# Evaluating the Efficacy of Artificial Intelligence-Driven Chatbots in Addressing Queries on Vernal Conjunctivitis

**DOI:** 10.7759/cureus.79688

**Published:** 2025-02-26

**Authors:** Muhammad Saad, Muhammad A Moqeet, Hassan Mansoor, Shama Khan, Rabia Sharif, Fahim Ullah Khan, Ali H Naqvi, Warda Ali

**Affiliations:** 1 Ophthalmology, Al-Shifa Trust Eye Hospital, Rawalpindi, PAK; 2 Cornea and Refractive Surgery, Al-Shifa Trust Eye Hospital, Rawalpindi, PAK; 3 Cornea, Al-Shifa Trust Eye Hospital, Rawalpindi, PAK

**Keywords:** artificial intelligence (ai), chatgpt, co-pilot, google gemini, health sciences, medical education, medical research, patient care

## Abstract

Background

Vernal keratoconjunctivitis (VKC) is a recurrent allergic eye disease that requires accurate patient education to ensure proper management. AI-driven chatbots, such as Google Gemini Advanced (Mountain View, California, US), are increasingly being explored as potential tools for providing medical information. This study evaluates the accuracy, reliability, and clinical applicability of Google Gemini Advanced in addressing VKC-related queries.

Objective

To assess the performance of Google Gemini Advanced in delivering medically accurate and relevant information about VKC and to evaluate its reliability based on expert ratings.

Methods

A total of 125 responses generated by Google Gemini Advanced for 25 VKC-related questions were assessed by two independent cornea specialists. Responses were rated on accuracy, completeness, and potential harm using a 5-point Likert scale (1-5). Inter-rater reliability was measured using Cronbach’s alpha. Responses were categorized into highly accurate (score of 5), minor inconsistencies (score of 4), and inaccurate (scores 1-3).

Results

Google Gemini Advanced demonstrated high inter-rater reliability (Cronbach’s alpha = 0.92, 95% CI: 0.87-0.94). Of the 125 responses, 108 (86.4%) were rated highly accurate (score of 5) while 17 (13.6%) had minor inconsistencies (score of 4) but posed no potential for harm. No responses were classified as inaccurate or potentially harmful. The combined mean score was 4.88 ± 0.31, reflecting strong agreement between raters. The chatbot consistently provided reliable information across diagnostic, treatment, and prognosis-related queries, with minor gaps in complex grading and treatment-related discussions.

Discussion

The findings support the use of AI-driven chatbots like Google Gemini Advanced as potential tools for patient education in ophthalmology. The chatbot exhibited strong accuracy and consistency, particularly in addressing general VKC-related queries. However, areas for improvement remain, especially in providing detailed guidance on treatment protocols and ensuring completeness in responses to complex clinical questions.

Conclusion

Google Gemini Advanced demonstrates high reliability and accuracy in delivering medical information about VKC, making it a valuable tool for patient education. While its responses are consistent and generally accurate, expert oversight remains necessary to refine AI-generated content for clinical applications. Further research is needed to enhance AI-driven chatbots' ability to provide nuanced medical advice and integrate them safely into ophthalmic patient education and clinical decision-making.

## Introduction

The advent of artificial intelligence (AI) has ushered in a transformative era across various sectors, with healthcare being a prominent beneficiary. Within healthcare, ophthalmology stands as a field ripe for AI integration, given its reliance on image interpretation and data analysis. The potential of AI to augment diagnostic accuracy, streamline workflows, and enhance patient care has garnered significant attention [1]. AI chatbots, trained on vast medical datasets, have the potential to bridge gaps in patient education by providing quick and accessible medical information. However, the reliability and accuracy of AI-generated responses remain critical concerns, particularly in sensitive areas such as clinical decision-making and treatment guidance. This study aims to evaluate the effectiveness of Google Gemini Advanced (Google LLC, Mountain View, California, US), an AI-driven chatbot, in delivering accurate and relevant responses to vernal keratoconjunctivitis (VKC)-related queries. By assessing its performance against expert evaluations, this research seeks to determine whether AI chatbots can serve as reliable tools for patient education in ophthalmology and identify areas for refinement to enhance their clinical utility.

Traditionally, the assessment of clinical knowledge has relied on human graders, often domain experts, to evaluate responses to questions. This process, while valuable, can be time-consuming, resource-intensive, and subject to inter-rater variability. The emergence of AI-driven chatbots, such as Google Gemini Advanced, has opened up new avenues for automating and improving the grading process. These chatbots, trained on vast datasets of text and code, have demonstrated the ability to understand and generate human-like text, making them suitable candidates for evaluating responses to clinical knowledge assessment questions [2].

VKC is a chronic and recurrent condition that primarily affects children, with substantial psychological and socioeconomic implications. Early diagnosis and prompt initiation of treatment tailored to the severity of VKC are essential for preventing vision loss and enhancing the quality of life for children and adults affected by the disease [3].

The use of AI-driven chatbots in grading responses on platforms like the VKC knowledge assessment tool holds the promise of several advantages. First, it could significantly expedite the grading process, providing timely feedback to learners and educators. Second, it could potentially reduce the burden on human graders, allowing them to focus on more complex tasks. Third, if developed and trained appropriately, AI-driven chatbots could offer consistent and objective grading, minimizing the impact of inter-rater variability.

Google Gemini Advanced and ChatGPT, developed by tech giants Google and OpenAI (San Francisco, California, US), respectively, are AI-driven chatbots capable of processing and generating human-like text. Despite their shared abilities, they differ in key aspects. Their training datasets vary, potentially influencing their knowledge bases and response styles. The underlying architecture and algorithms of the models also differ, which could affect their performance on specific tasks such as answering medical queries. Regarding access, ChatGPT has been publicly available for a more extended period, while Google Gemini Advanced is relatively newer with potentially evolving capabilities [4].

In light of these considerations and prior research, this study aims to evaluate the efficacy of the AI-driven chatbot, Google Gemini Advanced, in grading responses on the VKC knowledge assessment tool. By comparing the performance of Google Gemini Advanced with human expert graders, this research sheds light on AI's potential benefits and limitations in this domain. The main objective of this study was to evaluate the reliability, accuracy, and clinical applicability of Google Gemini Advanced in providing patient education on VKC, with a specific focus on assessing expert agreement and identifying areas for improvement in AI-generated medical responses.

## Materials and methods

This study evaluated the efficacy of Google Gemini Advanced, an AI-driven chatbot, in grading responses to questions on the VKC knowledge assessment tool. The methodology was adapted from previous studies that evaluated the accuracy of AI chatbots by comparing their responses to those provided by human experts in medical settings. To ensure a rigorous evaluation of Google Gemini Advanced, this study incorporated a structured assessment framework guided by expert raters with specialized knowledge in corneal diseases and VKC. The chatbot's responses were evaluated by two independent cornea specialists, each with extensive clinical and research experience in ophthalmology. These experts were provided with predefined assessment rubrics based on prior studies, ensuring standardized evaluation criteria across all responses. The evaluation process was conducted using a five-point Likert scale, measuring accuracy, relevance, completeness, and potential harm. To enhance consistency and minimize bias, both raters underwent a calibration phase, where they independently reviewed and discussed sample responses before formally assessing the full dataset. Additionally, while the chatbot’s training dataset was not explicitly disclosed by the developers, Google Gemini Advanced is trained on large-scale medical literature, clinical guidelines, and patient interaction models, allowing it to generate contextually relevant responses. Although this study primarily focused on general VKC-related patient inquiries, future research could extend the evaluation to assess the chatbot’s adaptability across different patient demographics, literacy levels, and cultural variations, ensuring a more comprehensive understanding of its responsiveness in diverse healthcare settings. The duly-formatted questionnaire used for evaluating VKC-related responses is included in Appendix 1.

The 25 questions used in this study were derived from a previous study in which the accuracy of ChatGPT responses to standard patient and parent questions on VKC was evaluated [5]. Two experienced clinical experts on VKC formulated these questions and covered four categories: general questions/etiology, prognosis, treatment/prevention, and allergy-related.

To assess the range of responses generated by Google Gemini Advanced, each question was input into the chatbot 5 times, resulting in 125 responses (5 responses per question x 25 questions). The chatbot was prompted to respond as if it were communicating with a patient or parent seeking information about VKC. The file containing all 125 responses generated by Google Gemini Advanced has been included and cited in Appendix 2.

The 125 responses generated by Google Gemini Advanced were independently evaluated by two cornea specialist surgeons using a 5-point Likert scale. This evaluation method aligns with approaches utilized in previous studies [1,2]. The definitions and criteria for the Likert scale are outlined in Table 1.

**Table 1:** Likert scale for assessing the accuracy and relevance of chatbot responses

Score	Definition	Explanation
5	Very Good	No inaccuracies, response is highly relevant, accurate, and poses no harm
4	Good	Minor inconsistencies present, but response is still relevant and poses no potential for harm
3	Moderate	Inaccuracies or inconsistencies that could be misinterpreted but do not cause significant harm
2	Poor	Contains potentially harmful inaccuracies that could mislead or harm the user
1	Very Poor	Response is irrelevant, inaccurate, or has significant potential for harm

The two experts assessed each response based on its relevance, accuracy, and potential for harm. Responses that were irrelevant or contained significant inaccuracies with the potential to harm were assigned lower scores. In contrast, relevant, accurate responses that did not pose any risk were assigned higher scores. Low scores (1-3) reflect responses ranging from very poor with unacceptable inaccuracies (1) to moderate with potentially misinterpretable inaccuracies (3). High scores (4-5) indicate responses that are good with only minor non-harmful inaccuracies (4) or very good with no inaccuracies (5). This scale helps categorize the responses' relevance, accuracy, and safety.

To ensure consistency in the evaluation process, inter-rater reliability between the two experts was assessed using Cronbach's alpha. This statistical measure determines the internal consistency of the ratings and indicates the degree of agreement between the raters. The scores assigned by the two experts for each response were then combined and summarized using descriptive statistics. For each question, the mean and standard deviation of the combined scores were calculated to provide an overall assessment of the chatbot's performance on that particular question.

## Results

Inter-rater reliability and overall performance

**Figure 1:**
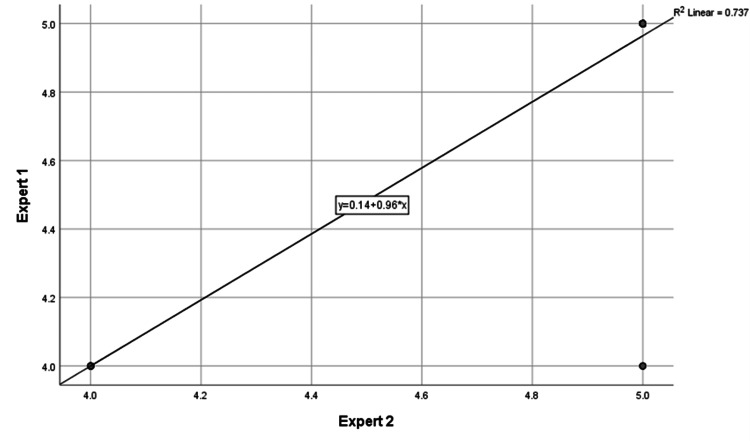
Correlation plot showing the relationship between scores assigned by Expert 1 and Expert 2

A total of 125 responses were generated for 25 VKC-related questions. Among these, 108 (86.4%) responses were rated as entirely relevant and free from inaccuracies, earning a perfect score of 5 from both experts. Seventeen responses (13.6%) were rated 4 due to minor inconsistencies or missing key details. No responses were rated below 4, indicating that none posed any significant inaccuracies or potential harm. The inter-rater reliability between the two expert evaluators was excellent, with a Cronbach’s alpha of 0.92 and a 95% confidence interval for the intraclass correlation coefficient ranging from 0.87 to 0.94. Figure 1 shows the correlation between the scores assigned by the two experts. The plot indicates a linear relationship, with an R² value of 0.737, supporting the high level of agreement between evaluators.

This strong correlation underscores the high level of agreement between the evaluators, indicating consistent and reliable assessment of the chatbot’s responses. The linear trend suggests that both experts consistently rated the accuracy and relevance of the responses in a similar manner, further validating the robustness of the evaluation process.

Performance analysis across questions

Table 2 summarizes the individual expert ratings, presented as median and range values, along with the combined mean ± standard deviation (SD) scores for each question, highlighting the high level of agreement between the two experts.

**Table 2:** Individual expert scores in median and range, and combined scores in mean ± SD SD: Standard Deviation

Question	Expert 1 (Median (Range))	Expert 2 (Median (Range))	Combined (Mean ± SD)
1	5 (5–5)	5 (5–5)	5.0 ± 0.00
2	5 (5–5)	5 (5–5)	5.0 ± 0.00
3	4 (4–5)	5 (4–5)	4.6 ± 0.41
4	5 (5–5)	5 (5–5)	5.0 ± 0.00
5	5 (4–5)	5 (4–5)	4.6 ± 0.54
6	5 (5–5)	5 (5–5)	5.0 ± 0.00
7	5 (4–5)	5 (4–5)	4.8 ± 0.44
8	5 (4–5)	5 (4–5)	4.8 ± 0.44
9	5 (4–5)	5 (4–5)	4.8 ± 0.44
10	5 (4–5)	5 (4–5)	4.8 ± 0.44
11	5 (5–5)	5 (5–5)	5.0 ± 0.00
12	5 (4–5)	5 (4–5)	4.6 ± 0.54
13	5 (5–5)	5 (5–5)	5.0 ± 0.00
14	5 (4–5)	5 (4–5)	4.6 ± 0.54
15	5 (5–5)	5 (5–5)	5.0 ± 0.00
16	5 (5–5)	5 (5–5)	5.0 ± 0.00
17	5 (4–5)	5 (5–5)	4.9 ± 0.22
18	5 (4–5)	5 (5–5)	4.9 ± 0.22
19	5 (4–5)	5 (4–5)	4.8 ± 0.44
20	5 (5–5)	5 (5–5)	5.0 ± 0.00
21	5 (5–5)	5 (5–5)	5.0 ± 0.00
22	5 (5–5)	5 (5–5)	5.0 ± 0.00
23	5 (5–5)	5 (5–5)	5.0 ± 0.00
24	5 (5–5)	5 (5–5)	5.0 ± 0.00
25	5 (4–5)	5 (4–5)	4.8 ± 0.44

The evaluation of responses by Google Gemini Advanced demonstrates strong performance, with 17 out of 25 questions receiving perfect scores of 5 (5-5) from both cornea specialists. This highlights the chatbot’s ability to provide accurate and relevant information, particularly for straightforward VKC-related topics such as symptoms, prognosis, and general treatments. For eight questions, combined scores ranged from 4.6 to 4.9, indicating minor inconsistencies or omissions. These responses, while generally accurate, lacked depth or references to tools like the Bonini scale when addressing complex topics such as severity grading or advanced management strategies. Expert agreement was highest for consistently scored questions, reflecting the chatbot’s reliability in generating clear, unambiguous answers for common queries. Slight score variations between 4 and 5 emphasized the importance of expert oversight in identifying gaps, such as incomplete discussions of nuanced clinical scenarios.

**Figure 2:**
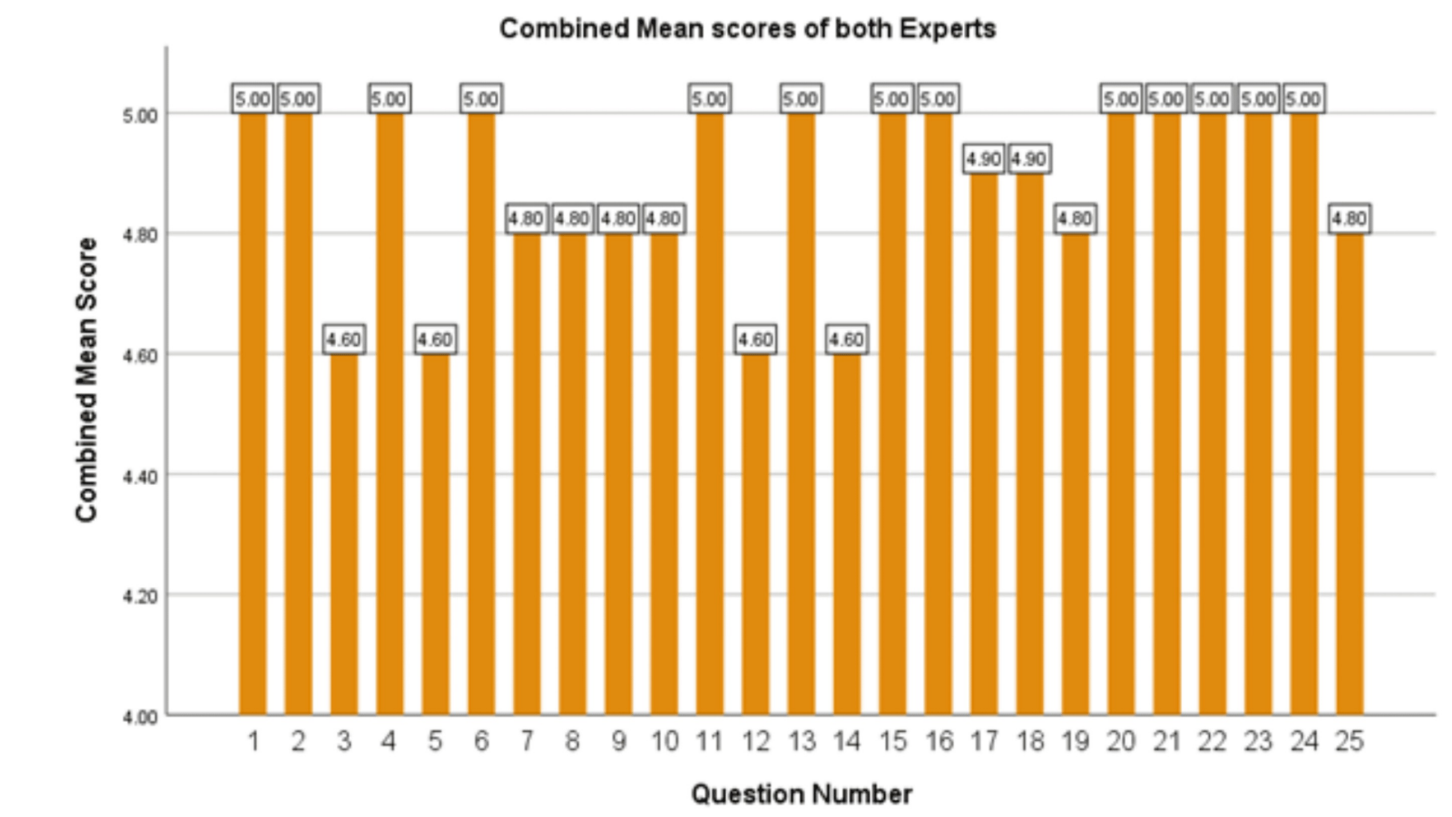
Bar chart depicting the combined mean scores assigned by both experts for each of the 25 VKC-related questions. VKC: vernal keratoconjunctivitis

Figure 2 presents the combined mean scores for the 25 questions, ranging from 4.6 to 5.0. The overall mean score across all responses was 4.88 ± 0.31.

The bar chart highlights the chatbot's overall strong performance, with the majority of the questions achieving scores close to 5, indicating a high degree of accuracy and relevance in its responses. The consistent pattern of high scores emphasizes the chatbot's reliability in maintaining a high standard of response quality across a diverse range of queries, further supporting its potential as a reliable tool for patient education. However, the slightly lower scores in specific areas suggest opportunities for improvement, particularly in addressing complex clinical scenarios.

High-Scoring Responses (Score = 5)

Responses with a score of 5 were deemed complete, accurate, and highly relevant. For example, the response to Question 4 ("Is it only children who get vernal keratoconjunctivitis?") was detailed and informative, correctly describing the prevalence across different age groups. Such responses demonstrated a robust understanding of VKC and provided an appropriate context for patient education.

Responses With Minor Inconsistencies (Score = 4)

Responses that scored 4 exhibited minor gaps in detail but remained accurate and safe. For instance, in response to Question 17 (“What is the spectrum of severity of VKC?”), while the chatbot provided a general overview of severity assessment, it did not mention the Bonini scale, a structured tool commonly referenced in VKC literature. Although the omission did not compromise the overall safety or relevance, it highlighted a gap in comprehensiveness.

Questions that received lower scores are discussed below, highlighting specific areas of inconsistency or incomplete information. 

Detailed analysis of lower scores

Questions 3 and 5 (Combined Mean Score = 4.6)

Question 3 (“Who gets VKC?”):The lower score was due to discrepancies in age range categorization (e.g., “1 to 22 years old” vs. “5 to 12 years old”) and oversimplification of gender disparities. Additionally, some responses lacked specific details on high-risk subgroups, such as individuals with atopic tendencies or those living in certain climates.

Question 5 ("How do I know if my child has VKC?"): The responses contain minor inaccuracies, such as mislabeling limbal nodules and overemphasizing crusting, and lack key diagnostic details, including the distinction between VKC forms, age/gender prevalence, associated atopic conditions, and corneal complications, which are crucial for accurate diagnosis and management.

Questions 7 to 10 (Combined Mean Score = 4.8)

These questions addressed treatment and prognosis. While most responses were accurate, they varied in depth.

Question 7 (“Can vernal keratoconjunctivitis be treated by a general ophthalmologist?"):Responses varied in comprehensiveness, with most correctly stating that general ophthalmologists can manage mild to moderate VKC, but only some detailed when referral to specialists is necessary for severe, unresponsive, or complicated cases involving corneal damage or scarring.

Question 8 (“What is the prognosis for vernal keratoconjunctivitis?”): Although responses mentioned that VKC is generally self-limiting, they provided limited details on persistent cases or potential complications like keratoconus.

Question 9 (“When will my kid’s vernal keratoconjunctivitis go away?"): The responses provide a general notion that VKC is self-limiting and resolves by puberty but lack precise timelines for varying severities, making them less actionable. They also insufficiently emphasize regular follow-up as a crucial strategy for monitoring progression and preventing complications.

Question 10 (“Can you get vernal keratoconjunctivitis as an adult?"):The responses on adult VKC inconsistently distinguish early-onset (persisting from childhood) from late-onset (developing de novo), with varying definitions, prevalence estimates, and clinical implications. This leads to confusion and gaps in understanding risk factors, causes, and prognosis.

Questions 12 and 14 (Combined Mean Score = 4.6)

Question 12 (“Can you go blind from vernal keratoconjunctivitis?”):While responses provided a general overview of VKC severity, they lacked references to widely used grading tools like the Bonini scale.

Question 14 (“What can I do to prevent vernal keratoconjunctivitis?"): The responses varied in detail, with some focusing on basic preventive measures and others mentioning advanced treatments like corticosteroids, mast cell stabilizers, and immunomodulators for severe or refractory VKC. This led to inconsistencies in the depth of guidance provided.

Questions 17 and 18 (Combined Mean Score = 4.9)

These questions received slightly lower scores due to minor gaps in detail. Examples follow.

Question 17 (“What is the spectrum of severity of vernal keratoconjunctivitis?"): The responses varied in comprehensiveness, with some omitting rare complications like corneal neovascularization, scarring, and permanent vision loss, while others detailed shield ulcers, macro erosions, and the need for close monitoring in severe cases.

Question 18 (“What eye drops should I get for vernal keratoconjunctivitis?"):Although management strategies were correctly covered, there was variability in discussing advanced treatments such as immunomodulators for severe cases.

Questions 19 and 25 (Combined Mean Score = 4.8)

Question 19 (“Can you get side effects from the eyedrops treating vernal keratoconjunctivitis?"): Inconsistencies were noted in the level of detail regarding environmental triggers and allergen management, as some responses thoroughly addressed specific strategies like pollen control, dust mite reduction, and pet dander management, while others only briefly mentioned these factors without offering clear, actionable steps. This variation in depth may lead to gaps in understanding how to minimize VKC flare-ups effectively.

Question 25 (“Can you use makeup if you have vernal keratoconjunctivitis?"): The responses lacked depth in discussing makeup-related precautions and alternatives for VKC patients, with varying levels of detail on minimizing risks such as using hypoallergenic products and maintaining hygiene. Additionally, not all responses emphasized the importance of consulting an ophthalmologist before using makeup, leading to a slightly lower score due to incomplete guidance.

Subgroup analysis

A subgroup analysis was conducted to evaluate the chatbot’s performance across different question categories, including diagnostic, treatment-related, and prognosis-based inquiries. The highest consistency was observed in diagnostic questions, where the chatbot demonstrated strong accuracy and reliability, with most responses receiving perfect scores. This suggests that Google Gemini Advanced effectively conveys factual information regarding VKC symptoms and general disease characteristics. Treatment-related questions, however, exhibited slightly more variability, with scores ranging between 4.6 and 5.0. Minor inconsistencies were identified in responses related to grading severity and treatment approaches, where the chatbot occasionally omitted references to structured tools like the Bonini scale. While these omissions did not introduce harmful inaccuracies, they indicated areas where responses could be refined to provide more comprehensive guidance. Prognosis-related questions showed similarly high scores, averaging 4.8 to 5.0, but responses addressing long-term disease outcomes and management strategies were occasionally less detailed than expected.​​​​​​​

Summary

Overall, responses with a score of 5 demonstrated high accuracy, relevance, and completeness. Responses with a score of 4 had minor informational gaps but were still considered accurate and safe for patient education. All responses were rated 4 or higher, indicating that none contained significant inaccuracies or posed any potential risk. Questions related to the diagnosis of VKC generally received higher scores, demonstrating the chatbot's ability to provide well-established and accurate clinical information. This consistency in delivering relevant diagnostic criteria contributed to the high overall performance in these areas. In contrast, questions addressing treatment strategies scored slightly lower due to variability in response depth and a lack of uniformity in discussing advanced management options such as immunomodulators or referral guidelines for severe cases. These discrepancies highlight the chatbot's limitations in covering complex treatment-related topics comprehensively.

## Discussion

This study revealed high inter-rater reliability (Cronbach’s alpha = 0.92) among the two experts evaluating Google Gemini Advanced's responses about VKC. Out of 125 responses, 108 were rated highly accurate and relevant (score of 5) while 17 had minor inconsistencies (score of 4) with no potential for harm. The mean combined score was 4.88 ± 0.31, indicating that the chatbot effectively provides reliable information on VKC, showcasing its potential as a valuable tool for patient education in ophthalmology.

Gemini Advanced has been trained on an even more extensive dataset of text and code than ChatGPT, further enhancing its ability to understand and generate human-like text, particularly in complex domains such as medical science [6]. This aligns with the findings of Potapenko et al. (2023), who explored AI responses for patient queries on optic disc drusen, and Rasmussen et al. (2023), who examined AI responses for VKC-related inquiries [5,7]. Both studies indicate that while AI can offer pertinent information, inaccuracies, and inconsistencies often arise, particularly concerning treatment recommendations.

Furthermore, this recent study supports the notion discussed by Alhur (2024), emphasizing the potential of AI in revolutionizing healthcare through personalized health coaching, early disease detection, and medical decision-making [8]. Gemini Advanced's high accuracy in answering complex medical queries underscores the importance of human oversight in AI applications to ensure accuracy and ethical considerations. AI's capacity to enhance patient engagement and support clinical decision-making highlights its role in ongoing healthcare innovations.

The study by Simon Høj et al.(2024) evaluated the quality of ChatGPT's information on allergic rhinitis using a Likert scale, finding 8 out of 20 responses completely accurate while others showed varying degrees of inaccuracies. This highlights that while AI can provide relevant information for patient education, it cannot replace the nuanced decision-making and empathy of healthcare professionals [9]. In contrast, our study emphasizes the greater effectiveness of AI-driven chatbots like Google Gemini Advanced in delivering accurate medical information, underscoring the potential for these technologies to enhance patient education and care when used with appropriate oversight.

Both this study and a recent analysis evaluating ChatGPT's performance in generating responses to patient and parent questions on vernal keratoconjunctivitis (VKC) have highlighted key similarities and differences in the capabilities of AI-driven chatbots for patient education [5]. In the ChatGPT study, responses were generally relevant and user-friendly; however, significant concerns were raised regarding the accuracy and completeness of treatment-related information. ChatGPT frequently omitted critical therapies, such as calcineurin inhibitors, and failed to mention potential serious side effects of corticosteroids, which resulted in lower scores for treatment and prevention questions. Additionally, while ChatGPT provided reasonable responses to general questions and prognosis-related queries, its suggestions in complex areas, such as surgical options for VKC, were flagged as potentially harmful by the experts evaluating its responses. In contrast, our study, which focused on Google Gemini Advanced, demonstrated more consistent and reliable responses across all categories, particularly in treatment and prevention. Unlike ChatGPT, Gemini provided comprehensive answers, consistently mentioned key treatment options, and emphasized the need for professional medical consultation when addressing adverse effects or complex interventions, reducing the potential risk of harmful inaccuracies. The differences in performance between the two chatbots may be attributed to variations in their underlying architecture and training datasets. Google Gemini Advanced appears to be better optimized for delivering precise and reliable content in specialized medical domains like ophthalmology. Both studies underline the importance of continued monitoring and evaluation of AI tools to ensure their reliability, primarily when used for critical health information. While ChatGPT's ease of accessibility and readability make it appealing for general patient education, its occasional inaccuracies necessitate cautious use in medical contexts. On the other hand, Google Gemini Advanced shows incredible promise as a dependable tool for ophthalmic patient education, highlighting its potential for broader application in healthcare. Future research could benefit from a direct head-to-head comparison of these two AI models across a broader range of medical topics to comprehensively evaluate their unique strengths and limitations.

While the findings of this study are promising, several limitations deserve consideration. The integration of AI in grading responses raises significant concerns regarding the accuracy and reliability of these systems, which require thorough evaluation. There is a potential for biases in the training data and algorithms, leading to unfair or inaccurate assessments that could negatively impact patient care. Additionally, the "black box" nature of some AI models can obscure the reasoning behind their grading decisions, hindering transparency and accountability (Tikhomirov et al., 2024) [10]. This lack of interpretability raises ethical issues about relying on AI for clinical decision-making, mainly when patient safety is critical.

## Conclusions

In conclusion, this study highlights the efficacy of Google Gemini Advanced in delivering accurate and relevant information on vernal keratoconjunctivitis (VKC) to patients and parents. The chatbot’s high inter-rater reliability and consistently strong performance across a range of VKC-related queries demonstrate its potential as a reliable educational tool in ophthalmology. While the chatbot excels in providing objective, consistent, and rapid information, it lacks the personalized nuance, empathy, and clinical judgment that only human clinicians can offer. This underscores AI-driven chatbots' complementary role in patient education rather than replacing direct medical consultations.

However, for AI to be safely and effectively integrated into healthcare, further clinical validation and real-world trials are necessary to assess its impact on diverse patient populations and clinical workflows. Ethical concerns, such as data privacy, informed consent, and algorithmic bias, must be carefully addressed to maintain public trust in AI-driven medical tools. Regulatory frameworks should ensure that AI-generated content is accurate, unbiased, and patient-centered. As AI continues to evolve, ongoing refinement, oversight, and interdisciplinary collaboration will be essential to unlock its full potential in enhancing patient education, improving accessibility, and supporting better healthcare outcomes.

## Appendices

Appendix 1

The questionnaire used for evaluating Google Gemini Advanced’s responses to vernal keratoconjunctivitis (VKC) queries as shown in Table 2.

**Table 3:** Likert response scale table Instruction: For each response given in the booklet, circle the option that best characterizes how you feel about the statement where: 1 = Strongly Disagree, 2 = Disagree, 3 = Neither Agree nor Disagree, 4 = Agree, 5 = Strongly Agree

Q 1 a	1	2	3	4	5
Q 1 b	1	2	3	4	5
Q 1 c	1	2	3	4	5
Q 1 d	1	2	3	4	5
Q 1 e	1	2	3	4	5
Q 2 a	1	2	3	4	5
Q 2 b	1	2	3	4	5
Q 2 c	1	2	3	4	5
Q 2 d	1	2	3	4	5
Q 2 e	1	2	3	4	5
Q 3 a	1	2	3	4	5
Q 3 b	1	2	3	4	5
Q 3 c	1	2	3	4	5
Q 3 d	1	2	3	4	5
Q 3 e	1	2	3	4	5
Q 4 a	1	2	3	4	5
Q 4 b	1	2	3	4	5
Q 4 c	1	2	3	4	5
Q 4 d	1	2	3	4	5
Q 4 e	1	2	3	4	5
Q 5 a	1	2	3	4	5
Q 5 b	1	2	3	4	5
Q 5 c	1	2	3	4	5
Q 5 d	1	2	3	4	5
Q 5 e	1	2	3	4	5
Q 6 a	1	2	3	4	5
Q 6 b	1	2	3	4	5
Q 6 c	1	2	3	4	5
Q 6 d	1	2	3	4	5
Q 6 e	1	2	3	4	5
Q 7 a	1	2	3	4	5
Q 7 b	1	2	3	4	5
Q 7 c	1	2	3	4	5
Q 7 d	1	2	3	4	5
Q 7 e	1	2	3	4	5
Q 8 a	1	2	3	4	5
Q 8 b	1	2	3	4	5
Q 8 c	1	2	3	4	5
Q 8 d	1	2	3	4	5
Q 8 e	1	2	3	4	5
Q 9 a	1	2	3	4	5
Q 9 b	1	2	3	4	5
Q 9 c	1	2	3	4	5
Q 9 d	1	2	3	4	5
Q 9 e	1	2	3	4	5
Q 10 a	1	2	3	4	5
Q 10 b	1	2	3	4	5
Q 10 c	1	2	3	4	5
Q 10 d	1	2	3	4	5
Q 10 e	1	2	3	4	5

Q 11 a	1	2	3	4	5
Q 11 b	1	2	3	4	5
Q 11 c	1	2	3	4	5
Q 11 d	1	2	3	4	5
Q 11 e	1	2	3	4	5
Q 12 a	1	2	3	4	5
Q 12 b	1	2	3	4	5
Q 12 c	1	2	3	4	5
Q 12 d	1	2	3	4	5
Q 12 e	1	2	3	4	5
Q 13 a	1	2	3	4	5
Q 13 b	1	2	3	4	5
Q 13 c	1	2	3	4	5
Q 13 d	1	2	3	4	5
Q 13 e	1	2	3	4	5
Q 14 a	1	2	3	4	5
Q 14 b	1	2	3	4	5
Q 14 c	1	2	3	4	5
Q 14 d	1	2	3	4	5
Q 14 e	1	2	3	4	5
Q 15 a	1	2	3	4	5
Q 15 b	1	2	3	4	5
Q 15 c	1	2	3	4	5
Q 15 d	1	2	3	4	5
Q 15 e	1	2	3	4	5
Q 16 a	1	2	3	4	5
Q 16 b	1	2	3	4	5
Q 16 c	1	2	3	4	5
Q 16 d	1	2	3	4	5
Q 16 e	1	2	3	4	5
Q 17 a	1	2	3	4	5
Q 17 b	1	2	3	4	5
Q 17 c	1	2	3	4	5
Q 17 d	1	2	3	4	5
Q 17 e	1	2	3	4	5
Q 18 a	1	2	3	4	5
Q 18 b	1	2	3	4	5
Q 18 c	1	2	3	4	5
Q 18 d	1	2	3	4	5
Q 18 e	1	2	3	4	5
Q 19 a	1	2	3	4	5
Q 19 b	1	2	3	4	5
Q 19 c	1	2	3	4	5
Q 19 d	1	2	3	4	5
Q 19 e	1	2	3	4	5
Q 20 a	1	2	3	4	5
Q 20 b	1	2	3	4	5
Q 20 c	1	2	3	4	5
Q 20 d	1	2	3	4	5
Q 20 e	1	2	3	4	5

Q 22 a	1	2	3	4	5
Q 22 b	1	2	3	4	5
Q 22 c	1	2	3	4	5
Q 22 d	1	2	3	4	5
Q 22 e	1	2	3	4	5
Q 23 a	1	2	3	4	5
Q 23 b	1	2	3	4	5
Q 23 c	1	2	3	4	5
Q 23 d	1	2	3	4	5
Q 23 e	1	2	3	4	5
Q 24 a	1	2	3	4	5
Q 24 b	1	2	3	4	5
Q 24 c	1	2	3	4	5
Q 24 d	1	2	3	4	5
Q 24 e	1	2	3	4	5
Q 25 a	1	2	3	4	5
Q 25 b	1	2	3	4	5
Q 25 c	1	2	3	4	5
Q 25 d	1	2	3	4	5
Q 25 e	1	2	3	4	5

Appendix 2

A detailed file containing the 125 responses generated by Google Gemini Advanced for the 25 questions listed in Appendix 1. Each question was input five times into the chatbot to assess consistency, accuracy, and relevance as shown in Table 3. The file was used by expert raters for evaluation purposes.

**Table 4:** Complete dataset of 125 responses generated by Google Gemini Advanced, with 5 responses provided for each of the 25 questions

Question	Response Generated by Gemini AI
1	What is vernal keratoconjunctivitis?	Vernal keratoconjunctivitis (VKC), also known as spring catarrh, is a chronic, allergic eye disease that primarily affects young boys. It is characterized by intense itching, tearing, and light sensitivity. The symptoms typically worsen during the spring and summer months, and may improve or disappear completely in the winter.
		Vernal keratoconjunctivitis (VKC), also known as spring catarrh, is a chronic, allergic eye disease that primarily affects young boys. It is characterized by inflammation of the conjunctiva, the thin layer of tissue that covers the white part of the eye and the inner surface of the eyelids.
		Vernal keratoconjunctivitis (VKC), also known as spring catarrh, is a chronic, allergic eye disease that primarily affects young boys. It is characterized by inflammation of the conjunctiva, the clear tissue that covers the white part of the eye and the inner surface of the eyelids.
		Vernal keratoconjunctivitis (VKC), also known as spring catarrh, is a chronic, allergic eye disease that primarily affects young boys, typically between the ages of 5 and 12. It is characterized by intense itching, photophobia (light sensitivity), and tearing. The symptoms of VKC are often worse during the spring and summer months, and may improve in the fall and winter. This is because VKC is often triggered by allergens such as pollen, dust mites, and mold.
		Vernal keratoconjunctivitis (VKC), also known as spring catarrh, is a chronic, allergic eye disease that primarily affects young boys, typically between the ages of 5 and 12. It is characterized by intense itching, tearing, light sensitivity (photophobia), and a thick, ropy discharge from the eyes.
2	Why did my child get vernal keratoconjunctivitis?	Unfortunately, it's impossible for me to definitively say why your child specifically developed vernal keratoconjunctivitis (VKC) without a full medical evaluation. Diagnosing the root cause of VKC is challenging for medical professionals as well. However, I can share some general information about the risk factors associated with VKC: Age and gender: VKC primarily affects young boys between the ages of 5 and 12, although it can occur in older children and adults of both genders. Family history of allergies: Children with a family history of allergies such as asthma, eczema, or allergic rhinitis are at a higher risk of developing VKC. Geographical location: VKC is more common in hot, dry climates such as the Mediterranean region, the Middle East, and Southeast Asia. Exposure to allergens: While the exact triggers of VKC are unknown, it's believed to be an allergic reaction. Exposure to common allergens like pollen, dust mites, pet dander, and mold may increase the risk of flare-ups. It's important to remember that these are just general risk factors, and having one or more of them doesn't guarantee that your child will develop VKC. Conversely, a child without any of these risk factors can still develop the condition. If you're concerned about your child's VKC, the best course of action is to consult with an ophthalmologist (eye doctor). They can perform a comprehensive examination, discuss your child's specific situation, and recommend the most appropriate treatment plan.
		Unfortunately, I cannot definitively tell you why your child specifically developed vernal keratoconjunctivitis (VKC) as this requires individual medical evaluation. However, I can share some general information about factors that may increase the risk: Allergic tendencies: Children with a personal or family history of allergies or atopic conditions like eczema or asthma are more susceptible to VKC. This suggests a potential role of the immune system in overreacting to harmless substances like pollen or dust mites. Environment: The prevalence of VKC is higher in hot, arid regions like the Mediterranean, West Africa, and the Indian subcontinent. This suggests environmental factors, possibly related to increased exposure to allergens or irritants, may play a role. Genetics: While the exact genetic link isn't fully understood, research suggests a potential genetic predisposition in some individuals. It's important to understand that these are just potential contributing factors, and the exact cause of VKC in any individual case remains unknown. Consulting a healthcare professional: If you're concerned about your child's VKC, it's crucial to consult an ophthalmologist (eye doctor) for a proper diagnosis and discuss the most effective treatment plan for your child's specific case. They can also help identify potential triggers and provide guidance on managing the condition and preventing future flare-ups.
		Unfortunately, I cannot provide specific medical advice or diagnose your child's condition. However, I can share some general information about the potential causes of VKC: Allergies: VKC is primarily considered an allergic eye disease. While the exact allergens triggering VKC are not always identified, common triggers include pollen, dust mites, and pet dander. Genetics: A family history of allergies, including atopic dermatitis (eczema) and asthma, increases the risk of developing VKC. Environmental factors: VKC is more prevalent in hot, dry climates and may be exacerbated by exposure to smoke, wind, and other irritants. It's important to consult with your child's pediatrician or ophthalmologist to understand the specific cause of your child's VKC and develop a personalized treatment plan. They can perform a thorough examination, discuss your child's medical history, and determine the best course of action.
		Unfortunately, it's impossible for me to definitively say why your child specifically developed vernal keratoconjunctivitis (VKC) without a full medical evaluation. However, I can share some general information about the potential causes and risk factors: Allergic reaction: VKC is primarily considered an allergic reaction in the eyes. While the exact cause of the allergy remains under research, it's believed to be triggered by exposure to various allergens, such as: Pollen Dust mites Mold Animal dander Increased risk factors: Certain factors might increase the susceptibility to VKC, including: Age: VKC typically affects children between 5 and 12 years old, though it can occur in adults as well. Gender: VKC is more prevalent in boys than in girls. Family history: Having a family history of allergies or conditions like atopic dermatitis (eczema) or asthma increases the risk. Geographic location: VKC is more common in hot, dry climates like the Mediterranean region, West Africa, and the Indian subcontinent. It's important to remember that these are just general factors, and individual cases may vary. If you're concerned about your child's VKC, it's crucial to consult with a pediatric ophthalmologist for a proper diagnosis and discuss the most suitable treatment plan. They can also help identify any specific triggers and provide guidance on reducing exposure to them.
		Unfortunately, I cannot provide specific medical advice or diagnose your child's condition. The exact reason why your child developed vernal keratoconjunctivitis (VKC) is best determined by a qualified medical professional, like your child's pediatrician or ophthalmologist (eye doctor). However, I can share some general information about factors that may contribute to VKC: Allergies: VKC is primarily considered an allergic reaction, and children with a family history of allergies (such as asthma or eczema) are more likely to develop it. Age: VKC generally affects children and young adults, typically between the ages of 5 and 12. Geography and climate: The condition is more prevalent in hot, dry climates and regions with higher levels of air pollution. Genetics: While not fully understood, there is a suspected genetic component, as VKC can run in families. Remember, this is just general information, and it's crucial to consult your child's doctor to understand the specific reason behind their case and get the most effective treatment plan.
3	Who gets vernal keratoconjunctivitis?	While anyone can technically develop vernal keratoconjunctivitis (VKC), certain groups are at higher risk than others: Age and gender: VKC primarily affects young boys between the ages of 5 and 12, although it can occur in older children, adults, and females, with the prevalence becoming more equal with increasing age. Family history: Children with a family history of allergies like asthma, eczema, or allergic rhinitis are at an increased risk. Geography: VKC is more common in hot, dry climates such as the Mediterranean region, the Middle East, and Southeast Asia. Allergen exposure: While the exact triggers are unknown, VKC is believed to be an allergic reaction. Exposure to common allergens like pollen, dust mites, pet dander, and mold may increase the risk of flare-ups. It's important to remember that these are just risk factors, and having one or more of them doesn't guarantee that someone will develop VKC. Conversely, someone without any of these risk factors can still develop the condition. If you have further questions or concerns about VKC, it's always recommended to consult with an ophthalmologist (eye doctor) for personalized advice and diagnosis.
		Vernal keratoconjunctivitis (VKC) primarily affects young boys between the ages of 1 and 22 years old, with a peak occurrence around 6-7 years old. However, it is important to remember that anyone can develop VKC, although it's less common in: Females: While boys are affected more frequently, the gap in prevalence narrows as individuals age. Adults: VKC often resolves or becomes less severe after puberty. Although, it can persist or even develop for the first time in adults. Individuals in specific climates: Though VKC has been historically associated with hot and dry climates, it can occur anywhere and is not strictly seasonal in all cases. Here are some additional points to consider: Family history: Individuals with a family history of allergies or atopic conditions like eczema or asthma are at an increased risk for developing VKC. Geographic variations: The prevalence of VKC can vary significantly depending on geographic location and environmental factors. If you are concerned about yourself or someone you know potentially having VKC, it is crucial to consult an ophthalmologist (eye doctor) for a proper diagnosis and appropriate treatment plan.
		Vernal keratoconjunctivitis (VKC) primarily affects young males, typically between the ages of 1 and 22. While it can occur in both genders, males are affected 2-4 times more frequently than females, especially in childhood. Here's a breakdown of who is more likely to get VKC: Age: Most commonly diagnosed between ages 1-22, with symptoms often resolving by puberty. Gender: Males are significantly more affected than females, especially in childhood. The gender difference becomes smaller with age. Family history: Having a family history of allergies, particularly atopic dermatitis (eczema) and asthma, increases the risk of developing VKC. Climate: VKC is more prevalent in hot, dry climates and might be exacerbated by exposure to irritants like smoke, wind, and dust. It's important to note that these are general trends and VKC can occur in individuals outside these categories. If you have any concerns about yourself or your child potentially having VKC, consulting a qualified healthcare professional is crucial for proper diagnosis and treatment.
		Vernal keratoconjunctivitis (VKC) primarily affects young males, with the highest prevalence occurring between the ages of 5 and 12 years. However, it's important to note that: It can affect individuals of any age and gender: While less common, VKC can occur in adults, females, and even infants. The prevalence of VKC among females increases with age, narrowing the gender gap in adulthood. Geographic location plays a role: VKC is more prevalent in hot, dry climates like the Mediterranean region, parts of Africa, and the Indian subcontinent. This suggests environmental factors like exposure to specific allergens or irritants might contribute to the development of VKC. Atopic conditions increase susceptibility: Individuals with a personal or family history of atopic conditions like eczema, asthma, or allergic rhinitis are at a higher risk of developing VKC. This suggests a potential link between VKC and the body's overall allergic response. Therefore, while VKC is most commonly seen in young boys, it's not exclusive to this demographic. Consulting a healthcare professional for diagnosis and management is crucial for anyone experiencing symptoms suggestive of VKC, regardless of age or gender.
		While anyone can develop vernal keratoconjunctivitis (VKC), certain groups are more susceptible than others: Age: VKC primarily affects young males between the ages of 5 and 12, though it can occur in older children and young adults. Gender: Boys are affected more often than girls, with the ratio being around 3:1. However, the gender gap narrows with age. Allergies: Individuals with a family history of allergies, such as asthma, eczema, or allergic rhinitis, are at increased risk of developing VKC. Geography and Climate: VKC is more prevalent in hot, dry climates and regions with higher air pollution levels. Genetics: Though not fully understood, there is a suspected genetic component, meaning a family history of VKC can increase the risk. It's important to remember that these are just general trends, and VKC can still occur in individuals who don't fall into these categories. If you suspect you or someone you know might have VKC, it's crucial to consult an ophthalmologist (eye doctor) for proper diagnosis and treatment.
4	Is it only children who get vernal keratoconjunctivitis?	No, vernal keratoconjunctivitis (VKC) is not exclusive to children. While it primarily affects young boys between the ages of 5 and 12, it can occur in older children, adolescents, and even adults in rarer cases. Here's a breakdown of the occurrence of VKC across different age groups: Most common: Children between 5 and 12 years old, particularly boys. Less common: Teenagers and young adults. Rare: Adults beyond young adulthood. It's important to note that VKC in adults is a distinct entity compared to the childhood form. Adult VKC is considered less common and often doesn't follow the same self-limiting course as childhood VKC, meaning it might persist for longer periods and require different treatment approaches. If you or someone you know experiences symptoms suggestive of VKC, regardless of age, it's crucial to consult an ophthalmologist (eye doctor) for proper diagnosis and personalized treatment.
		No, vernal keratoconjunctivitis (VKC) is not exclusive to children, although it is most common in young boys between the ages of 5 and 12. While less frequent, it can occur in: Adults: Although rare, VKC can persist or develop for the first time in adults. This is categorized as adult VKC, which has different characteristics and treatment approaches compared to childhood VKC. Females: Although less prevalent than in males, VKC can also affect females, especially in adulthood. The frequency of VKC in females increases with age, making the gender gap less significant in adults. Infants: While very uncommon, VKC can even occur in infants, although diagnosis can be challenging due to the difficulty in expressing symptoms. It's important to remember that specific factors like allergic predisposition, environmental triggers, and geographical location can influence the development of VKC, regardless of age or gender. If you or someone you know experiences symptoms suggestive of VKC, it's crucial to consult a healthcare professional for a proper diagnosis and discuss the most suitable treatment plan.
		While vernal keratoconjunctivitis (VKC) is primarily a disease affecting children, it's important to clarify that it can occur in adults, although less commonly. Here's a breakdown of the occurrence of VKC in different age groups: Most common: Ages 1-22, with symptoms often resolving by puberty. Less common: Adults. Estimates suggest that around 12% of VKC cases may persist into adulthood. Additionally, a rarer form of VKC can arise de novo (for the first time) in adults with no prior childhood history. It's crucial to remember that this information is for general understanding and cannot be used for self-diagnosis. If you or someone you know experiences symptoms potentially consistent with VKC, regardless of age, it's essential to consult a healthcare professional for proper diagnosis and treatment.
		While vernal keratoconjunctivitis (VKC) is much more common in children, it's important to understand that adults can also develop the condition. Here's a breakdown: Predominantly affects children: Age range: Primarily affects young boys between 1 and 22 years old, with a peak around 6-7 years old. Resolution: Often resolves or becomes less severe after puberty. However, adults can also be affected: Adult VKC: Although less common, VKC can persist beyond childhood or even develop for the first time in adults. This is categorized as adult-onset VKC. Two variants: Adult VKC can be further categorized: Early-onset: Childhood VKC persisting beyond puberty. Late-onset: VKC arising de novo (for the first time) in adults. Additional points: Gender disparity: While boys are more frequently affected as children, the gap narrows in adults. Geographic variations: More prevalent in hot and dry climates, but can occur anywhere. If you suspect VKC in yourself or someone you know, regardless of age, consult an ophthalmologist (eye doctor) for accurate diagnosis and appropriate treatment.
		No, vernal keratoconjunctivitis (VKC) is not exclusive to children, although it primarily affects them. Here's a breakdown of who can develop VKC: Most common: Young boys: VKC is most prevalent in boys between the ages of 5 and 12. Less common, but possible: Older children and adults: While less frequent, VKC can occur in older children, adolescents, and even adults. Females: Although less common than in males, females can also develop VKC, with the prevalence becoming more equal with increasing age. It's important to note that: Adults with VKC: When VKC persists into adulthood, it's often referred to as adult VKC. This form is considered rare and may differ slightly in its presentation and management compared to the typical childhood VKC. Prevalence: Although VKC can technically affect anyone, it's still considered a relatively uncommon condition overall, regardless of age or gender. If you have any concerns about yourself or someone you know potentially having VKC, regardless of age, it's always best to consult with an ophthalmologist (eye doctor) for proper diagnosis and appropriate management.
5	How do I know if my child has vernal keratoconjunctivitis?	I cannot diagnose medical conditions, and it's crucial to consult with an ophthalmologist (eye doctor) if you suspect your child has vernal keratoconjunctivitis (VKC). They are qualified to perform a comprehensive examination, including: Asking about your child's medical history: This will involve questions about their symptoms, including their severity, duration, and any potential triggers like seasonal changes or exposure to allergens. Conducting a physical examination of the eyes: This will involve examining the eyelids, conjunctiva (the white part of the eye), cornea (the clear front part of the eye), and iris (the colored part of the eye) for signs of VKC, such as: Redness and swelling of the conjunctiva Bumpy, cobblestone-like appearance of the inner surface of the upper eyelid Thick, stringy discharge Light sensitivity (photophobia) In some cases, additional tests: These might include allergy testing to help identify potential triggers or special examinations to assess the health of the cornea. However, here are some common symptoms of VKC that you may observe in your child: Intense itching of the eyes, often described as worse in the mornings Excessive tearing and watery eyes Light sensitivity, making it uncomfortable to be in bright light Feeling like something is stuck in the eye (foreign body sensation) Blurred vision (may come and go) Redness of the whites of the eyes Increased blinking It's important to remember that these symptoms can also be indicative of other eye conditions. Therefore, seeking professional medical advice from an ophthalmologist is crucial for an accurate diagnosis and appropriate treatment plan tailored to your child's specific needs.
		While I cannot diagnose any medical condition, I can share some common symptoms of vernal keratoconjunctivitis (VKC) in children to help you be more informed when discussing your child's concerns with a healthcare professional: Common symptoms of VKC in children: Severe itching: This is often the most prominent and bothersome symptom. Redness: The whites of the eyes may appear red and inflamed. Tearing: Excessive watering of the eyes. Light sensitivity: The eyes may be sensitive to bright light. Blurry vision: Inflammation can cause temporary blurring of vision. Feeling of something in the eye: Children may describe a foreign object sensation in their eyes. Mucus discharge: Stringy or ropy discharge, particularly upon waking. "Morning misery": Difficulty opening the eyes and increased discomfort upon waking, often described as "morning misery" due to the accumulation of discharge overnight. Additional signs that may be observed by an ophthalmologist: Cobblestone-like bumps: Tiny bumps on the underside of the upper eyelid. Gelatinous nodules: Small, raised bumps on the white part of the eye near the cornea. Corneal involvement: In severe cases, the cornea may be affected, leading to complications like ulcers or scarring. It is crucial to consult an ophthalmologist (eye doctor) for proper diagnosis. They can: Perform a comprehensive eye examination, including looking for specific signs of VKC. Rule out other potential causes of your child's symptoms. Recommend the most appropriate treatment plan based on the severity and specific presentation of VKC in your child. Early diagnosis and management are crucial to prevent potential complications and improve your child's comfort and well-being.
		While I cannot diagnose your child's specific condition, I can share some general information about the symptoms of vernal keratoconjunctivitis (VKC) that might help you recognize the need to seek professional medical advice: Common symptoms of VKC in children include: Intense itching: This is often the most bothersome symptom and can be severe enough to disrupt sleep and daily activities. Redness (hyperemia) of the eyes. Watering (tearing) of the eyes. Light sensitivity (photophobia), making it uncomfortable to be in bright light. Feeling like something is in the eye (foreign body sensation). Thick, ropy discharge from the eyes, especially in the mornings. Blurred vision in some cases. Crusting of the eyelids upon waking. Additionally, in children with VKC, an eye doctor may observe: Cobblestone-like bumps on the inner surface of the upper eyelid (giant papillae). Other signs of inflammation on the surface of the eye. If your child experiences any of these symptoms, particularly intense itching and chronic eye discomfort, it's crucial to consult a qualified healthcare professional, preferably an ophthalmologist (eye doctor). They can perform a thorough eye examination, discuss your child's medical history, and provide an accurate diagnosis to determine the most appropriate treatment plan. Early diagnosis and management of VKC can help minimize discomfort, prevent complications, and improve your child's quality of life.
		I am unable to provide medical advice or diagnose any condition. It is crucial to consult a healthcare professional, preferably a pediatric ophthalmologist, to determine if your child has vernal keratoconjunctivitis (VKC) and receive proper treatment. Here are some general points to consider, but they are not a substitute for professional medical advice: Symptoms: While VKC symptoms can vary, common ones in children include: Intense itching of the eyes Sensitivity to light (photophobia) Excessive tearing Blurred vision Feeling like something is in the eye (foreign body sensation) Redness of the eye Thick, stringy discharge from the eye Difficulty opening eyes upon waking (especially in the morning) Diagnosis: A healthcare professional will conduct a comprehensive examination, including: Detailed medical history: Discussing symptoms, allergies, family history, and potential triggers. Eye examination: This may involve assessing the eyelids, cornea, conjunctiva, and tear film using specialized equipment. Treatment: There is no cure for VKC, but treatment aims to manage symptoms and prevent complications. This may involve: Artificial tears: To lubricate and soothe the eyes. Antihistamine eye drops: To reduce itching and inflammation. Corticosteroid eye drops: For severe cases, to reduce inflammation, but use with caution due to potential side effects. Cold compresses: To provide temporary relief from itching and inflammation. Allergy management: Identifying and avoiding triggers like pollen, dust mites, or mold, when possible. Remember, early diagnosis and management are crucial to prevent potential complications like corneal scarring, which can impair vision. Never hesitate to consult a healthcare professional if you have any concerns about your child's eye health.
		While I can't diagnose your child's condition, I can share some common symptoms of vernal keratoconjunctivitis (VKC) in children to help you identify potential signs and encourage you to seek professional medical advice: Common symptoms of VKC in children: Intense itching: This is often the most noticeable and bothersome symptom, sometimes leading to constant rubbing and irritation of the eyes. Redness: The whites of the eyes may appear red and inflamed. Tearing: Excessive tearing is a common response to the irritation caused by VKC. Light sensitivity (photophobia): Children with VKC may experience discomfort or pain in bright light. Thick, ropy discharge: The discharge can be stringy and may cause the eyelids to stick together, especially upon waking up. Blurry vision: In some cases, the inflammation and discharge can temporarily affect vision. Cobblestone-like bumps on the inner eyelids: These bumps, called papillae, are a characteristic sign of VKC. Seasonal pattern: VKC symptoms typically worsen during spring and summer months due to increased exposure to allergens like pollen, and improve during fall and winter. It's crucial to remember that these symptoms can also occur with other eye conditions. If you notice any of these signs in your child, especially if they are causing significant discomfort or affecting their daily life, it's important to consult a qualified healthcare professional, such as a pediatrician or ophthalmologist (eye doctor). They can perform a comprehensive eye examination, including looking for specific signs of VKC, and determine the most appropriate course of treatment for your child. Early diagnosis and management are crucial to prevent potential complications, such as corneal scarring, which can affect vision.
6	Why do you mostly get vernal keratoconjunctivitis in the spring and summer?	You are correct that vernal keratoconjunctivitis (VKC) typically worsens or starts during spring and summer. This seasonal pattern is primarily linked to the increased presence of allergens in the environment during these months. Here's a breakdown of the connection: Seasonal allergens: VKC is believed to be an allergic reaction, although the exact triggers remain unknown. Common allergens like pollen, dust mites, and mold spores are often more prevalent in the spring and summer months. Increased exposure: During these seasons, people tend to spend more time outdoors, leading to greater exposure to these allergens. This increased exposure can trigger or worsen the symptoms of VKC in individuals who are already susceptible. Here are some additional points to consider: Not everyone experiences seasonal variations: While the spring and summer months are common triggers, not everyone with VKC experiences significant seasonal variations. Some individuals may have symptoms year-round, while others may have flare-ups at different times of the year depending on their specific triggers. Other potential triggers: Beyond seasonal allergens, other factors like exposure to dust mites, pet dander, or smoke can also trigger or worsen VKC symptoms, regardless of the season. If you are concerned about your child's VKC, especially regarding seasonal variations or other potential triggers, it's advisable to consult with their ophthalmologist. They can provide personalized guidance and tailor a management plan considering your child's individual situation and potential triggers.
		Vernal keratoconjunctivitis (VKC) is indeed more prevalent in the spring and summer months, but it can occur any time of year. Here's why the seasonal connection exists: Seasonal Allergens: VKC is primarily an allergic reaction, and its seasonal association stems from the increased presence of allergens like pollen and dust mites in the environment during spring and summer. These allergens trigger an exaggerated immune response in individuals with VKC, leading to the characteristic inflammation and irritation in the eyes. However, it's important to consider: Not everyone with VKC experiences seasonality: Some individuals may experience year-round symptoms or have flare-ups triggered by specific allergens regardless of the season. Other factors can also contribute: Aside from allergens, factors like dry air and irritants like smoke or chlorine can exacerbate VKC symptoms at any time of year. Therefore, while the increased prevalence of VKC in spring and summer is due to seasonal allergens, it's important to understand that the condition is not strictly seasonal and can occur any time of year depending on individual triggers and sensitivities.
		You're correct that vernal keratoconjunctivitis (VKC) is more prevalent in the spring and summer months. This seasonal pattern is primarily linked to the increased presence of allergens in the environment during these times. Here's a breakdown of the reasons: Seasonal rise in allergens: Spring and summer often see a significant increase in pollen count, particularly from trees, grasses, and weeds. These airborne allergens can trigger allergic reactions in individuals with VKC, leading to the characteristic symptoms like itching, redness, and watering of the eyes. Increased outdoor activity: During spring and summer, people tend to spend more time outdoors, exposing themselves to these allergens at higher levels compared to colder months when they stay indoors more frequently. Other seasonal factors: While pollen is the primary culprit, other factors like increased humidity and dust mites during these months might also contribute to the worsening of VKC symptoms. It's important to note that not everyone with allergies will develop VKC, and while the condition is more common in spring and summer, it can occur year-round in some individuals who are exposed to specific triggers like dust mites or pet dander. If you suspect you or your child might have VKC, regardless of the season, consulting a healthcare professional is crucial for proper diagnosis and appropriate management to alleviate symptoms and prevent complications.
		Vernal keratoconjunctivitis (VKC) exhibits a seasonal pattern, primarily occurring and worsening during spring and summer months. This seasonal trend is mainly attributed to the increased presence of airborne allergens in the environment during these seasons. Here's a breakdown of the key factors: Seasonal allergies: VKC is primarily considered an allergic reaction in the eyes. Common allergens like pollen, dust mites, and mold spores are more prevalent in the spring and summer. Exposure to these allergens triggers an inflammatory response in the eyes of individuals with VKC, leading to the characteristic symptoms like itching, redness, and tearing. Environmental factors: Warm and dry climates are also associated with a higher prevalence of VKC. These conditions can exacerbate existing allergies and dry out the eyes, further contributing to irritation and discomfort. Therefore, the combined influence of increased allergens and environmental factors during spring and summer creates a higher risk for individuals with VKC to experience worsened symptoms. It's important to note that while seasonal variations are common, VKC can sometimes persist throughout the year or even develop for the first time in adults, regardless of the season. If you have any concerns about VKC or your child's eye health, especially during allergy seasons, consulting a healthcare professional is always recommended. They can help identify triggers, manage symptoms, and prevent potential complications.
		The seasonal nature of vernal keratoconjunctivitis (VKC) is primarily linked to the increased presence of airborne allergens like pollen, dust mites, and pet dander in the spring and summer months. These allergens trigger an excessive immune response in individuals susceptible to VKC, leading to the characteristic symptoms of inflammation, itching, and discharge. Here's a breakdown of the factors contributing to the seasonal pattern of VKC: Increased Allergen Levels: Spring and summer often witness higher pollen counts due to plant and tree pollination. Additionally, warmer temperatures and increased humidity can create favorable conditions for dust mites and mold, further contributing to the allergen burden in the environment. Outdoor Activities: During spring and summer, people tend to spend more time outdoors, engaging in activities like gardening, sports, and playing in parks. This increases their exposure to airborne allergens, exacerbating VKC symptoms. Dry Air: Spring and summer can also be times of lower humidity, particularly in certain climates. Dry air can irritate the eyes and potentially worsen VKC symptoms. While the seasonal pattern is a hallmark of VKC, it's important to note that: Some individuals may experience symptoms year-round, especially if they are exposed to allergens indoors or have other triggers. The severity of seasonal variation can differ between individuals, with some experiencing significant worsening during peak allergy seasons and others having milder fluctuations. If you or your child experiences VKC symptoms, it's crucial to consult an ophthalmologist (eye doctor) for proper diagnosis and personalized treatment. They can help manage symptoms, control the underlying allergic reaction, and minimize potential complications.
7	Can vernal keratoconjunctivitis be treated by a general ophthalmologist?	Yes, in most cases, vernal keratoconjunctivitis (VKC) can be effectively treated by a general ophthalmologist. General ophthalmologists are qualified to diagnose and manage a wide range of eye conditions, including VKC. They possess the necessary expertise to: Perform a comprehensive eye examination: This includes reviewing your child's medical history, examining their eyes for signs of VKC, and potentially conducting additional tests if needed. Diagnose VKC: Based on the examination findings and your child's symptoms, a general ophthalmologist can accurately diagnose VKC. Develop a treatment plan: They can create a personalized treatment plan tailored to the severity of your child's VKC. This plan typically involves: Non-steroidal medications: Medications like antihistamines and mast cell stabilizers can help reduce itching, inflammation, and other symptoms. Cold compresses: Applying cold compresses to the eyes can help alleviate itching and swelling. Allergen avoidance: Identifying and avoiding potential allergens can help prevent flare-ups. In some cases, topical corticosteroids: For severe cases, a general ophthalmologist may prescribe short-term topical corticosteroids to manage inflammation. However, due to potential side effects, they will be cautious and monitor your child closely while using them. Referral to a specialist: While general ophthalmologists are well-equipped to manage most cases of VKC, there might be situations where a referral to a specialist is necessary. This could include: Severe or complex cases: If your child has a very severe or complex case of VKC that is not responding well to standard treatment, a referral to an ophthalmologist specializing in corneal diseases or pediatric ophthalmology might be recommended. Suspected complications: In rare cases, VKC can lead to complications like corneal scarring. If the ophthalmologist suspects such complications, they might refer your child to a specialist for further evaluation and management. Overall, a general ophthalmologist is an excellent starting point for diagnosing and treating VKC. They can effectively manage most cases and determine if a referral to a specialist is necessary in your child's specific situation.
		Yes, in most cases, vernal keratoconjunctivitis (VKC) can be effectively treated by a general ophthalmologist (eye doctor). Ophthalmologists are qualified to diagnose and manage a wide range of eye conditions, including VKC. Here's why a general ophthalmologist is often well-equipped for VKC treatment: Expertise in eye diseases: Ophthalmologists have extensive training and experience in diagnosing and treating various eye conditions, including allergic eye diseases like VKC. Diagnosis and treatment: They can perform a comprehensive eye exam to diagnose VKC, differentiate it from other conditions, and recommend the most appropriate treatment plan based on the severity and specific presentation of the case. Treatment options: General ophthalmologists have access to a wide range of treatment options for VKC, including: Non-steroidal medications: Artificial tears, lubricants, and antihistamine eye drops. Mast cell stabilizers: To prevent the release of inflammatory substances. Steroid eye drops: In severe cases, but used cautiously due to potential side effects. Immunomodulators: For more severe or persistent cases. Referral to a specialist: While general ophthalmologists can effectively manage most VKC cases, there might be situations where a referral to a specialist is recommended. Here are some potential scenarios: Complex or severe cases: If the VKC is particularly severe, involves complications like corneal involvement, or doesn't respond well to standard treatments, a referral to an ophthalmologist specializing in corneal diseases or pediatric ophthalmology might be considered. Multidisciplinary approach: In some cases, a collaborative approach involving an ophthalmologist and other specialists like an allergist or immunologist might be necessary for optimal management, particularly if there are underlying allergies or complex immune system factors contributing to the VKC. Overall, a general ophthalmologist is a good first point of contact for diagnosis and treatment of VKC. They can assess the situation and determine if a referral to a specialist is necessary based on the individual case.
		Yes, vernal keratoconjunctivitis (VKC) can typically be effectively treated by a general ophthalmologist. Here's why: General ophthalmologists are qualified eye doctors with extensive training in diagnosing and treating a wide range of eye conditions, including allergic conditions like VKC. They possess the expertise to perform a comprehensive eye examination, discuss your medical history, and identify the signs and symptoms characteristic of VKC. General ophthalmologists have access to a broad spectrum of medications commonly used to manage VKC, including: Artificial tears for lubrication Antihistamine eye drops or oral medications to relieve itching Mast cell stabilizers to prevent the release of inflammatory substances Corticosteroid eye drops in severe cases (used cautiously under their supervision) While some complex eye conditions might require referral to a specialist, VKC typically falls within the scope of a general ophthalmologist's expertise. Here are some situations where a referral to a specialist might be considered: Unusually severe or persistent VKC: If the general ophthalmologist struggles to achieve adequate control of symptoms despite appropriate treatment. Suspected complications: In rare cases, VKC can lead to corneal complications. A specialist might be needed if such complications arise. Patient preference: If you prefer to see a pediatric ophthalmologist specializing in childhood eye conditions, you can discuss this with your general ophthalmologist and request a referral. Ultimately, the decision of who to consult depends on your specific situation and the severity of your child's VKC. However, in most cases, a general ophthalmologist can effectively manage and treat VKC.
		In many cases, yes, a general ophthalmologist can effectively treat vernal keratoconjunctivitis (VKC), especially for mild to moderate cases. General ophthalmologists are qualified eye doctors with expertise in diagnosing and treating a wide range of eye conditions, including VKC. Here's a breakdown of when a general ophthalmologist can manage VKC: Diagnosis and initial treatment: A general ophthalmologist can diagnose VKC through a comprehensive eye examination and medical history review. They can then initiate appropriate treatment plans, typically involving: Artificial tears for lubrication Antihistamine eye drops to reduce itching and inflammation Cold compresses for temporary relief Management of mild to moderate VKC: General ophthalmologists can effectively manage most cases of VKC that fall under the mild to moderate category. This includes monitoring the condition, adjusting treatment plans as needed, and offering guidance on allergy management. However, there are situations where a referral to a specialist might be recommended: Severe VKC: In cases of severe VKC that are unresponsive to initial treatment or involve complications, a referral to a corneal specialist or pediatric ophthalmologist (for children) might be necessary. These specialists have additional expertise in managing complex eye conditions and can provide more advanced treatment options like: Topical corticosteroids: Used cautiously due to potential side effects, but can be helpful in severe cases. Immunomodulators: Medications like cyclosporine A, used in severe cases to suppress the underlying immune response. Ultimately, the decision of whether a referral is needed will depend on the severity of your child's VKC and their response to initial treatment. Your general ophthalmologist will be able to advise you on the best course of action.
		In many cases, yes, a general ophthalmologist can effectively treat vernal keratoconjunctivitis (VKC). Here's a breakdown of how a general ophthalmologist can address VKC: Diagnosis and Management: General ophthalmologists are qualified to diagnose VKC through a comprehensive eye examination, including looking for specific signs like cobblestone-like bumps on the inner eyelids (papillae). They can prescribe first-line medications typically used for VKC, such as: Artificial tears: To soothe and lubricate the eyes. Mast cell stabilizers: To prevent the release of inflammatory chemicals from mast cells in the eye. Topical antihistamines: To reduce itching and allergic response. They can monitor the effectiveness of treatment and adjust medication regimens as needed. Referral to a Specialist: In some cases, a general ophthalmologist might recommend a referral to a specialist, such as an oculoplastic surgeon or a pediatric ophthalmologist. This may be necessary for: Severe VKC that doesn't respond well to initial treatment. Cases with potential complications like corneal scarring. Children with VKC, particularly if the diagnosis or treatment plan requires a more specialized approach. Benefits of Consulting a General Ophthalmologist: Accessibility: General ophthalmologists are often more readily available than specialists. Comprehensive Eye Care: They can address other potential eye problems during the same visit. Treatment Initiation: They can promptly initiate treatment to manage symptoms and prevent complications. Overall, a general ophthalmologist can be a valuable first point of contact for diagnosing and managing VKC. However, if the case is severe or requires specialized expertise, they can make appropriate referrals to ensure optimal care.
8	What is the prognosis for vernal keratoconjunctivitis?	The prognosis for vernal keratoconjunctivitis (VKC) is generally good. Here's a breakdown of what you can expect: Favorable outcome: In most cases, VKC is a self-limiting disease, meaning the symptoms often improve or resolve spontaneously around puberty, typically between the ages of 12 and 16. Management of symptoms: Even though VKC often resolves on its own, treatment plays a crucial role in managing symptoms and improving your child's quality of life during flare-ups. Following a doctor's prescribed treatment plan can significantly reduce discomfort and prevent complications. Potential for persistence: In some cases, VKC can persist beyond puberty, affecting up to 12% of patients. This is more common in individuals with severe cases or those with a strong family history of allergies. Here are some additional factors to consider: Early diagnosis and treatment: Seeking medical attention early and adhering to the treatment plan can help prevent complications and improve your child's overall prognosis. Complications: While rare, VKC can lead to complications like corneal scarring if left untreated or not managed effectively. This is why following a doctor's recommendations is crucial. Individual variations: The course of VKC can vary from person to person. Some children may experience mild, infrequent symptoms, while others may have more severe, persistent cases. Overall, the prognosis for VKC is positive. With proper management, most children experience significant improvement or complete resolution of symptoms by puberty. Consulting with an ophthalmologist is crucial to ensure a proper diagnosis, develop an effective treatment plan, and monitor your child's progress to minimize the risk of complications.
		The prognosis for vernal keratoconjunctivitis (VKC) is generally good, but it depends on several factors: Favorable outlook: Self-limiting: In many children, VKC is a self-limiting condition that often improves or resolves spontaneously around puberty. Effective treatment: Early diagnosis and proper treatment with medications like antihistamines, mast cell stabilizers, or corticosteroids (used cautiously) can effectively manage symptoms and prevent complications. Minimal visual impact: With proper management, VKC typically doesn't cause permanent vision loss. Potential concerns: Chronic inflammation: In some cases, VKC can become chronic, leading to persistent inflammation and discomfort. Corneal complications: Uncontrolled inflammation can potentially lead to complications like corneal ulcers or scarring, which can affect vision. Adult VKC: Although less common, VKC can persist or develop for the first time in adults, requiring ongoing management. Here's a breakdown of potential outcomes: Most cases: VKC resolves with age or puberty with minimal to no lasting impact on vision with proper treatment. Some cases: May develop into chronic VKC requiring ongoing management. Rare cases: Can lead to corneal complications impacting vision if not managed effectively. Importance of consulting an ophthalmologist: Early diagnosis and proper treatment plan from an ophthalmologist are crucial to: Improve your child's comfort and quality of life. Minimize the risk of complications and potential vision problems. They can monitor your child's condition and adjust treatment as needed to ensure the best possible outcome.
		The prognosis for vernal keratoconjunctivitis (VKC) is generally good. Here's a breakdown of what you can expect: Self-limiting: In most cases, VKC is a self-limiting disease, meaning the symptoms improve over time and often resolve by puberty. Favorable outcome: With proper management and avoidance of triggers, many children experience significant relief and a return to normal vision. Chronic cases: While less common, VKC can persist into adulthood in some cases (around 12% of patients). There's also a rarer form that can develop for the first time in adults. Here's a look at some factors influencing the prognosis: Early diagnosis and treatment: Early intervention with appropriate management strategies can significantly improve the prognosis and prevent complications. Severity of symptoms: The severity of symptoms and the frequency of flare-ups can influence the long-term outcome. Compliance with treatment: Following the treatment plan prescribed by the ophthalmologist is crucial for optimal outcomes. Potential complications: Corneal damage: In severe or uncontrolled cases, chronic inflammation can lead to damage to the cornea, the clear dome at the front of the eye. This can potentially affect vision. Vision impairment: Scarring of the cornea is a rare complication that can cause permanent vision impairment. Cataracts or glaucoma: While uncommon, long-term use of certain medications like corticosteroids for severe VKC may increase the risk of cataracts or glaucoma. Here are some tips to improve the prognosis: Schedule regular eye checkups: Regular eye examinations by your ophthalmologist are essential to monitor the condition, adjust treatment as needed, and detect any potential complications early on. Identify and avoid triggers: If possible, try to identify and avoid allergens or irritants that might trigger VKC flare-ups. Follow the treatment plan: It's crucial to adhere to the treatment plan recommended by your ophthalmologist, which may involve medications like artificial tears, antihistamines, mast cell stabilizers, or corticosteroids in severe cases. Practice good eye hygiene: Maintaining proper eye hygiene practices like avoiding eye rubbing and using cool compresses can help soothe symptoms and prevent infections. If you have any concerns about your child's VKC or its prognosis, discussing them with your ophthalmologist is important. They can provide personalized guidance based on your child's specific situation.
		The prognosis for vernal keratoconjunctivitis (VKC) is generally positive, particularly with early diagnosis and proper management. Here's a breakdown of what you can expect: Self-limiting in children: In most children, VKC is considered self-limiting, meaning the symptoms often improve or resolve spontaneously around puberty. This is because the underlying immune response that triggers VKC tends to mature by puberty. Favorable outcome with treatment: Even before puberty, following a prescribed treatment plan from an ophthalmologist can significantly alleviate symptoms and prevent complications. This allows children to experience better comfort and maintain good vision. Potential for persistence: In some cases, VKC symptoms may persist beyond childhood into adulthood. This is more common in individuals with a strong allergic predisposition. However, even in these cases, proper management strategies can still effectively control symptoms and prevent complications. Risk of complications: Uncontrolled VKC, especially when severe, can lead to complications like corneal scarring which can potentially affect vision. This highlights the importance of seeking medical attention for proper diagnosis and management. Here are some additional points to consider: Individual variability: The prognosis for VKC can vary depending on the individual's severity, response to treatment, and allergic tendencies. Long-term management: For adults with persistent VKC, ongoing management with an ophthalmologist might be necessary to control symptoms and prevent complications. If you have any concerns about your child's VKC or its prognosis, consulting a healthcare professional is always recommended. They can provide personalized guidance based on your child's specific situation.
		The prognosis for vernal keratoconjunctivitis (VKC) is generally good, particularly with early diagnosis and proper treatment. Here's a breakdown of what you can expect: Favorable Outcome: Self-limiting: In many cases, especially in children, VKC is considered self-limiting. This means symptoms often improve or resolve spontaneously around puberty, with some studies suggesting spontaneous resolution in up to 80% of cases. Effective Treatment: Medication and other management strategies can effectively control symptoms and improve quality of life. Minimal Long-Term Effects: With proper management, most individuals with VKC do not experience long-term vision problems. Potential Concerns: Persistent Symptoms: While uncommon, VKC symptoms can persist beyond puberty in some adults (around 12% of cases). Complications: Uncontrolled VKC or improper treatment with certain medications (like corticosteroids) can lead to complications like corneal ulcers, scarring, and vision impairment. These complications are rare but highlight the importance of seeking professional medical advice. Importance of Management: Even though VKC often resolves on its own, proper management is crucial for several reasons: Symptom Relief: Effective treatment can significantly improve comfort and reduce bothersome symptoms like itching, tearing, and light sensitivity. Prevention of Complications: Early intervention can help prevent complications like corneal damage that could potentially affect vision. Improved Quality of Life: Managing VKC can significantly enhance quality of life by allowing individuals to participate in normal activities without discomfort. Overall, the prognosis for VKC is positive. With proper diagnosis and management by a qualified healthcare professional, most individuals can achieve good control of symptoms and prevent potential complications.
9	When will my kid’s vernal keratoconjunctivitis go away?	Unfortunately, I can't predict exactly when your child's vernal keratoconjunctivitis (VKC) will go away. The reason is twofold: Individual variations: VKC affects children differently. Some experience mild, infrequent symptoms that resolve quickly, while others have more severe, persistent cases that may last for years. Dependence on multiple factors: The course of VKC depends on factors like: Severity of the condition: Milder cases tend to resolve sooner than severe ones. Management strategies: Following a doctor-recommended treatment plan effectively can significantly reduce symptoms and potentially shorten their duration. Exposure to triggers: Avoiding allergens that trigger flare-ups can help prevent worsening symptoms and potentially shorten their course. However, here's some general information that might be helpful: Self-limiting disease: In most cases, VKC is a self-limiting disease, meaning the symptoms typically improve or resolve spontaneously around puberty, usually between the ages of 12 and 16. Recommendations: Consult an ophthalmologist: They can assess your child's specific situation, develop a personalized treatment plan, and offer the most accurate prognosis based on your child's individual case. Management is key: Even though VKC often resolves on its own, following the prescribed treatment plan can significantly improve your child's comfort and potentially shorten the duration of symptoms. Focus on prevention: Identifying and avoiding triggers like allergens can help prevent flare-ups and potentially shorten the overall course of the condition. By consulting an ophthalmologist and following their recommendations, you can create the best environment for your child's VKC to improve and potentially resolve as quickly as possible.
		Unfortunately, I can't predict exactly when your child's vernal keratoconjunctivitis (VKC) will go away. The course of VKC varies depending on individual factors, but here's some general information that might be helpful: Favorable Outlook: Self-limiting: In many children, VKC is a self-limiting condition that often improves or resolves spontaneously around puberty. This is the most likely scenario. Factors influencing resolution: Age: VKC typically improves with age, with most cases resolving by puberty. Severity: Milder cases tend to resolve faster than severe cases. Treatment: Early diagnosis and proper treatment can accelerate improvement and prevent complications. Recommendations: Consult an ophthalmologist: They can assess your child's specific case and provide a more informed estimate based on the severity and presentation of VKC. Management plan: Following a prescribed treatment plan from the ophthalmologist can help manage symptoms and potentially expedite improvement. Here are some additional resources you may find helpful: American Academy of Ophthalmology: https://www.aao.org/education/disease-review/vernal-keratoconjunctivitis-5 National Eye Institute: https://www.nei.nih.gov/ Remember, while VKC can be bothersome, it's generally a manageable condition, and early intervention with an ophthalmologist is key to optimizing your child's comfort and preventing complications.
		I can't give a specific timeframe for when your child's vernal keratoconjunctivitis (VKC) will go away because it varies depending on individual cases. However, here's some general information that might be helpful: Self-limiting condition: In most cases, VKC is a self-limiting disease, meaning it improves with time and often resolves by puberty. Favorable outcome: With proper management and avoidance of triggers, many children experience significant relief and a return to normal vision within a few weeks or months of starting treatment. Here are some factors that can influence how long it takes for your child's VKC to improve: Severity of symptoms: More severe cases with frequent flare-ups might take longer to manage and control symptoms compared to milder cases. Early diagnosis and treatment: Early intervention with appropriate medications and management strategies can significantly improve the speed of recovery. This is why consulting a healthcare professional is crucial. Compliance with treatment: Following the treatment plan prescribed by the ophthalmologist is essential for optimal outcomes. Sticking to the recommended schedule and dosage of medications and eye hygiene practices will contribute to faster improvement. Recommendations: Consult an ophthalmologist: They can assess your child's specific condition, recommend the most appropriate treatment plan, and provide guidance on managing triggers and preventing complications. They can also give you a more precise idea of what to expect in your child's case. Focus on management: While there isn't a cure for VKC, effective management strategies can significantly improve your child's quality of life and minimize discomfort. Remember, I am not a medical professional and cannot provide diagnosis or treatment advice. However, I hope this information helps!
		Unfortunately, I can't predict exactly when your child's vernal keratoconjunctivitis (VKC) will go away. Here's what I can tell you based on what we know about VKC: Generally self-limiting in children: In most children, VKC tends to improve or resolve around puberty because the underlying immune response that triggers VKC matures during this time. Variability exists: The course of VKC can vary from child to child. Some children experience symptoms for just a few seasons, while others may have them for several years. Here's what might help get a better idea: Consulting your child's ophthalmologist: They can assess your child's specific case and provide a more informed prognosis based on the severity and their response to treatment. Considering the age: If your child is approaching puberty, there's a higher chance the VKC might improve on its own. Remember, even if VKC doesn't completely disappear on its own, following a prescribed treatment plan from an ophthalmologist can significantly alleviate symptoms and prevent complications. This allows your child to experience better comfort and maintain good vision.
		I can't give a specific timeframe for when your child's vernal keratoconjunctivitis (VKC) will go away. Unfortunately, predicting the exact course is difficult. However, here's some general information that might be helpful: Self-Limiting: In many children with VKC, especially those diagnosed before puberty, the condition is self-limiting. This means it often improves or even resolves entirely around puberty. Studies suggest spontaneous resolution in up to 80% of cases. Variable Course: The course of VKC can vary significantly between children. Some may experience a rapid improvement, while others might have symptoms that linger for a while. Importance of Diagnosis and Management: Even though VKC often resolves on its own, consulting an ophthalmologist is crucial. They can: Confirm the diagnosis. Recommend the most appropriate treatment plan to manage symptoms and prevent complications. Monitor your child's progress and adjust treatment as needed. Recommendations: Consult an ophthalmologist: A qualified eye doctor can provide the most accurate assessment and guidance for your child's specific case. Discuss Prognosis: During the appointment, ask the ophthalmologist about the expected course of VKC for your child and any potential factors that might influence it. Follow Treatment Plan: Adhering to the prescribed treatment plan is essential to manage your child's symptoms and prevent complications. By seeking professional medical advice and following the recommended treatment plan, you can help your child manage VKC effectively and improve their quality of life.
10	Can you get vernal keratoconjunctivitis as an adult?	Yes, it is possible to develop vernal keratoconjunctivitis (VKC) as an adult, although it is much less common than in children. Here's a breakdown of what you need to know: Primarily a childhood condition: VKC typically affects young boys between the ages of 5 and 12. Adult VKC: When VKC persists beyond puberty, it's referred to as adult VKC. This form is considered rare, affecting only around 1% of all allergic conjunctivitis cases and 2.7% of total VKC patients. Two variants: Adult VKC can present in two forms: Early-onset: This occurs when childhood VKC fails to resolve around puberty and continues into adulthood. Late-onset: This is less common and refers to VKC developing for the first time in an adult with no childhood history of the condition. Reasons for rarity in adults: Unknown: The exact reasons for the rarity of adult VKC are not fully understood. Possible explanations: Some theories suggest that adults may develop a different immune response to allergens compared to children, potentially making them less susceptible to VKC. Additionally, lifestyle changes in adults, like spending less time outdoors compared to children, might reduce exposure to certain triggers. If you suspect adult VKC: See an ophthalmologist: They can perform a comprehensive eye examination to diagnose adult VKC and differentiate it from other conditions with similar symptoms. Treatment: The treatment approach for adult VKC is generally similar to childhood VKC, focusing on managing symptoms and preventing complications. This may involve medications like antihistamines, mast cell stabilizers, and corticosteroids (used cautiously due to potential side effects) alongside cold compresses and allergen avoidance strategies. Remember: Adult VKC is uncommon but possible. Early diagnosis and management are crucial for preventing complications. Consulting an ophthalmologist is essential for proper diagnosis and treatment.
		Yes, it is possible to get vernal keratoconjunctivitis (VKC) as an adult, although it is much less common than in children. Here's a breakdown: Predominantly affects children: VKC primarily affects young boys between 1 and 22 years old, with a peak around 6-7 years old. It often resolves or becomes less severe after puberty. Adult VKC: While less frequent, VKC can persist beyond childhood or even develop for the first time in adults. This is called adult-onset VKC. There are two variants of adult VKC: Early-onset: Childhood VKC persisting into adulthood. Late-onset: VKC arising de novo (for the first time) in adults. Possible reasons for adult VKC: The exact reasons for adult VKC are not fully understood, but some theories suggest: Atopic predisposition: Individuals with allergies or atopic conditions like eczema or asthma might be more susceptible throughout their lives. Environmental triggers: Ongoing exposure to allergens or irritants could contribute to VKC development in adults. If you suspect VKC as an adult: Consult an ophthalmologist (eye doctor) for proper diagnosis. VKC can mimic other eye conditions in adults. They can develop a treatment plan tailored to your specific needs, potentially including: Non-steroidal medications: Artificial tears, lubricants, and antihistamine eye drops. Mast cell stabilizers: To prevent the release of inflammatory substances. Steroid eye drops: In severe cases, but used cautiously due to potential side effects. Immunomodulators: For more severe or persistent cases. Importance of early diagnosis and management: Early intervention by an ophthalmologist is crucial to manage symptoms, prevent complications like corneal damage, and improve your quality of life.
		Yes, while vernal keratoconjunctivitis (VKC) is primarily a childhood disease, it can occur in adults, although it's less common. Here's a breakdown of how VKC presents in adults: Childhood persistence: Up to 12% of childhood VKC cases may persist into adulthood. Adult-onset VKC: There's also a rarer form of VKC that can develop for the first time in adults with no prior childhood history (de novo). Adult VKC can differ from childhood VKC in a few ways: Less self-limiting: Adult VKC often isn't self-limiting and may require ongoing management to control symptoms. Increased risk of complications: Chronic inflammation in adult VKC can lead to a higher risk of complications like corneal scarring compared to childhood VKC. If you suspect you might have adult VKC, here are some signs to watch out for: Itching: This is often the most bothersome symptom. Redness (hyperemia) of the eyes Watering (tearing) of the eyes Light sensitivity (photophobia) Feeling like something is in the eye (foreign body sensation) Thick, ropy discharge from the eyes It's crucial to consult an ophthalmologist (eye doctor) if you experience these symptoms. They can perform a comprehensive eye examination, discuss your medical history, and provide an accurate diagnosis to determine the most appropriate treatment plan for adult VKC.
		Yes, it is possible to get vernal keratoconjunctivitis (VKC) as an adult, although it is much less common than in children. Here's a breakdown of what you need to know: Predominantly affects children: VKC typically affects young boys between the ages of 5 and 12. Adult VKC exists: There are cases where VKC persists beyond childhood (adult-onset VKC) or develops for the first time in adults. This is estimated to occur in around 12% of VKC cases. Types of adult VKC: Early-onset: This refers to childhood VKC that persists beyond puberty. Late-onset: This describes VKC that develops for the first time in adulthood. Adult VKC characteristics: Less self-limiting: Unlike childhood VKC, adult VKC often doesn't resolve spontaneously and can be more persistent. Increased risk of complications: Uncontrolled adult VKC can lead to a higher risk of complications like corneal scarring compared to childhood VKC. If you suspect adult VKC, it's crucial to consult an ophthalmologist for proper diagnosis and management. Treatment for adult VKC may involve: Similar strategies to childhood VKC: This includes artificial tears, antihistamine eye drops, and cold compresses. Potentially stronger medications: In some cases, ophthalmologists might prescribe stronger medications like topical corticosteroids (used cautiously due to side effects) or immunomodulators for severe cases. Remember: Early diagnosis and proper management are essential to control symptoms, prevent complications, and maintain good vision. Don't hesitate to seek professional help if you experience any symptoms suggestive of VKC, regardless of your age.
		Yes, it is possible to get vernal keratoconjunctivitis (VKC) as an adult, although it is much less common than in children. Here's a breakdown of what you need to know: Predominantly Childhood Condition: VKC typically affects young boys between the ages of 5 and 12. Studies suggest that around 88% of VKC cases occur in children. Adult Onset VKC: Adult-onset VKC is a distinct entity and represents only a small percentage of all VKC cases, estimated to be around 1% of all allergic conjunctivitis and 2.7% of total VKC patients. Two Variants: Adult VKC can occur in two forms: Early Onset: This refers to childhood VKC persisting beyond puberty. Late Onset: This describes VKC arising for the first time in adults with no childhood history. Reasons for Less Prevalence in Adults: Possible Outgrowing: The exact reasons are not fully understood, but some theories suggest that children may outgrow their sensitivity to allergens that trigger VKC. Changes in Immune System: The immune system matures as we age, potentially making adults less susceptible to the specific allergic response that causes VKC. If you suspect you have VKC as an adult, it's important to consult an ophthalmologist (eye doctor) for proper diagnosis. Adult VKC can differ from the childhood form in some aspects: Less Self-Limiting: Unlike childhood VKC, adult VKC often doesn't resolve spontaneously and may require ongoing management. Increased Risk of Complications: Uncontrolled adult VKC can have a higher risk of complications like corneal scarring compared to childhood VKC. Early diagnosis and treatment are crucial for managing VKC in adults and preventing potential complications. The ophthalmologist can develop a personalized treatment plan to address your specific needs and symptoms.
11	Is vernal keratoconjunctivitis inherited?	The link between genetics and vernal keratoconjunctivitis (VKC) is a bit complex. While there's no definitive evidence for VKC being a directly inherited condition, there are suggestive factors that point towards a possible genetic influence: Family history: Studies have shown that children with a family history of allergies, such as asthma, eczema, or allergic rhinitis, are at a higher risk of developing VKC. This suggests a potential genetic predisposition to allergic reactions that might play a role in VKC development. Geographical patterns: VKC is more prevalent in certain geographical regions, suggesting a possible role of genetic makeup in combination with environmental factors. However, here's what we currently understand about the limitations of the inheritance link: No identified genes: Researchers haven't identified specific genes directly responsible for causing VKC. Complex interaction: VKC is likely caused by a complex interplay of genetic and environmental factors. While there might be a genetic predisposition, it's not the sole determinant. Overall: There's evidence suggesting a possible genetic influence on VKC susceptibility, but it's not a straightforward inheritance pattern. Family history of allergies increases the risk, but it doesn't guarantee that someone will develop VKC. If you're concerned about your child's risk of VKC due to family history, consulting with an ophthalmologist can be helpful. They can discuss your child's specific situation and offer personalized advice.
		The inheritance of vernal keratoconjunctivitis (VKC) is a complex issue and there's no simple yes or no answer. Here's a breakdown of what we currently know: Genetic predisposition: Research suggests a potential genetic predisposition for VKC. This means that having a family history of allergies or atopic conditions like eczema or asthma can increase your risk of developing VKC. However, specific genes linked to VKC haven't been definitively identified. Increased prevalence in families: Studies show a higher prevalence of VKC in families with a history of atopy, suggesting a possible hereditary component. Not a simple Mendelian inheritance: VKC likely doesn't follow a simple Mendelian inheritance pattern (where a single gene determines the trait). It's likely a polygenic condition influenced by multiple genes and environmental factors. Current understanding: The exact genetic link to VKC remains under investigation. While there's evidence suggesting a hereditary component, the specific genes involved and the inheritance pattern are not fully understood. What this means: Having a family history of allergies increases your risk of VKC, but it doesn't guarantee you'll develop it. Environmental factors like exposure to allergens also play a significant role. Future research: Ongoing research aims to identify specific genes associated with VKC susceptibility. If you're concerned about your risk of VKC: Talk to your doctor, especially if you have a family history of allergies or atopic conditions. They can discuss your individual risk factors and recommend appropriate preventive measures. Remember, even with a family history, VKC is a manageable condition. Early diagnosis and proper treatment from an ophthalmologist are key to controlling symptoms and preventing complications.
		The inheritance of vernal keratoconjunctivitis (VKC) is a complex issue and there's no simple yes or no answer. Here's a breakdown of what we currently know: Genetic predisposition: A family history of allergies, including atopic dermatitis (eczema) and asthma, significantly increases the risk of developing VKC. This suggests a genetic susceptibility might play a role. No identified genes: Researchers haven't identified specific genes directly causing VKC. The genetic influence is likely due to a combination of multiple genes, making it more challenging to pinpoint a clear inheritance pattern. Here's an analogy to understand the concept better: Imagine VKC susceptibility like needing a key to open a door. Genetics might provide you with the key blank, but environmental factors like exposure to allergens would be required to "cut" the key and make it functional. In summary: A family history of allergies increases the risk of VKC, suggesting a genetic influence. Specific genes linked to VKC haven't been identified. The inheritance pattern is likely complex and involves multiple genes interacting with environmental factors. If you have concerns about your family history and VKC risk, consulting a healthcare professional or a genetic counselor can be helpful. They can provide more specific guidance based on your individual situation.
		The inheritance of vernal keratoconjunctivitis (VKC) is a subject of ongoing research, and there's no definitive answer yet. Here's what we know: Genetic predisposition suspected: There's evidence suggesting a genetic susceptibility to VKC. This means having a family history of allergies or atopic conditions like eczema or asthma can increase your child's risk of developing VKC. No identified genes: Despite the suspected genetic link, researchers haven't identified specific genes directly responsible for causing VKC. Complex interplay: The development of VKC likely involves a complex interplay between genetic predisposition and environmental factors like exposure to allergens. Here's an analogy: Imagine VKC susceptibility like needing a key and a lock to open a door. Genetics might provide the key (predisposition), but environmental triggers (allergens) act like the lock. Without both, the door (VKC) wouldn't open. Key points to remember: A family history of allergies increases the risk of VKC, but it's not a guaranteed inheritance. The exact genes involved in VKC remain unknown. Environmental factors play a significant role in triggering VKC in individuals with a genetic susceptibility. If you're concerned about your child's risk of VKC due to family history, consulting a healthcare professional can be helpful. They can discuss your child's specific situation and offer guidance on managing allergies and reducing exposure to potential triggers.
		The inheritance of vernal keratoconjunctivitis (VKC) is a complex issue with no simple answer. Here's what we know currently: Likely Plays a Role: While the exact cause of VKC is unknown, a genetic component is suspected to play a role. Family History: Individuals with a family history of allergies, such as asthma, eczema, or allergic rhinitis, are more at risk of developing VKC. This suggests a potential genetic link. No Identified Genes: Despite the association with allergies, researchers haven't identified specific genes directly causing VKC. Studies investigating potential genetic associations with VKC have yielded inconclusive results. Here's a more detailed breakdown of the current understanding: Complex Interaction: VKC is likely caused by a combination of genetic and environmental factors. Genetic Predisposition: Certain genes might make individuals more susceptible to developing VKC if exposed to triggering allergens. Immune System: These genes might influence the way the immune system reacts to allergens, leading to the characteristic inflammatory response seen in VKC. More Research Needed: Scientists are still actively researching the specific genes and how they might influence the development of VKC. Further studies are needed to fully understand the role of genetics in VKC. In conclusion, while a genetic predisposition is likely involved in VKC, it's not a straightforward inheritance pattern. Family history of allergies increases the risk, but specific genes haven't been pinpointed yet. If you have concerns about your family history and VKC risk, consulting a healthcare professional is recommended for personalized advice.
12	Can you go blind from vernal keratoconjunctivitis?	Vernal keratoconjunctivitis (VKC) itself is unlikely to directly cause blindness. However, in rare cases, if left untreated or not managed effectively, VKC can lead to complications that can potentially impair vision or even lead to blindness. Here's a breakdown of the risks: Generally not blinding: The good news is that VKC typically doesn't cause blindness. With proper diagnosis and management, most people with VKC experience significant improvement or complete resolution of symptoms. Potential complications: In some severe or untreated cases, VKC can lead to complications like: Corneal scarring: This is the most concerning complication. Chronic inflammation caused by VKC can damage the cornea, the clear front part of the eye, leading to scarring. Significant scarring can affect vision and in rare cases, can even cause blindness. Steroid-induced cataracts: If topical corticosteroids are used excessively or for extended periods to treat VKC symptoms, they can increase the risk of developing cataracts, which can cloud the lens of the eye and blur vision. Importance of management: Early diagnosis and treatment: Seeking medical attention promptly and adhering to the prescribed treatment plan can significantly reduce the risk of complications like corneal scarring. Ophthalmologist's role: An ophthalmologist can monitor your child's condition and adjust the treatment plan as needed to minimize the risk of complications. Overall: While blindness is not a common outcome of VKC, proper management is crucial to prevent complications that could potentially affect vision. Early diagnosis, following a doctor's recommendations, and regular checkups with an ophthalmologist are essential for maintaining good eye health and preventing vision problems. If you have any concerns about your child's VKC or the risk of complications, consulting with their ophthalmologist is the best course of action. They can address your specific questions and provide personalized guidance based on your child's individual situation.
		Vernal keratoconjunctivitis (VKC) itself typically does not lead to blindness. However, in rare cases, if left untreated or improperly managed, it can cause complications that can potentially impair vision. Here's a breakdown: Vision loss unlikely: In most cases, VKC is a chronic allergic condition that primarily causes discomfort and irritation, not permanent vision loss. Early diagnosis and proper treatment with medications like antihistamines, mast cell stabilizers, or corticosteroids (used cautiously) can effectively manage symptoms and prevent complications. Potential complications: Uncontrolled chronic inflammation from VKC can lead to: Corneal ulcers: Open sores on the cornea, the clear outer dome of the eye. Corneal scarring: Healing of corneal ulcers can leave scar tissue, which can distort the shape of the cornea and affect vision. Vision impairment: Severe corneal ulcers or scarring can cause: Blurred vision Light sensitivity Decreased vision Importance of management: Regular eye checkups with an ophthalmologist are crucial to monitor your child's VKC and address any potential complications promptly. Early intervention can prevent vision-threatening complications. Blindness is rare: While severe corneal scarring can significantly impact vision, complete blindness from VKC is very uncommon. Remember: With proper management, most people with VKC experience minimal to no lasting impact on vision. Early diagnosis and adherence to a treatment plan from an ophthalmologist are key to preventing complications.
		Vernal keratoconjunctivitis (VKC) itself typically does not directly cause blindness. However, in rare cases, if left untreated or if complications arise, it can lead to vision impairment. Here's a breakdown of the risks: Vision loss: While uncommon, VKC can cause vision loss in a few ways: Corneal scarring: Chronic inflammation in severe or uncontrolled VKC can damage the cornea, the clear dome at the front of the eye. Scarring on the cornea can blur vision. Complications from treatment: In some severe cases, long-term use of corticosteroids, a medication sometimes used to manage VKC, can increase the risk of cataracts or glaucoma, which can affect vision. Here's what you can do to minimize the risk of vision loss: Early diagnosis and treatment: Consulting an ophthalmologist (eye doctor) as soon as you suspect VKC is crucial. Early intervention with appropriate management strategies can significantly reduce the risk of complications. Treatment adherence: Following the treatment plan prescribed by your ophthalmologist is essential. This might involve medications, eye hygiene practices, and avoiding triggers. Regular eye checkups: Scheduling regular eye exams with your ophthalmologist allows them to monitor the condition, adjust treatment as needed, and detect any potential complications early on. If you have any concerns about vision loss or complications from VKC, discuss them with your ophthalmologist. They can provide personalized guidance based on your specific situation and the severity of your VKC. Remember: VKC is a manageable condition. With proper diagnosis, treatment, and adherence to recommendations, the risk of vision loss is very low.
		Vernal keratoconjunctivitis (VKC) itself is unlikely to cause complete blindness. However, in rare cases, if left untreated or improperly managed, it can lead to complications that can significantly impair vision. Here's a breakdown of the risks: Vision impairment, not blindness: While severe VKC can cause vision problems, complete blindness is uncommon. Potential complications: Untreated VKC, especially severe cases, can lead to complications like: Corneal ulcers: Open sores on the cornea that can cause scarring and vision problems. Corneal perforation: A hole in the cornea, which is a serious eye emergency. Glaucoma: Increased pressure within the eye that can damage the optic nerve and lead to vision loss. Cataracts: Clouding of the lens of the eye that can blur vision. Importance of treatment: Early diagnosis and proper management of VKC with an ophthalmologist is crucial to: Reduce the risk of complications: Following a treatment plan can significantly decrease the chances of developing vision-threatening problems. Maintain good vision: By controlling inflammation and preventing complications, VKC's impact on vision can be minimized. Seek medical attention: If you experience any symptoms suggestive of VKC, don't hesitate to consult an ophthalmologist. Early intervention is key to preventing complications and protecting your vision.
		Vernal keratoconjunctivitis (VKC) itself is unlikely to cause complete blindness. However, in rare cases, if left untreated or improperly managed, it can lead to complications that could potentially impair vision significantly. Here's a breakdown of the risks and how to manage them: Low Blindness Risk: VKC primarily causes itching, redness, tearing, and light sensitivity. These symptoms, although bothersome, don't usually lead to blindness. Potential Complications: If VKC is severe and not adequately controlled, it can increase the risk of developing certain eye problems that can affect vision, such as: Corneal ulcers: These are open sores on the cornea, the clear dome at the front of the eye. They can cause pain, blurry vision, and scarring. Corneal scarring: If corneal ulcers heal improperly, they can leave scar tissue behind. This scarring can significantly affect vision. Glaucoma: In rare cases, severe VKC can contribute to glaucoma, a condition that damages the optic nerve and can lead to vision loss. Importance of Management: Early diagnosis and proper treatment with an ophthalmologist (eye doctor) are crucial to: Manage symptoms and improve comfort. Reduce the risk of complications that could potentially affect vision. Early intervention is key: By seeking professional medical attention and adhering to the prescribed treatment plan, you can significantly reduce the risk of vision-threatening complications from VKC. Overall, the risk of blindness from VKC is very low with proper management. If you experience any symptoms suggestive of VKC, consult an ophthalmologist for prompt diagnosis and treatment.
13	Can you have a normal life with vernal keratoconjunctivitis?	Yes, in most cases, you can absolutely have a normal life with vernal keratoconjunctivitis (VKC). Here's a breakdown of why VKC doesn't typically hinder a normal life: Manageable symptoms: With proper diagnosis and treatment, the symptoms of VKC, such as itching, tearing, and light sensitivity, can be effectively managed. This allows individuals to participate in normal activities with minimal disruption. Self-limiting disease: For most people, VKC is a self-limiting disease, meaning the symptoms typically improve or resolve spontaneously around puberty. This means that even if symptoms cause some discomfort during childhood, they are unlikely to persist throughout life. Rare complications: Serious complications from VKC, like corneal scarring that can significantly impact vision, are rare, especially with proper management. Here are some additional factors to consider for living a normal life with VKC: Importance of treatment: Following a doctor's recommendations regarding medication, cold compresses, and allergen avoidance can significantly improve your quality of life and prevent complications. Potential adjustments: During flare-ups, you might need to make minor adjustments to your daily routine, such as limiting outdoor activities on high-pollen days or using lubricating eye drops more frequently. However, these adjustments are usually temporary and shouldn't significantly impact your overall lifestyle. Individual variations: The severity of VKC symptoms can vary from person to person. Some may experience mild, infrequent flare-ups, while others might have more frequent or intense symptoms. However, even in more severe cases, effective management strategies can still enable a normal life. Overall, with proper diagnosis, treatment, and potentially some minor adjustments during flare-ups, VKC does not have to prevent you from living a normal and fulfilling life. If you have VKC or are concerned about your child's condition, consulting with an ophthalmologist can help you develop a personalized management plan and address any specific concerns you may have.
		Yes, in most cases, you can absolutely have a normal life with vernal keratoconjunctivitis (VKC). Here's why: Manageable condition: VKC is a chronic allergic eye disease, but it's generally well-managed with proper treatment. Effective treatments: Ophthalmologists have a range of treatment options available, including: Artificial tears and lubricants: Soothe dry and irritated eyes. Antihistamine eye drops: Reduce allergic reactions and inflammation. Mast cell stabilizers: Prevent the release of inflammatory substances. Steroid eye drops (used cautiously): Reduce severe inflammation. Immunomodulators (for severe cases): Regulate the immune system. Favorable prognosis: For most children, VKC improves or resolves around puberty. Even in adults, VKC can be effectively managed to minimize symptoms and prevent complications. Normal life: With proper treatment, VKC shouldn't significantly impact your daily activities or overall well-being. You can participate in sports, hobbies, and social activities without restrictions in most cases. Importance of management: Regular checkups with your ophthalmologist are crucial to: Monitor your VKC. Adjust treatment as needed. Prevent complications. Living with VKC: While VKC can be bothersome, it doesn't have to define your life. By following your doctor's recommendations and managing your condition effectively, you can live a normal and fulfilling life. Here are some additional points to consider: Individual experiences: The severity of VKC symptoms can vary from person to person. Some people might experience occasional mild discomfort, while others might have more frequent and bothersome symptoms. Importance of communication: Open communication with your doctor is key to managing your VKC and addressing any concerns you might have. If you have VKC, remember you are not alone. With proper treatment and management, you can lead a normal and active life.
		Yes, in most cases, you can absolutely have a normal life with vernal keratoconjunctivitis (VKC). Here's a breakdown of why VKC typically doesn't impede a normal life: Treatable condition: VKC is a manageable disease. With proper diagnosis and treatment from an ophthalmologist (eye doctor), most people experience significant relief from symptoms. Self-limiting: In most cases, particularly in children, VKC is a self-limiting condition, meaning it improves with time and often resolves by puberty. Effective management: Even in adults where VKC might persist, effective management strategies like medications, eye hygiene, and avoiding triggers can significantly control symptoms and minimize discomfort. Here's what a normal life with VKC might look like: Participating in activities: With proper management, you can participate in most activities you enjoy, including sports and outdoor hobbies. Maintaining good vision: Early diagnosis and treatment can help prevent complications that might affect vision. Quality of life: Following the prescribed treatment plan and managing triggers can significantly improve your quality of life and reduce the impact of VKC on your daily routine. However, it's important to consider these points: Severity of VKC: In severe cases with frequent flare-ups, managing symptoms might require more effort and potentially limit some activities during flare-ups. Discomfort: The itching and other symptoms associated with VKC can be bothersome and disrupt sleep or daily activities. However, following treatment plans effectively reduces these discomforts. Here are some tips for living a normal life with VKC: See an ophthalmologist: Get a proper diagnosis and discuss treatment options. Follow the treatment plan: Adhere to the medications, eye hygiene practices, and trigger avoidance strategies recommended by your ophthalmologist. Schedule regular eye checkups: Maintain regular appointments with your ophthalmologist to monitor the condition and adjust treatment as needed. Communicate with your doctor: Discuss any concerns you have about VKC or its impact on your life. By following these tips and working with your ophthalmologist, you can effectively manage VKC and live a normal, fulfilling life.
		Yes, in most cases, you can absolutely have a normal life with vernal keratoconjunctivitis (VKC), especially with proper diagnosis and management. Here's why: Treatable condition: VKC is a treatable condition. Following a treatment plan prescribed by your ophthalmologist can significantly alleviate symptoms like itching, redness, and tearing. This allows you to enjoy a good quality of life with minimal disruption to your daily activities. Self-limiting in children: For most children, VKC is self-limiting, meaning the symptoms often improve or resolve around puberty. This allows them to participate fully in activities and social interactions. Manageable in adults: While less common, VKC can occur in adults. Even in these cases, proper management strategies can effectively control symptoms and prevent complications, allowing adults to maintain a normal life. Here are some key points to remember for a normal life with VKC: Early diagnosis and treatment: This is crucial for managing symptoms effectively and preventing complications. Following your doctor's recommendations: This includes using prescribed medications consistently and avoiding identified triggers. Regular eye checkups: Monitoring your condition with your ophthalmologist is essential to ensure proper control and address any potential issues. Living with VKC: Daily activities: With proper management, VKC shouldn't significantly impact daily activities like attending school, work, or participating in sports and hobbies. Social interactions: Effective symptom control allows for normal social interactions without discomfort or limitations. While VKC can be bothersome, it doesn't have to prevent you from living a normal life. By working with your ophthalmologist and following a treatment plan, you can effectively manage your symptoms and maintain good vision. Additional tips: Identify and avoid triggers: If you can identify specific allergens that worsen your VKC, try to avoid them whenever possible. This can further minimize symptoms and improve your quality of life. Over-the-counter options: In addition to prescribed medications, over-the-counter artificial tears can provide additional soothing relief for dry and irritated eyes. Remember, communication with your ophthalmologist is key. Don't hesitate to discuss any concerns you have about managing VKC and living a normal life.
		Yes, in most cases, you can absolutely have a normal life with vernal keratoconjunctivitis (VKC). Here's why: Manageable Condition: VKC is a treatable condition. With proper diagnosis and management by an ophthalmologist (eye doctor), most individuals can effectively control symptoms and prevent complications. Effective Treatments: Various medications and strategies can significantly alleviate symptoms like itching, redness, and tearing, allowing for normal daily activities. Self-Limiting Nature: Particularly in children, VKC often resolves on its own around puberty. Even in adults, proper treatment can ensure a normal life. Here's a breakdown of factors contributing to a normal life with VKC: Early Diagnosis and Treatment: Seeking professional medical attention at the first sign of symptoms is crucial. This allows for prompt initiation of treatment and reduces the risk of complications. Adherence to Treatment Plan: Following the prescribed medications and eye care regimen as instructed by your ophthalmologist is essential for optimal symptom control. Lifestyle Modifications: Depending on the severity and triggers, some lifestyle adjustments might be helpful, such as avoiding allergens or using cold compresses on the eyes. Regular Eye Checkups: Scheduling regular eye checkups with your ophthalmologist allows for monitoring progress and adjusting treatment as needed. Living with VKC: While VKC can be bothersome, it shouldn't significantly restrict your ability to live a normal life. Here are some additional points to consider: Impact on Activities: During periods of worsening symptoms, certain activities like spending time outdoors during high pollen seasons or wearing contact lenses might need temporary adjustments. Emotional Well-being: The discomfort and irritation caused by VKC can sometimes affect emotional well-being, particularly in children. Open communication with your doctor and exploring support groups can be helpful. Overall, with proper diagnosis, treatment, and management, most people with VKC can lead normal, active lives without significant limitations. If you have VKC or suspect you might, consult an ophthalmologist to discuss the best course of action for you.
14	What can I do to prevent vernal keratoconjunctivitis?	Vernal keratoconjunctivitis (VKC) is an allergic eye condition, and unfortunately, there's no guaranteed way to prevent it completely. However, there are steps you can take to reduce the risk of flare-ups and manage symptoms if your child develops VKC: Minimize allergen exposure: Identify triggers: If possible, try to identify potential allergens that might trigger your child's VKC. Common triggers include pollen, dust mites, pet dander, and mold. Pollen control: During high-pollen seasons, keep windows closed and use air conditioning with air filters. Consider pollen forecasts and limit outdoor activities on high-pollen days. Dust mite control: Wash bedding in hot water (at least 55°C or 130°F) weekly. Encase mattresses and pillows in allergen-proof covers. Regularly vacuum carpets and furniture with a HEPA filter. Pet dander control: If you have pets, bathe them regularly and keep them out of bedrooms. Consider allergy medication for pets if appropriate. Mold control: Address any mold problems in your home promptly. Maintain proper ventilation in bathrooms and kitchens to prevent moisture buildup. General preventative measures: Eye hygiene: Encourage your child to wash their hands frequently to avoid transferring allergens to their eyes. Teach them to avoid rubbing their eyes, which can worsen irritation. Cold compresses: Applying cool compresses to the eyes for a few minutes at a time can help soothe itching and inflammation. Sunglasses: Wearing sunglasses that block ultraviolet (UV) rays can help protect the eyes from irritants and light sensitivity. Remember: Consult your ophthalmologist: Discuss these preventative measures with your child's ophthalmologist. They can provide personalized recommendations based on your child's specific triggers and situation. Management is key: Even with preventative measures, flare-ups can still occur. Following a doctor-recommended treatment plan is crucial to manage symptoms effectively and improve your child's comfort. By implementing these strategies, you can help reduce the risk of VKC flare-ups and improve your child's quality of life. While there's no guaranteed way to prevent vernal keratoconjunctivitis (VKC), certain strategies can help reduce your child's risk of flare-ups or lessen the severity of symptoms: Minimize allergen exposure: Identify triggers: If possible, try to identify specific allergens like pollen, dust mites, or pet dander that might trigger your child's VKC. Pollen control: During peak pollen seasons, keep windows closed and use air conditioning with HEPA filters. Dust mite control: Encase mattresses and pillows in allergen-proof covers, wash bedding frequently in hot water, and reduce dust-collecting items like carpets and stuffed animals. Pet dander control: If you have pets, bathe them regularly, vacuum frequently, and keep them out of bedrooms. Eye hygiene: Cold compresses: Applying cold compresses to your child's eyes several times a day can help reduce inflammation and itching. Artificial tears: Regular use of artificial tears can help lubricate the eyes and soothe irritation. Eyelid hygiene: Gently clean your child's eyelids with a mild cleanser to remove allergens and irritants. Other recommendations: Sunglasses: Encourage your child to wear sunglasses with UV protection to shield their eyes from sunlight and irritants like dust or wind. Avoid eye rubbing: Rubbing can worsen inflammation and irritation. Teach your child alternative ways to soothe itchy eyes, like gentle blinking. Air purifiers: Air purifiers with HEPA filters can help reduce airborne allergens in your child's environment. Talk to your doctor: Discuss any additional preventive measures or medications that might be helpful for your child's specific case. Remember: These are general recommendations, and the most effective preventive approach will depend on your child's individual triggers and sensitivities. Consulting an ophthalmologist is crucial to create a personalized plan to manage your child's VKC and minimize the risk of flare-ups. By implementing these strategies and working with your doctor, you can help your child experience fewer and less severe VKC flare-ups, improving their overall comfort and well-being.
		Unfortunately, there's no guaranteed way to prevent vernal keratoconjunctivitis (VKC) entirely. However, there are several strategies that can help reduce your risk or minimize the severity of flare-ups: Minimize allergen exposure: Identify triggers: If you suspect allergens play a role in your VKC, try to identify your specific triggers through allergy testing or by keeping a symptom journal. Common triggers include pollen, dust mites, and pet dander. Pollen avoidance: During high pollen seasons, keep windows closed and use air conditioning with HEPA filters. Consider wearing a pollen mask when outdoors. Dust mite control: Encase mattresses and pillows in allergen-proof covers, wash bedding frequently in hot water, and vacuum regularly with a HEPA filter. Pet dander control: If you have pets, bathe them regularly, brush them frequently outdoors, and avoid close contact during flare-ups. Eye hygiene practices: Cold compresses: Applying cool compresses to your eyes for a few minutes several times a day can help soothe itching and inflammation. Artificial tears: Using artificial tears throughout the day can help lubricate your eyes and reduce irritation. Avoid eye rubbing: Rubbing your eyes can worsen inflammation and irritation. Lifestyle modifications: Limit smoke and irritants: Avoid exposure to smoke, dust, and other airborne irritants that can exacerbate VKC symptoms. Sunglasses: Wearing sunglasses that block ultraviolet (UV) rays can help protect your eyes from sunlight, which can sometimes trigger symptoms. Early diagnosis and treatment: See an ophthalmologist: If you experience symptoms suggestive of VKC, consult an ophthalmologist for a proper diagnosis and discuss potential treatment options. Early intervention can help prevent complications and manage future flare-ups more effectively. Remember: These are general recommendations, and the most effective approach might vary depending on your individual triggers and the severity of your VKC. It's crucial to discuss these strategies with your ophthalmologist to develop a personalized plan to manage your VKC and improve your quality of life.
		While there's no guaranteed way to prevent vernal keratoconjunctivitis (VKC) completely, several strategies can help reduce the risk of developing symptoms or experiencing flare-ups, especially if you have a family history of allergies or atopic conditions: Minimize allergen exposure: Identify triggers: If possible, try to identify specific allergens like pollen, dust mites, or mold that worsen your symptoms. This might involve keeping a symptom journal to track potential triggers. Pollen avoidance: During high pollen seasons, keep windows closed, use air conditioning with air filters, and limit outdoor activities when pollen counts are high. Dust mite control: Regularly wash bedding in hot water (at least 55°C or 130°F) and use mite-proof covers for pillows and mattresses. Vacuum frequently and remove dust-collecting items like stuffed animals from bedrooms. Mold control: Address any sources of mold in your home and maintain proper ventilation to reduce humidity levels. General preventative measures: Eye hygiene: Maintain good eye hygiene by washing your hands frequently and avoiding rubbing your eyes, which can introduce allergens or irritants. Cold compresses: Applying cool compresses to your closed eyelids for short periods can provide temporary relief from itching and inflammation. Artificial tears: Using preservative-free artificial tears throughout the day can help lubricate and soothe dry, irritated eyes. Lifestyle modifications: Omega-3 fatty acids: Some studies suggest that consuming foods rich in omega-3 fatty acids, like fatty fish, might have anti-inflammatory properties and potentially offer some benefit. Consult your doctor: Discuss these lifestyle modifications with your healthcare professional to determine if they are suitable for you. Remember: Early diagnosis and management: If you experience any symptoms suggestive of VKC, consult an ophthalmologist for a proper diagnosis and discuss the most suitable treatment plan to manage your specific case. Ongoing management: Even with preventative measures, VKC flare-ups can still occur. Following your doctor's recommendations and being prepared to manage symptoms is crucial. By implementing these strategies and working with your healthcare professional, you can potentially reduce your risk of developing VKC or experiencing severe flare-ups, allowing you to enjoy a better quality of life.
		Unfortunately, there's no guaranteed way to prevent vernal keratoconjunctivitis (VKC) entirely, as it's likely caused by a combination of genetic and environmental factors. However, there are steps you can take to reduce your risk of triggering symptoms or worsening an existing case: Minimize Allergen Exposure: Identify Triggers: If you know what triggers your allergies (pollen, dust mites, pet dander, etc.), try to minimize exposure. Pollen Season: During high pollen seasons, keep windows closed, use air conditioning with air filters, and limit outdoor activities when pollen counts are high. Dust Mites: Wash bedding in hot water frequently, use dust mite-proof mattress covers, and vacuum regularly with a HEPA filter. Pet Dander: If you're allergic to pet dander, bathe pets regularly, keep them off furniture, and avoid close contact during flare-ups. Eye Care Practices: Cold Compresses: Apply cool compresses to your eyes several times a day to soothe irritation. Artificial Tears: Use lubricating eye drops (artificial tears) to keep your eyes moist and comfortable. Avoid Rubbing: Rubbing your eyes can worsen irritation. Try to resist the urge and use artificial tears instead. Eyewear: Wearing sunglasses that block ultraviolet (UV) rays and wrap around the face can help shield your eyes from allergens and irritants. Lifestyle Modifications: Smoke and Air Pollution: Avoid smoky environments and areas with high air pollution levels, as these can irritate your eyes. Handwashing: Wash your hands frequently to avoid transferring allergens to your eyes. Contact Lenses: If you wear contact lenses, consult your ophthalmologist (eye doctor) to discuss if they might worsen your VKC and explore alternative options like eyeglasses. Remember, these are general recommendations. It's crucial to consult an ophthalmologist for personalized advice based on your specific triggers and condition severity. They can create a tailored management plan to help you effectively manage VKC and improve your quality of life.
15	How can vernal keratoconjunctivitis be treated?	Vernal keratoconjunctivitis (VKC) doesn't have a single cure, but there are effective treatments available to manage symptoms and prevent complications. Here's an overview of the treatment options: Medications: Antihistamines: These medications help reduce itching, a hallmark symptom of VKC. They come in various forms like eye drops or oral tablets. Mast cell stabilizers: These eye drops work by preventing the release of inflammatory chemicals from mast cells, which play a role in allergic reactions. They are typically used long-term to prevent flare-ups. Corticosteroids: In severe cases, ophthalmologists might prescribe short-term topical corticosteroids to manage inflammation. However, due to potential side effects like increased eye pressure or cataracts, they are used cautiously and for a limited duration. Other treatments: Cold compresses: Applying cool compresses to the eyes for a few minutes at a time can help alleviate itching and swelling. Artificial tears: Lubricating eye drops can help soothe dryness and irritation. Allergen avoidance: Identifying and avoiding potential allergens that trigger your child's VKC can significantly reduce flare-ups. Immunosuppressants: In very rare and severe cases, ophthalmologists might consider immunosuppressant medications to suppress the immune system and reduce inflammation. Treatment plan: Personalized approach: The ophthalmologist will develop a treatment plan tailored to the severity of your child's VKC and their individual needs. Severity considerations: For mild cases, eyedrops like antihistamines or mast cell stabilizers might be sufficient. In severe cases, a combination of medications and other strategies might be necessary. Long-term management: VKC is often a chronic condition, and treatment may be needed for an extended period, especially during allergy seasons or if triggers cannot be fully avoided. Here are some additional points to remember: Early diagnosis is key: Seeking medical attention promptly allows for early intervention and prevents complications. Follow doctor's instructions: It's crucial to adhere to the ophthalmologist's recommendations regarding medication use, frequency, and duration for optimal results and to minimize side effects. Regular checkups: Schedule regular follow-up appointments with the ophthalmologist to monitor your child's progress and adjust the treatment plan as needed. By working with an ophthalmologist and following a personalized treatment plan, you can effectively manage your child's VKC and improve their quality of life.
		Vernal keratoconjunctivitis (VKC) treatment focuses on managing symptoms and preventing complications. The specific approach will depend on the severity of your child's case. Here's a breakdown of common treatment options: Non-steroidal medications: Artificial tears: These lubricating eye drops help relieve dryness and irritation, a hallmark symptom of VKC. They can be used frequently throughout the day. Antihistamine eye drops: These medications block the release of histamine, a chemical released during allergic reactions that contributes to inflammation and itching. Mast cell stabilizers: These medications prevent mast cells (immune system cells) from releasing inflammatory substances, reducing inflammation and itching. They are typically used long-term to prevent flare-ups. Corticosteroids (used cautiously): Corticosteroid eye drops are powerful anti-inflammatory medications. However, due to potential side effects with prolonged use, they are typically reserved for severe cases or short-term management of acute flare-ups. Immunomodulators (for severe cases): In rare cases of severe or persistent VKC, medications that regulate the immune system might be considered. These are typically used by ophthalmologists specializing in immunology or allergy. Additional tips: Cold compresses: Applying cold compresses to the eyes several times a day can provide temporary relief from inflammation and itching. Eyelid hygiene: Gently cleaning your child's eyelids with a mild cleanser can remove allergens and irritants that may worsen symptoms. Importance of consulting an ophthalmologist: A qualified ophthalmologist can diagnose VKC, differentiate it from other conditions, and recommend the most appropriate treatment plan based on your child's individual needs and severity. They can also monitor your child's progress and adjust the treatment plan as needed. Here are some resources you may find helpful: American Academy of Ophthalmology: https://www.aao.org/education/disease-review/vernal-keratoconjunctivitis-5 National Eye Institute: https://www.nei.nih.gov/ Remember, early diagnosis and proper management are crucial to improve your child's comfort, prevent complications, and ensure optimal vision health.
		Vernal keratoconjunctivitis (VKC) doesn't have a single cure, but there are effective treatment strategies to manage symptoms and prevent complications. Here's a breakdown of the common treatment approaches: 1. Artificial Tears: Function: Artificial tears lubricate the eyes, soothe irritation, and wash away allergens that may trigger symptoms like itching and burning. 2. Cold Compresses: Function: Applying cool compresses to closed eyelids for a few minutes at a time can help reduce inflammation and provide temporary relief from itching and discomfort. 3. Antihistamines: Function: Antihistamines come in eye drop and oral medication forms. They work by blocking the action of histamine, a chemical released by the body during an allergic reaction, which helps alleviate itching and redness. 4. Mast Cell Stabilizers: Function: Mast cells are immune system cells that release histamine. Mast cell stabilizers help prevent these cells from releasing histamine, thereby reducing inflammation and itching. They typically require a few weeks of consistent use to show their full effect. 5. Corticosteroids: Function: Corticosteroids are powerful anti-inflammatory medications. They are typically reserved for severe cases or short-term use due to potential side effects like increased eye pressure with prolonged use. Here's a note on treatment selection: The specific medications and their combinations will be chosen by your ophthalmologist (eye doctor) based on the severity of your VKC and your individual needs. Additional Management Strategies: Allergen avoidance: Identifying and avoiding triggers like pollen, dust mites, or pet dander can significantly reduce flare-ups. Eyelid hygiene: Regularly cleaning your eyelids with a gentle cleanser can help remove allergens and irritants. Sunglasses: Wearing sunglasses that block UV rays can protect your eyes from sunlight, which can sometimes worsen symptoms. It's important to remember: Treatment adherence: Following the treatment plan prescribed by your ophthalmologist is crucial for optimal outcomes. This might involve using medications regularly, practicing good eye hygiene, and avoiding triggers. Regular eye checkups: Scheduling regular appointments with your ophthalmologist allows them to monitor the condition, adjust treatment as needed, and detect any potential complications early on. If you have any concerns about VKC treatment options or their side effects, discuss them openly with your ophthalmologist. They can provide personalized guidance based on your specific situation.
		Vernal keratoconjunctivitis (VKC) doesn't have a cure, but there are effective treatment strategies to manage symptoms and prevent complications. The specific treatment plan will depend on the severity of your case and your doctor's recommendations. Here's a breakdown of common treatment approaches: Artificial Tears: Provide lubrication and soothe dry, irritated eyes. Available over-the-counter in various forms (drops, gels, ointments). Can be used frequently throughout the day, especially during dry or irritating conditions. Antihistamine Eye Drops: Help reduce itching and inflammation caused by allergic reactions. Available as over-the-counter or prescription options, depending on the severity. May cause temporary side effects like blurred vision or burning sensation. Corticosteroid Eye Drops: Powerful anti-inflammatory medication reserved for severe cases or those unresponsive to other treatments. Use with caution due to potential side effects like increased eye pressure or cataracts with prolonged use. Close monitoring by your ophthalmologist is crucial if prescribed. Cold Compresses: Offer temporary relief from itching and inflammation. Apply a cool compress to closed eyelids for short periods (around 10-15 minutes) at a time. Ensure proper hygiene to avoid introducing bacteria into the eyes. Allergy Management (if applicable): Identifying and avoiding specific triggers like pollen, dust mites, or mold can significantly reduce symptoms. This might involve using air purifiers, maintaining good home hygiene practices, and minimizing outdoor activities during high pollen seasons. Immunomodulators (for severe cases): Medications like cyclosporine A may be used in rare cases to suppress the underlying immune response in severe VKC unresponsive to other treatments. Due to potential side effects, these medications are typically reserved for severe cases under close supervision by an ophthalmologist. Additional Tips: Over-the-counter pain relievers: In some cases, over-the-counter pain relievers like ibuprofen can help manage discomfort associated with VKC. However, always consult your doctor before using any new medication. Lifestyle modifications: Maintaining good eye hygiene, getting enough sleep, and managing stress might contribute to overall eye health and potentially reduce VKC symptoms. Discuss these modifications with your doctor. Remember: Early diagnosis and treatment are crucial to prevent complications like corneal scarring, which can impair vision. Consistency with your prescribed treatment plan is essential for optimal symptom control. Regular follow-up appointments with your ophthalmologist are necessary to monitor your condition and adjust the treatment plan as needed. If you have any concerns about VKC or its treatment, don't hesitate to consult your healthcare professional. They can provide personalized advice based on your specific situation.
		Vernal keratoconjunctivitis (VKC) treatment focuses on managing symptoms and preventing complications. The specific approach will vary depending on the severity of your case and your doctor's recommendations. Here's a breakdown of the most common treatment options: Medications: Artificial Tears: These lubricating eye drops help soothe irritation and dryness caused by VKC. Mast Cell Stabilizers: These medications prevent the release of inflammatory chemicals from mast cells in the eye, reducing itching and inflammation. Topical Antihistamines: These medications target histamine, another key player in the allergic response, further reducing itching and redness. Corticosteroids: In severe cases, corticosteroids may be prescribed for short-term relief due to their potent anti-inflammatory properties. However, long-term use should be avoided due to potential side effects. Cyclosporine A: This medication is sometimes used in severe cases that don't respond well to other treatments. It suppresses the immune system and reduces inflammation. Other Strategies: Cold Compresses: Applying cool compresses to your eyes several times a day can help reduce inflammation and discomfort. Avoid Rubbing: Rubbing your eyes can worsen irritation. Try to resist the urge and use artificial tears instead. Eyewear: Wearing sunglasses that block ultraviolet (UV) rays and wrap around the face can help shield your eyes from allergens and irritants. In some cases, additional interventions might be necessary: Allergy Testing: If the specific allergens are unknown, allergy testing can help identify triggers and guide further management strategies. Immunotherapy: In rare cases, allergy shots (immunotherapy) might be considered for long-term management of severe VKC. Here are some additional points to consider: Early Diagnosis and Treatment: Seeking professional medical attention at the first sign of symptoms is crucial. This allows for prompt initiation of treatment and reduces the risk of complications. Adherence to Treatment Plan: Following the prescribed medications and eye care regimen as instructed by your ophthalmologist is essential for optimal symptom control. Long-Term Management: VKC can be a chronic condition, and ongoing management might be necessary, especially in adults. Remember, this information is intended for general knowledge only. It's vital to consult an ophthalmologist (eye doctor) for a proper diagnosis and personalized treatment plan for your specific case of VKC.
16	What type of surgeries are available for vernal keratoconjunctivitis?	Surgery is typically not the first-line treatment for vernal keratoconjunctivitis (VKC) because most cases respond well to medication and other management strategies. However, in some rare and severe cases, surgery may be considered as a last resort if other approaches fail to control the symptoms or prevent complications. Here's a breakdown of potential surgical interventions for VKC: Limited surgical role: Not a primary treatment: Surgery is generally not recommended for most VKC cases. Ophthalmologists will primarily focus on managing symptoms with medications, cold compresses, and allergen avoidance strategies. Potential surgical options: Excision of giant papillae: In severe cases with large, inflamed bumps (giant papillae) on the inner surface of the upper eyelid that are causing significant discomfort or vision problems, an ophthalmologist might consider surgical excision. This procedure involves removing the papillae to reduce inflammation and improve symptoms. Conjunctival scraping: In very rare cases with severe corneal involvement and persistent inflammation despite other treatments, conjunctival scraping might be an option. This procedure involves removing a thin layer of the conjunctiva (the white part of the eye) to reduce inflammatory cells and potentially improve symptoms. Considerations for surgery: Reserved for severe cases: Surgery is only considered when other treatments have failed to provide adequate relief, and the potential benefits outweigh the risks. Careful evaluation: An ophthalmologist will carefully evaluate the severity of the condition, the potential risks and benefits of surgery, and any alternative treatment options before recommending surgery. Success rates: The success rates of surgical interventions for VKC can vary depending on the specific procedure and the severity of the case. However, these procedures are not always curative, and there's a chance of recurrence. Overall: Surgery for VKC is uncommon. Medication and other management strategies are usually the preferred approach. If surgery is considered, a thorough evaluation by an ophthalmologist is crucial to determine if it's the right course of action for your specific case. It's important to remember that this information is intended for general knowledge only and shouldn't be a substitute for professional medical advice. If you have any questions or concerns about surgical options for your child's VKC, consulting with their ophthalmologist is essential. They can provide the most up-to-date and personalized recommendations based on your child's specific situation and medical history.
		Surgery is typically not the first-line treatment for vernal keratoconjunctivitis (VKC) because most cases can be effectively managed with medications and other conservative measures. However, in rare and severe cases, surgery might be considered as a last resort. Here's a breakdown of the situation: Limited surgical role: VKC is primarily treated with medications like antihistamines, mast cell stabilizers, and corticosteroids (used cautiously) to manage inflammation and symptoms. Surgical scenarios: Surgery for VKC is reserved for severe cases that are unresponsive to medical therapy and where complications threaten vision. The specific surgical approach depends on the presenting features of the VKC. Potential surgical options: Excision of giant papillae: In some cases, large, inflamed bumps (giant papillae) on the underside of the eyelid can be surgically removed to reduce mechanical irritation on the cornea. This might be combined with: Mitomycin C: A medication applied during surgery to prevent scar tissue formation. Amniotic membrane transplantation: Transplanting a thin layer of tissue from the amniotic sac to promote healing and reduce inflammation. Corneal procedures (rare): In severe cases with corneal involvement like ulcers or scarring, corneal surgeries like keratoplasty (corneal transplant) might be considered to restore vision. Importance of consulting an ophthalmologist: A qualified ophthalmologist can assess the severity of your child's VKC and determine if surgery is a viable option. They will discuss the risks and benefits of surgery in detail and recommend the most appropriate course of action for your child's specific case. Remember: Surgical intervention for VKC is uncommon and only considered in severe cases that don't respond to other treatments. Early diagnosis and proper management with medications can help prevent the need for surgery.
		Surgery for vernal keratoconjunctivitis (VKC) is generally not the first-line treatment and is typically reserved for severe cases that are unresponsive to other management strategies. Here's a breakdown of why surgery might be considered and what procedures are involved: When is surgery considered? Severe, uncontrolled VKC: If medication, cold compresses, and allergen avoidance fail to adequately control symptoms despite consistent use, surgery might be an option. Complications: In rare cases, severe VKC can lead to corneal complications like scarring or ulcers. Surgery might be necessary to address these complications and restore vision. Types of surgery for VKC: Excision of giant papillae: In some cases, large, inflamed bumps (giant papillae) on the inner surface of the upper eyelid can contribute significantly to symptoms. An ophthalmologist might recommend surgical removal of these papillae. Limbal vernal keratoconjunctivitis: For a rare form of VKC affecting the limbus (the border between the cornea and conjunctiva), surgical excision of the affected tissue might be considered. Conjunctival grafts: In severe cases with significant conjunctival scarring, a conjunctival graft from another part of the eye or from a donor might be used to replace the damaged tissue. Important points to consider about surgery for VKC: Not a first-line treatment: Surgery is typically a last resort after trying other management strategies first. Effectiveness: The long-term effectiveness of surgery for VKC can vary, and there's a chance of recurrence. Risks and complications: As with any surgery, there are potential risks and complications associated with VKC surgery, which your ophthalmologist will discuss with you in detail before proceeding. It's crucial to discuss the potential benefits and risks of surgery with your ophthalmologist to determine if it's the right course of action for your specific case. They can assess the severity of your VKC, explore all treatment options, and help you make an informed decision. Here's a reiteration: In most cases of VKC, effective management with medications, cold compresses, and allergen avoidance strategies can significantly improve symptoms and prevent complications. Surgery is usually considered only in severe or chronic cases that don't respond well to other treatments.
		Surgery is typically not the first-line treatment for vernal keratoconjunctivitis (VKC) because most cases can be effectively managed with medication and lifestyle modifications. However, in rare cases of severe VKC that is unresponsive to other treatments, surgery may be considered. Here's a breakdown of some potential surgical options: Excision of Giant Papillae: This procedure involves removing large, inflamed bumps (giant papillae) that develop on the underside of the eyelid in some cases of VKC. The removal can be done with different techniques, including scalpel blade, scissors, or radiosurgery. This surgery is usually performed along with other treatments like anti-inflammatory medications to manage the underlying allergic response. Conjunctival Grafting: This procedure involves transplanting healthy tissue from another part of the eye (usually the upper conjunctiva) to the affected area on the eyeball. This can be helpful in cases where the cornea (the clear front surface of the eye) has become damaged due to chronic inflammation from VKC. The new tissue helps to replace damaged tissue and promote healing. Amniotic Membrane Transplantation (AMT): This surgery involves transplanting a thin layer of tissue from the amniotic membrane (the sac that surrounds a developing fetus) onto the ocular surface. AMT has anti-inflammatory properties and can promote healing of the corneal surface in cases of VKC with corneal complications. It may be used in combination with other procedures like excision of giant papillae. Important points to remember about surgery for VKC: Reserved for severe cases: Surgery is considered a last resort for VKC, typically only used when other treatments have failed to control symptoms and prevent complications. Benefits and risks: Each surgical procedure has its own set of benefits and risks. Your ophthalmologist will discuss these details with you thoroughly before recommending surgery. Not a cure: Surgery for VKC aims to manage symptoms and prevent complications, but it doesn't cure the underlying allergic condition. If your doctor suggests surgery for VKC: Ask questions: Don't hesitate to ask your ophthalmologist about the specific procedure recommended, its potential benefits and risks, and alternative treatment options. Understand the recovery process: Inquire about the expected recovery time, post-operative care, and potential limitations after surgery. Remember: Early diagnosis and proper management of VKC with medication and lifestyle modifications can often prevent the need for surgery. Consulting with your ophthalmologist is crucial to determine the most suitable treatment approach for your specific case.
		Surgery for vernal keratoconjunctivitis (VKC) is generally considered a last resort, used only in severe cases that don't respond well to other treatment options. Here's a breakdown of why surgery is uncommon for VKC and the potential surgical approaches: Limited Role of Surgery: Effectiveness of Medications: In most cases, VKC can be effectively managed with medications, cold compresses, and lifestyle modifications. Self-Limiting Nature: Particularly in children, VKC often resolves on its own around puberty. Surgery is rarely necessary for such cases. Potential Surgical Options: If medication and other conservative measures fail to provide adequate relief in severe VKC, a qualified ophthalmologist (eye doctor) might consider surgical intervention. The specific type of surgery will depend on the presenting features: Excision of Giant Papillae: In some cases, large, inflamed bumps (giant papillae) on the inner eyelids can be surgically excised. This can be combined with intraoperative application of Mitomycin C, a medication that helps prevent regrowth. Limbal Keratoplasty: In rare cases with severe corneal involvement, limbal keratoplasty might be an option. This procedure involves transplanting healthy tissue from the limbus (the border between the cornea and the conjunctiva) to the affected cornea. Important Considerations: Risks and Benefits: As with any surgery, there are potential risks associated with VKC surgeries, such as infection, bleeding, and scarring. The ophthalmologist will carefully weigh the risks and benefits before recommending surgery. Not a Cure: Surgery is not a cure for VKC, but rather aims to address specific complications or improve symptoms that haven't responded to other treatments. Alternative Approaches: In some cases, other interventions like intense pulsed light (IPL) therapy might be explored for managing VKC, although more research is needed to establish their long-term effectiveness. Overall, surgery for VKC is uncommon. Early diagnosis and proper management with medications and lifestyle modifications can effectively control symptoms and prevent complications in most cases. If you have concerns about severe VKC that isn't responding to treatment, consult your ophthalmologist to discuss all potential treatment options, including the risks and benefits of surgery.
17	What is the spectrum of severity of vernal keratoconjunctivitis?	Vernal keratoconjunctivitis (VKC) can manifest in a range of severities, affecting individuals differently. Here's a breakdown of the spectrum of VKC severity: Grading Systems: There's no universally accepted single system for grading VKC severity. However, ophthalmologists often use a combination of factors to assess the condition's intensity: Ocular signs: This includes the presence, size, and location of papillae (bumps) on the inner eyelid, hyperemia (redness) of the conjunctiva, and any corneal involvement (e.g., punctate keratitis). Symptoms: The severity of itching, tearing, light sensitivity, foreign body sensation, and blurred vision are all considered. Here's a general classification system used by some ophthalmologists: Mild VKC: This involves minimal symptoms like occasional itching and tearing, with small papillae on the inner eyelid and minimal to no corneal involvement. Moderate VKC: This includes moderate itching, tearing, and light sensitivity, with prominent papillae and possible punctate keratitis (tiny corneal defects). Severe VKC: This involves significant discomfort with intense itching, tearing, light sensitivity, and blurred vision. There might be large, cobblestone-like papillae, significant corneal involvement (e.g., macroerosions), and potential vision impairment. Additional Considerations: Impact on daily life: The severity is also assessed based on how much VKC disrupts a person's daily activities and overall quality of life. Variations in Presentation: Seasonal: VKC symptoms often worsen during spring and summer months due to increased allergen exposure. Individual differences: Even within these classifications, individual experiences of VKC can vary. Some people might have infrequent flare-ups with mild symptoms, while others experience persistent and severe discomfort. Importance of Consultation: Diagnosis and Grading: An ophthalmologist can accurately diagnose VKC, assess its severity based on your child's specific presentation, and develop a personalized treatment plan. Monitoring Progress: Regular follow-up appointments allow the ophthalmologist to monitor the response to treatment and adjust the plan as needed. By understanding the spectrum of VKC severity and consulting with an ophthalmologist, you can ensure your child receives the most appropriate management for their specific condition.
		Vernal keratoconjunctivitis (VKC) exists on a spectrum of severity, ranging from mild and occasional discomfort to severe and vision-threatening. Here's a breakdown of the potential variations: Mild VKC: This is the most common presentation. Symptoms might include: Occasional itching and redness of the eyes. Tearing. Mild discomfort. Minimal impact on daily activities. Moderate VKC: Symptoms become more frequent and bothersome. This could include: Increased itching, redness, and tearing. Light sensitivity. Feeling of something foreign in the eye. Disruption of sleep due to eye discomfort. May require frequent use of artificial tears or antihistamine eye drops. Severe VKC: This is the least common but most concerning presentation. It's characterized by: Intense itching, redness, and tearing. Significant discomfort that can interfere with daily activities. Possible involvement of the cornea, leading to complications like ulcers or scarring. Potential vision impairment. May require a combination of medications, including corticosteroids (used cautiously) and immunomodulators in severe cases. Factors influencing severity: Allergic triggers: Exposure to allergens like pollen or dust mites can worsen symptoms. Individual immune response: Some people have a stronger allergic response, leading to more severe VKC. Age: Symptoms may improve or resolve around puberty in some cases. Importance of diagnosis and management: Regardless of severity, consulting an ophthalmologist is crucial for proper diagnosis and a tailored treatment plan. Early intervention can prevent complications like corneal damage and ensure optimal vision health. Here's an additional point to consider: Some classification systems for VKC severity combine subjective symptoms (itching, discomfort) with objective signs observed by an ophthalmologist (papillae, corneal involvement) to create a comprehensive assessment. By understanding the spectrum of VKC severity and seeking professional guidance, you can ensure your child receives the most appropriate treatment to manage their symptoms and prevent complications.
		Vernal keratoconjunctivitis (VKC) can range from mild and seasonal to severe and chronic. The spectrum of severity is typically assessed based on a combination of factors, including: Symptoms: The intensity and frequency of symptoms like itching, redness, tearing, and foreign body sensation. Signs: The presence and extent of visible signs on the eye during an ophthalmological examination, such as giant papillae (inflamed bumps on the eyelid), corneal involvement (inflammation of the cornea), and limbal involvement (inflammation of the border between the cornea and conjunctiva). Impact on daily life: How much VKC disrupts daily activities and sleep due to discomfort. Here's a general breakdown of the VKC severity spectrum: Mild VKC: Symptoms: Occasional itching, mild redness, and watering of the eyes, primarily during allergy seasons. Signs: Limited presence of small papillae on the upper eyelid, minimal or no corneal involvement. Impact: Symptoms are mild and don't significantly disrupt daily activities. Moderate VKC: Symptoms: More frequent itching, redness, tearing, and foreign body sensation, with possible seasonal variations. Signs: Presence of moderate-sized papillae on the upper eyelid, possible punctate keratitis (tiny dots on the cornea) but no significant corneal scarring. Impact: Symptoms can be bothersome but may not severely disrupt daily life. Severe VKC: Symptoms: Intense, chronic itching, significant redness, tearing, and light sensitivity that can worsen during flare-ups. Signs: Large, inflamed giant papillae on the upper eyelid, possible corneal involvement with ulceration or scarring, and potential limbal involvement. Impact: Symptoms can be debilitating and significantly disrupt daily activities and sleep. Additionally: Classification systems: Ophthalmologists may use specific classification systems to assess VKC severity, considering both ocular signs and symptoms. These systems assign scores based on the severity of different aspects, allowing for a more objective evaluation. The distinction between moderate and severe VKC can vary depending on the specific classification system used and the ophthalmologist's assessment. It's important to note: This is a general overview, and the severity of VKC can exist on a spectrum within these categories. Consulting an ophthalmologist is crucial for an accurate diagnosis and to determine the appropriate treatment plan based on the individual's specific presentation of VKC.
		Vernal keratoconjunctivitis (VKC) can manifest in a range of severities, typically classified based on the signs and symptoms a patient experiences. Here's a breakdown of the spectrum of VKC severity: Mild VKC: This is the most common form of VKC. Symptoms are usually manageable and may include: Mild itching and irritation in the eyes Occasional tearing Some redness in the conjunctiva (the white part of the eye) Possible presence of small, pale papillae (bumps) on the underside of the eyelid Moderate VKC: Symptoms are more pronounced than mild VKC but still manageable with proper treatment. May include: Increased itching and irritation More frequent tearing Greater redness in the conjunctiva Presence of larger and more numerous papillae Possible light sensitivity (photophobia) Severe VKC: This is the least common form but requires close attention and management by an ophthalmologist to prevent complications. Symptoms can be quite bothersome and may include: Intense, constant itching and irritation Frequent and excessive tearing Significant redness and inflammation in the conjunctiva Presence of giant papillae (large, inflamed bumps) on the underside of the eyelid Corneal involvement: This can manifest as punctate epithelial erosions (tiny scratches on the cornea), shield ulcers (larger, more serious corneal ulcers), or corneal neovascularization (growth of blood vessels onto the cornea). Blurred vision Grading Systems: Several grading systems exist to categorize VKC severity based on specific clinical features. These systems often consider a combination of: Symptoms: Intensity of itching, tearing, and light sensitivity Signs: Degree of conjunctival hyperemia (redness), presence and size of papillae, and corneal involvement Here are some examples of grading systems: The Bonini et al. grading system: This system assigns scores based on symptoms and signs, with higher scores indicating greater severity. Importance of Early Diagnosis: Early diagnosis and treatment of VKC, regardless of severity, are crucial to prevent complications like corneal scarring, which can permanently affect vision. If you experience any symptoms suggestive of VKC, consulting an ophthalmologist is essential for proper diagnosis, assessment of severity, and development of a personalized treatment plan.
		Vernal keratoconjunctivitis (VKC) can manifest in a range of severity, from mild and seasonal to chronic and sight-threatening. Here's a breakdown of the spectrum of VKC severity: Grading Systems: There's no universally accepted grading system for VKC severity. However, ophthalmologists (eye doctors) often consider a combination of factors to assess the condition's intensity: Ocular Signs: This includes the presence, size, and location of papillae (bumps) on the inner eyelids, corneal involvement (inflammation or scarring), and overall redness and discharge. Symptoms: The severity of itching, tearing, light sensitivity, and foreign body sensation are all taken into account. Spectrum of Severity: Based on these factors, VKC can be broadly categorized into the following: Mild VKC: This is characterized by: Seasonal symptoms, often worse in spring and summer. Small papillae on the inner eyelids. Occasional itching, tearing, and redness. Minimal to no corneal involvement. Generally well-controlled with artificial tears and mast cell stabilizers. Moderate VKC: This involves: More prominent seasonal worsening of symptoms. Larger and more numerous papillae. Increased frequency and intensity of itching, tearing, and light sensitivity. Possible presence of punctate keratitis (tiny corneal infiltrates). May require additional medications like topical corticosteroids (short-term use). Severe VKC: This is characterized by: Chronic or year-round symptoms. Giant papillae covering a large portion of the inner eyelids. Significant discomfort with constant itching, tearing, and light sensitivity. Potential corneal involvement with ulcers or scarring. May require a combination of medications, including cyclosporine A, and close monitoring by the ophthalmologist. Increased risk of vision impairment if left untreated. Importance of Early Diagnosis: Regardless of the severity, early diagnosis and proper management of VKC are crucial. This can help prevent complications like corneal scarring, which can significantly affect vision. Additional Considerations: Individual Variability: The way VKC presents and progresses can vary significantly between individuals. Some may experience a rapid escalation of symptoms, while others might have a more gradual progression. Long-Term Management: Even in mild cases, VKC often requires ongoing management, particularly during allergy seasons, to prevent flare-ups. If you suspect you or your child has VKC, consult an ophthalmologist for a proper diagnosis and personalized treatment plan based on the specific severity of the condition.
18	What eye drops should I get for vernal keratoconjunctivitis?	I can't recommend specific medications, including eye drops, as it requires a medical diagnosis and professional guidance. Vernal keratoconjunctivitis (VKC) treatment depends on the severity of the condition and your child's individual needs. Here's why consulting an ophthalmologist is crucial: Accurate diagnosis: An ophthalmologist can confirm VKC diagnosis and rule out other conditions with similar symptoms. Severity assessment: They can assess the severity of your child's VKC to determine the most appropriate course of treatment. Personalized plan: Based on the severity and your child's specific situation, the ophthalmologist will create a personalized treatment plan that might include: Antihistamine eye drops: These help reduce itching, a hallmark symptom of VKC. Mast cell stabilizers: These eye drops work by preventing the release of inflammatory chemicals and are typically used for long-term prevention of flare-ups. Corticosteroids: In severe cases, ophthalmologists might prescribe short-term topical corticosteroids to manage inflammation. However, due to potential side effects, they are used cautiously and for a limited duration. Over-the-counter options: While I cannot recommend specific brands, over-the-counter lubricating eye drops might be helpful for temporary relief of dryness and irritation associated with VKC. However, it's important to consult an ophthalmologist before using any eye drops, including over-the-counter ones, to ensure they won't worsen your child's condition. Here's what you can do: Schedule an appointment: Make an appointment with an ophthalmologist who can examine your child, determine the appropriate course of treatment, and prescribe the most suitable eye drops for their specific needs. Discuss concerns: Don't hesitate to discuss any questions or concerns you have about medications, side effects, or alternative treatment options with the ophthalmologist. Remember, early diagnosis and proper management are crucial for effectively controlling VKC symptoms and preventing complications.
		I cannot recommend specific medications or eye drops for vernal keratoconjunctivitis (VKC) as it requires a proper diagnosis and treatment plan from a licensed ophthalmologist (eye doctor). Here's why: Diagnosis is crucial: VKC can mimic other eye conditions, and a doctor needs to confirm the diagnosis with an eye exam. Tailored treatment: The most appropriate eye drops depend on the severity of your child's VKC and their individual needs. General categories of eye drops used for VKC: Artificial tears: Lubricate dry and irritated eyes. Antihistamine eye drops: Reduce allergic reactions and inflammation. Mast cell stabilizers: Prevent the release of inflammatory substances. Corticosteroid eye drops (used cautiously): Reduce severe inflammation (used for short-term management or in severe cases). Importance of consulting an ophthalmologist: They can: Diagnose VKC and differentiate it from other conditions. Prescribe the most suitable eye drops based on your child's specific situation. Instruct on proper usage and potential side effects of the medication. Monitor your child's progress and adjust the treatment plan as needed. Here are some resources you may find helpful: American Academy of Ophthalmology: https://www.aao.org/education/disease-review/vernal-keratoconjunctivitis-5 National Eye Institute: https://www.nei.nih.gov/ Remember, early diagnosis and proper management from an ophthalmologist are essential for optimal eye health and preventing complications.
		I can't recommend specific eye drops for vernal keratoconjunctivitis (VKC) because it's crucial to consult a qualified healthcare professional, preferably an ophthalmologist (eye doctor), for proper diagnosis and treatment. Here's why: Diagnosis: An ophthalmologist can accurately diagnose VKC by performing a comprehensive eye examination and reviewing your medical history. They can rule out other conditions that might cause similar symptoms. Severity assessment: The appropriate type and strength of eye drops depend on the severity of your VKC. A doctor will assess the intensity and frequency of your symptoms and the presence of any ocular signs like papillae or corneal involvement. Individual needs: The best eye drops for you will depend on your specific needs and potential allergies to medications. Here are the general categories of eye drops commonly used for VKC: Artificial tears: These lubricating drops help soothe irritation and wash away allergens. Antihistamines: These drops block histamine, an inflammatory chemical, to reduce itching and redness. Mast cell stabilizers: These drops prevent the release of inflammatory substances from mast cells, offering long-term relief. Corticosteroids: These powerful anti-inflammatory drops are typically reserved for severe cases due to potential side effects with prolonged use. Here's what you can do: Schedule an appointment with an ophthalmologist. They can diagnose your condition, assess the severity, and prescribe the most appropriate eye drops for your specific case. Do not self-medicate. Using eye drops not prescribed by your doctor could worsen your condition or cause side effects. If you have any questions about VKC or eye drops, discuss them with your ophthalmologist. They can provide personalized guidance and ensure you receive safe and effective treatment for your VKC.
		I can't recommend specific medications or eye drops for vernal keratoconjunctivitis (VKC) as it requires a proper diagnosis and treatment plan from a licensed ophthalmologist. Here's why: Diagnosis is crucial: A qualified ophthalmologist can accurately diagnose VKC, assess the severity, and identify any potential underlying conditions. This is important for selecting the most appropriate treatment approach. Severity determines treatment: The type of eye drops prescribed depends on the severity of your VKC. For mild cases, artificial tears or over-the-counter antihistamine eye drops might suffice. In moderate or severe cases, stronger prescription medications like corticosteroids might be necessary. Potential side effects: Some eye drops, like corticosteroids, can have side effects with prolonged use. An ophthalmologist can weigh the risks and benefits and prescribe the safest and most effective option for you. Personalized plan: Your ophthalmologist will consider your individual needs and medical history when creating a treatment plan. This might involve a combination of medications, lifestyle modifications, and allergy management strategies. What you can do: Consult an ophthalmologist: This is the most important step. They can diagnose VKC, determine the severity, and recommend the most suitable eye drops and overall treatment plan for your specific case. Discuss over-the-counter options: You can discuss the possibility of using over-the-counter artificial tears or antihistamine eye drops for temporary relief with your ophthalmologist. They can advise you on the most appropriate options based on your situation. Here are some general categories of eye drops used for VKC: Artificial tears: Lubricate and soothe dry, irritated eyes. Available over-the-counter. Antihistamine eye drops: Reduce itching and inflammation caused by allergies. Can be over-the-counter or prescription depending on strength. Corticosteroid eye drops: Powerful anti-inflammatory medication reserved for severe cases or those unresponsive to other treatments. Use with caution due to potential side effects. Remember, consulting an ophthalmologist is key to getting the proper diagnosis, treatment plan, and most suitable eye drops for your VKC.
		I can't recommend specific eye drops for vernal keratoconjunctivitis (VKC) as prescription medication should only be given by a qualified healthcare professional. Here's why consulting an ophthalmologist (eye doctor) is crucial for determining the right eye drops for VKC: Diagnosis and Severity: An ophthalmologist can accurately diagnose VKC and assess its severity through an eye examination. This is essential for selecting the most appropriate medication. Personalized Treatment Plan: The best eye drops for VKC depend on the specific symptoms and severity. For instance, mild cases might benefit from lubricants like artificial tears, while moderate cases might require mast cell stabilizers or antihistamines. Potential Side Effects: Certain eye drops can have side effects, and the ophthalmologist can consider your individual medical history to choose medications with minimal risks. Monitoring Progress: Regular follow-up appointments with your ophthalmologist allow them to monitor your response to the medication and adjust the treatment plan as needed. Over-the-counter options: While I can't recommend specific medication, artificial tears are generally considered safe and can provide temporary relief from dryness and irritation associated with VKC. However, it's important to note that they won't address the underlying inflammatory response in VKC. Prescription options: Depending on the severity and your specific needs, your ophthalmologist might prescribe various eye drops, including: Artificial tears: Lubricate and soothe the eyes. Mast cell stabilizers: Prevent the release of inflammatory chemicals from mast cells in the eye, reducing itching and inflammation. Topical antihistamines: Target histamine, another key player in the allergic response, further reducing itching and redness. Corticosteroids: Potent anti-inflammatory medications used short-term for severe cases due to potential side effects with long-term use. Cyclosporine A: An immunosuppressant medication used in severe cases that don't respond well to other treatments. Remember: Consulting an ophthalmologist is vital for proper diagnosis, personalized treatment plan development, and safe and effective use of eye drops for managing VKC.
19	Can you get side effects from the eyedrops treating vernal keratoconjunctivitis?	Yes, like most medications, eye drops used to treat vernal keratoconjunctivitis (VKC) can have potential side effects. However, the risk and severity of these side effects vary depending on the type of medication used and the duration of use. Here's a breakdown of the most common eye drops used for VKC and their potential side effects: 1. Antihistamine Eye Drops: Function: Reduce itching, a hallmark symptom of VKC. Side Effects: Typically mild and may include: Burning sensation Stinging Dryness Blurred vision (usually temporary) 2. Mast Cell Stabilizers: Function: Prevent the release of inflammatory chemicals, used for long-term prevention of flare-ups. Side Effects: Generally well-tolerated, but may cause: Burning sensation Stinging Dryness Blurred vision (usually temporary) 3. Corticosteroids: Function: Powerful anti-inflammatory medication used for short-term management of severe VKC symptoms. Side Effects: Due to their potency, corticosteroids carry a higher risk of side effects, especially with prolonged use. These can include: Increased intraocular pressure (IOP) which can lead to glaucoma Cataract formation Thinning of the cornea Increased risk of infection Minimizing Side Effects: Ophthalmologist's Role: The ophthalmologist will prescribe the most appropriate medication based on your child's needs and ensure the lowest effective dose is used to minimize the risk of side effects. Following Instructions: It's crucial to adhere to the doctor's instructions regarding dosage, frequency, and duration of use to reduce the risk of complications. Reporting Concerns: If you notice any concerning side effects while using the eye drops, consult the ophthalmologist immediately. Additional Points: Alternatives: In some cases, depending on the severity and your child's specific situation, the ophthalmologist might explore alternative treatment options with potentially fewer side effects. Monitoring: During treatment, the ophthalmologist will monitor your child for any potential side effects, especially when using corticosteroids. Overall, while eye drops used for VKC can have side effects, the benefits of managing the symptoms often outweigh the risks. Consulting with an ophthalmologist and following their recommendations can help ensure your child receives the most effective treatment with the least risk of side effects.
		Yes, like most medications, eye drops used to treat vernal keratoconjunctivitis (VKC) can have potential side effects. However, the benefits of managing VKC symptoms typically outweigh the risks when used appropriately under the guidance of an ophthalmologist (eye doctor). Here's a breakdown of the most common types of eye drops used for VKC and their potential side effects: Artificial Tears: These are generally very safe with minimal side effects. In rare cases, some people might experience temporary stinging or burning upon application. Antihistamine Eye Drops: Potential side effects can include: Dryness, burning, or stinging sensation in the eyes. Blurred vision (usually temporary). Eyelid irritation. Mast Cell Stabilizers: Generally well-tolerated, but some people might experience: Burning or stinging sensation in the eyes. Blurred vision (usually temporary). Eyelid redness or irritation. It's important to note that mast cell stabilizers take time (weeks) to reach their full effect. Corticosteroid Eye Drops (Used Cautiously): Due to their potency, these are typically reserved for short-term management of severe inflammation or acute flare-ups. Potential side effects with prolonged use include: Increased intraocular pressure (eye pressure) which can lead to glaucoma. Cataract formation (clouding of the lens). Thinning of the cornea (the clear outer dome of the eye). Because of these potential complications, ophthalmologists carefully monitor patients using steroid eye drops. Minimizing side effects: Follow doctor's instructions: Use the eye drops exactly as prescribed by your ophthalmologist regarding dosage, frequency, and duration of use. Don't touch the tip of the dropper: This can contaminate the solution. Apply gentle pressure to the inner corner of the eye: This helps prevent medication from draining into the tear duct and potentially reaching the bloodstream. Wait a few minutes between applying different medications: If your child needs to use multiple types of eye drops, wait a few minutes between each one to allow for proper absorption. Importance of consulting an ophthalmologist: They can: Discuss the potential side effects of each medication in detail. Prescribe the most appropriate eye drops with the least risk of side effects based on your child's specific needs. Monitor your child for any potential side effects and adjust the treatment plan as needed. By understanding the potential side effects and using eye drops appropriately under an ophthalmologist's supervision, you can help your child manage their VKC symptoms effectively and minimize any potential risks.
		Yes, some of the eye drops used to treat vernal keratoconjunctivitis (VKC) can cause side effects. Here's a breakdown of the potential side effects associated with the common types of VKC eye drops: Artificial tears: These are generally very safe with minimal side effects. However, preserved artificial tears might cause temporary stinging or burning in some people. Opting for preservative-free artificial tears can minimize this discomfort. Antihistamines: While typically well-tolerated, some antihistamine eye drops might cause temporary burning, stinging, or a foreign body sensation in the eye. Mast cell stabilizers: These are usually well-tolerated, but some people might experience temporary burning or stinging upon instillation. They may take a few weeks of consistent use to show their full effect. Corticosteroids: Corticosteroid eye drops are powerful medications and should be used with caution and under an ophthalmologist's supervision due to potential side effects. These can include: Increased intraocular pressure (IOP), which can lead to glaucoma, a serious eye condition that can damage the optic nerve and vision. Cataract formation, a clouding of the lens of the eye that can impair vision. Thinning of the cornea, the clear dome at the front of the eye, which can weaken it and lead to vision problems. Here are some general tips to minimize the risk of side effects from eye drops: Follow your doctor's instructions: Use the eye drops exactly as prescribed by your ophthalmologist regarding dosage, frequency, and duration of use. Don't touch the tip of the dropper: This can contaminate the solution with bacteria. Wash your hands before using the drops: This helps prevent infection. Avoid contact between the dropper tip and your eye: Tilt your head back and gently pull down your lower eyelid to create a pocket. Instill the drops into the pocket without touching your eye with the dropper tip. Close your eye for a minute: Apply gentle pressure to the inner corner of your eye for a minute after instilling the drops to help them penetrate the eye and reduce drainage. Wait five minutes between medications: If you're using multiple types of eye drops, wait at least five minutes between applying them to allow each medication time to be absorbed. Report any side effects to your doctor: If you experience any side effects, such as worsening symptoms, burning, or stinging that persists, discuss them with your ophthalmologist. They can advise you on whether to adjust the dosage, switch medications, or discontinue use. Remember: The benefits of using prescribed eye drops typically outweigh the risks when used appropriately under your ophthalmologist's supervision. They will carefully consider the severity of your VKC and potential side effects when choosing the most suitable eye drops for you.
		Yes, some of the eye drops used to treat vernal keratoconjunctivitis (VKC) can cause side effects. Here's a breakdown of the potential risks associated with different types of eye drops: Artificial Tears: While generally safe, some artificial tears, especially those containing preservatives, might cause temporary stinging or burning upon application. Opting for preservative-free artificial tears can minimize this discomfort. Antihistamine Eye Drops: Common side effects can include: Dryness or burning sensation in the eyes Blurred vision (usually temporary) Stinging upon application Corticosteroid Eye Drops: These medications are powerful and effective for severe VKC but come with more potential side effects, especially with prolonged use. These can include: Increased intraocular pressure (IOP) which can lead to glaucoma, a serious eye condition that can damage the optic nerve and vision Cataract formation (clouding of the lens of the eye) Thinning of the cornea (the clear front surface of the eye) Increased risk of infections Here are some general tips to minimize side effects from eye drops: Follow your doctor's instructions: Use the eye drops exactly as prescribed by your ophthalmologist in terms of dosage, frequency, and duration of use. Don't touch the tip of the dropper: This can contaminate the solution and potentially introduce bacteria into the eye. Wash your hands: Always wash your hands thoroughly with soap and water before and after instilling eye drops. Avoid sharing eye drops: Sharing eye drops can spread infections. Report side effects to your doctor: If you experience any side effects, even mild ones, inform your ophthalmologist so they can adjust your treatment plan if necessary. Remember: The potential benefits of eye drops for managing VKC symptoms typically outweigh the risks, especially when used appropriately under the guidance of an ophthalmologist. Your doctor will carefully consider the severity of your VKC, your individual risk factors, and the potential side effects before prescribing any medication. If you have any concerns about the side effects of eye drops used for VKC, discuss them with your ophthalmologist. They can address your specific questions and ensure you receive the safest and most effective treatment for your condition.
		Yes, some of the eye drops used to treat vernal keratoconjunctivitis (VKC) can cause side effects. Here's a breakdown of the potential side effects associated with different types of eye drops commonly prescribed for VKC: Artificial Tears: Generally well-tolerated with minimal side effects. In rare cases, some individuals might experience temporary stinging or burning upon instillation. Mast Cell Stabilizers: Usually well-tolerated, but some people might experience: Blurred vision (usually temporary). Eye irritation (burning, stinging). A dry, gritty sensation in the eyes. Topical Antihistamines: Generally well-tolerated, but potential side effects include: Blurred vision (usually temporary). Eye irritation (burning, stinging). Dryness in the eyes. Corticosteroids: Potent medications with more significant side effects, especially with prolonged use. These can include: Increased intraocular pressure (IOP) which can lead to glaucoma. Cataract formation (clouding of the lens). Thinning of the cornea (the clear dome at the front of the eye). Increased risk of infections Cyclosporine A: An immunosuppressant medication with potential side effects like: Eye burning or stinging. Increased risk of infections. Elevated blood pressure. Kidney problems (rare). Importance of Doctor Monitoring: To minimize the risk of side effects, it's crucial to use eye drops exactly as prescribed by your ophthalmologist (eye doctor) and attend regular follow-up appointments. They can monitor for potential side effects and adjust the medication or dosage as needed. Knowing the Risks: Before starting any medication, discuss the potential side effects with your ophthalmologist and weigh the benefits against the risks. Alternative Options: In some cases, your ophthalmologist might explore alternative options like cold compresses or allergen avoidance strategies, especially for mild VKC, to minimize reliance on medications with potential side effects. Overall, while some eye drops used for VKC can cause side effects, the benefits of managing the condition often outweigh the risks. Consulting an ophthalmologist and following their guidance is essential for safe and effective use of these medications.
20	Are the eye drops used in vernal keratoconjunctivitis dangerous?	Eye drops used to treat vernal keratoconjunctivitis (VKC) aren't necessarily dangerous, but like most medications, they can have side effects. Here's a breakdown of the risks and benefits to consider: Benefits: Symptom control: Effective eye drops can significantly reduce itching, tearing, light sensitivity, and other uncomfortable symptoms of VKC, improving your child's quality of life. Preventing complications: Properly managed VKC can help prevent complications like corneal scarring, which can potentially impair vision. Risks: Side effects: As discussed earlier, each type of eye drop used for VKC has potential side effects, ranging from mild discomfort to more serious complications with prolonged use, particularly with corticosteroids. Weighing the risks and benefits: In most cases, the benefits of managing VKC symptoms with eye drops outweigh the risks, especially when used under the supervision of an ophthalmologist. Ophthalmologists will prescribe the most appropriate medication at the lowest effective dose to minimize side effects. Here's how to minimize risks: Ophthalmologist's guidance: Consulting an ophthalmologist is crucial. They can: Diagnose VKC accurately. Assess the severity of your child's condition. Prescribe the most suitable medication based on your child's needs. Monitor your child for any potential side effects. Following instructions: It's essential to strictly adhere to the ophthalmologist's instructions regarding dosage, frequency, and duration of use. Communicating concerns: If you notice any concerning side effects while using the eye drops, consult the ophthalmologist immediately. Remember: Not all eye drops are created equal: Different types of eye drops carry varying degrees of risk. Short-term use is generally safer: The risk of side effects increases with prolonged use. Alternatives exist: In some cases, the ophthalmologist might explore alternative treatment options with potentially fewer side effects. Overall, eye drops used for VKC can be a safe and effective way to manage symptoms and improve your child's quality of life when used appropriately under the guidance of an ophthalmologist. Don't hesitate to discuss any concerns you have about eye drops or their side effects with your child's ophthalmologist. They can provide the most up-to-date and personalized advice based on your child's specific situation.
		The eye drops used for vernal keratoconjunctivitis (VKC) aren't inherently dangerous when used appropriately under the guidance of an ophthalmologist (eye doctor). Here's a breakdown of the risks and benefits: Benefits: Symptom management: Eye drops can effectively control symptoms like itching, redness, and tearing, improving your child's comfort and quality of life. Preventing complications: By managing inflammation, eye drops can help prevent complications like corneal ulcers or scarring, which can potentially impair vision. Risks (mostly associated with corticosteroids): Side effects: As discussed previously, some eye drops, particularly corticosteroids, can have side effects like: Increased eye pressure (glaucoma) Cataract formation (clouding of the lens) Corneal thinning Minimizing risks: Ophthalmologist supervision: An ophthalmologist can: Choose the most appropriate eye drops with the lowest risk of side effects based on your child's VKC severity. Prescribe the correct dosage and duration of treatment to minimize risks. Monitor your child for any potential side effects and adjust the treatment plan as needed. Overall: The benefits of managing VKC symptoms with eye drops typically outweigh the risks when used appropriately. Ophthalmologists are aware of the potential side effects and take steps to minimize them. Here are some additional points to consider: Alternatives to corticosteroids: For milder cases, ophthalmologists might prioritize non-steroidal options like artificial tears, antihistamines, or mast cell stabilizers before resorting to corticosteroids. Importance of communication: Discuss any concerns you have about side effects with your ophthalmologist. They can address your questions and explain the risks and benefits in detail. Remember, early diagnosis and proper management from an ophthalmologist are crucial for optimal eye health and preventing complications associated with VKC.
		Eye drops used for vernal keratoconjunctivitis (VKC) aren't inherently dangerous when used as directed by an ophthalmologist (eye doctor). Here's a breakdown of the risks and benefits to consider: Benefits: Effective relief: Most VKC eye drops are very effective in managing symptoms like itching, redness, and watering, improving your quality of life. Reduced risk of complications: Early diagnosis and proper treatment with eye drops can help prevent complications like corneal scarring, which can affect vision. Variety of options: Depending on the severity of your VKC, there are different categories of eye drops with varying strengths to target your specific needs. Risks: Side effects: As discussed earlier, some eye drops can cause temporary side effects like burning, stinging, or a foreign body sensation. Corticosteroid drops, while powerful, require careful monitoring due to potential complications like glaucoma or cataracts with prolonged use. Misuse: Using eye drops not prescribed by your doctor or using them incorrectly can worsen your VKC or cause other eye problems. Here's how to minimize risks and maximize benefits: Consult an ophthalmologist: They can diagnose your VKC, assess the severity, and prescribe the most appropriate eye drops for your specific case. They will consider your individual needs and potential allergies to medications. Follow instructions carefully: Use the eye drops exactly as prescribed regarding dosage, frequency, and duration. Report side effects: If you experience any bothersome side effects, discuss them with your ophthalmologist. They can adjust the dosage, switch medications, or discontinue use if necessary. Schedule regular eye checkups: Maintain regular appointments with your ophthalmologist to monitor the response to treatment and check for any potential side effects. Overall, the benefits of using prescribed eye drops for VKC outweigh the risks when used appropriately under your doctor's supervision. They are a safe and effective way to manage VKC symptoms and improve your quality of life. If you have any concerns about the safety of eye drops, discuss them openly with your ophthalmologist.
		The eye drops used to treat vernal keratoconjunctivitis (VKC) aren't inherently dangerous, but like most medications, they can have side effects, especially with prolonged use. Here's a breakdown of the risks and benefits to consider: Types of Eye Drops and Potential Side Effects: Artificial Tears: Generally safe. Some might cause temporary stinging or burning, especially those with preservatives. Preservative-free options can minimize this. Antihistamine Eye Drops: Common side effects include dryness, burning, blurred vision (temporary), and stinging upon application. Corticosteroid Eye Drops: Potent and effective for severe VKC, but come with more potential side effects, particularly with long-term use: Increased eye pressure (glaucoma risk) Cataract formation (clouding of the lens) Corneal thinning Increased infection risk Balancing Risks and Benefits: The benefits of managing VKC symptoms with eye drops typically outweigh the risks, especially when used appropriately under an ophthalmologist's guidance. Your doctor will consider your VKC severity, individual risk factors, and potential side effects before prescribing anything. Minimizing Side Effects: Follow doctor's instructions: Use the eye drops exactly as prescribed regarding dosage, frequency, and duration. Proper hygiene: Wash hands thoroughly before and after instilling drops. Don't touch the dropper tip. Avoid sharing eye drops: This can spread infections. Report side effects: Inform your doctor about any side effects, even mild ones, so they can adjust treatment if needed. When to be Concerned: While uncommon, serious side effects from VKC eye drops are possible. If you experience: Severe eye pain Significant vision changes Signs of infection (redness, pus discharge) Seek immediate medical attention from your ophthalmologist. Conclusion: Eye drops for VKC are valuable tools to manage symptoms and improve quality of life. Understanding the potential side effects allows you to be informed and discuss any concerns with your doctor. They can ensure you receive the safest and most effective treatment plan for your specific case.
		The eye drops used in vernal keratoconjunctivitis (VKC) aren't necessarily dangerous, but they can cause side effects, especially with certain medications or improper use. Here's a breakdown of the risks and how to manage them: Safe and Effective Use: When used correctly under the supervision of an ophthalmologist (eye doctor), most eye drops for VKC are safe and effective in managing symptoms and preventing complications. The ophthalmologist will consider factors like the severity of VKC, your individual needs, and medical history to prescribe the most appropriate medication with minimal risk of side effects. Potential Side Effects: Different types of eye drops used for VKC can cause varying side effects, as discussed previously. These can range from mild and temporary like blurred vision or stinging to more serious ones like glaucoma or cataracts with prolonged steroid use. Minimizing Risks: Following Doctor's Instructions: Using eye drops exactly as prescribed by your ophthalmologist is crucial. This includes adhering to the dosage, frequency, and duration of treatment. Regular Follow-Up Appointments: Attending scheduled checkups with your ophthalmologist allows them to monitor for potential side effects and adjust the treatment plan as needed. Communicating Issues: If you experience any side effects, talk to your ophthalmologist immediately. They can advise on managing the side effects or recommend alternative medications. Remember: The benefits of treating VKC with eye drops often outweigh the risks, especially when managed by a qualified healthcare professional. Properly controlled VKC can significantly improve your quality of life and prevent vision problems. Here's a comparison for better understanding: Benefits: Reduced itching, redness, and tearing. Improved comfort and quality of life. Prevented complications like corneal ulcers and scarring. Risks (with proper use): Usually mild and temporary side effects like burning or blurred vision. Less common but serious side effects like glaucoma or cataracts with prolonged steroid use (these are closely monitored by the ophthalmologist). In conclusion, eye drops for VKC are valuable tools for managing the condition. By following your ophthalmologist's guidance and being aware of potential side effects, you can ensure safe and effective treatment for VKC.
21	My kid has vernal keratoconjunctivitis. Should I get him tested for allergies?	While getting your child tested for allergies might be helpful in managing their vernal keratoconjunctivitis (VKC), it's not necessarily mandatory for everyone with VKC. Here's a breakdown of the reasons why allergy testing might be considered and some additional factors to consider: Possible benefits of allergy testing: Identifying triggers: Knowing what allergens your child is sensitive to can help you take steps to avoid them, potentially reducing the frequency and severity of VKC flare-ups. Common triggers for VKC allergies include pollen, dust mites, pet dander, and mold. Targeted treatment: If specific allergies are identified, the ophthalmologist might tailor your child's treatment plan accordingly. For example, they might recommend allergen avoidance strategies or prescribe allergy medications specific to the identified triggers. However, it's important to understand: Not everyone with VKC has allergies: While VKC is considered an allergic eye disease, not all cases involve a clear allergic trigger. Up to 50% of VKC patients might test negative for allergies. Test limitations: Allergy tests aren't perfect. They can sometimes produce false positive or negative results. Consulting your child's ophthalmologist is crucial to determine if allergy testing is necessary or beneficial in your child's specific case. Here are some factors they might consider: Severity of VKC: Testing might be more likely to be recommended for children with severe VKC or those who don't respond well to initial treatment approaches. Suspected allergens: If you have a strong suspicion about potential allergens based on your child's environment or activities, testing might be helpful for confirmation. Overall health: If your child has a history of other allergies (e.g., eczema, asthma), allergy testing might be more likely to be recommended. Here's what you can do: Discuss with ophthalmologist: Talk to your child's ophthalmologist about the pros and cons of allergy testing in your child's specific situation. Ask questions: Don't hesitate to ask questions about the types of allergy tests available, their accuracy, and how the results might influence your child's treatment plan. Remember: Even if your child tests negative for allergies, there are several strategies to manage VKC effectively. Focus on working with your child's ophthalmologist to develop a personalized treatment plan that addresses their specific needs.
		While allergy testing isn't mandatory for diagnosing vernal keratoconjunctivitis (VKC) in your child, it can be helpful in some cases. Here's a breakdown of the situation: Diagnosis of VKC: Ophthalmologists can diagnose VKC based on your child's symptoms, medical history, and a thorough eye exam. This typically involves examining the eyelids and cornea for signs of inflammation and papillae (small bumps). Role of allergy testing: Not always necessary: Up to 50% of VKC cases might not show positive allergy test results, even though it's considered an allergic condition. Can be informative: However, allergy testing can identify specific allergens your child might be sensitive to, like pollen, dust mites, or pet dander. This information can be helpful for: Avoiding triggers: If possible, minimizing exposure to identified allergens can help reduce the frequency and severity of VKC flare-ups. Targeted treatment: In some cases, allergy testing can guide the ophthalmologist towards specific medications or immunotherapy for managing allergies, potentially reducing reliance solely on eye drops for VKC. Consulting your ophthalmologist: Discuss the pros and cons of allergy testing with your child's ophthalmologist. They can consider factors like: Severity of your child's VKC. Presence of other atopic conditions like asthma or eczema (which can suggest a higher likelihood of allergies playing a role in VKC). Your concerns and interest in identifying potential triggers. Here are some additional points to consider: Types of allergy testing: Skin prick tests or blood tests can be used to identify allergies. Limitations of allergy testing: Even with positive test results, it's not always clear how significantly those allergies contribute to VKC symptoms. Ultimately, the decision of whether or not to allergy test your child depends on your specific situation and your ophthalmologist's recommendations. By working together, you can create the best management plan to improve your child's comfort and eye health.
		It can be very helpful to get your child tested for allergies if they have vernal keratoconjunctivitis (VKC). Here's why: Strong association: While VKC isn't strictly an allergic reaction, there's a strong link between allergies and VKC. Children with a family history of allergies (eczema, asthma) or who have allergies themselves are more prone to developing VKC. Identifying triggers: Allergy testing can help identify specific allergens that might be triggering VKC flare-ups in your child. Common triggers include pollen, dust mites, and pet dander. Knowing the triggers can help you take steps to avoid them and potentially reduce the severity of symptoms. Management strategy: Even if allergy testing doesn't reveal specific allergens, it can still be a valuable tool for your child's ophthalmologist (eye doctor) when developing a management strategy for VKC. Here are the different types of allergy tests that might be used: Skin prick test: This is a common test where a small amount of potential allergens is pricked onto the skin and observed for a reaction (wheal and redness). Blood test: This test measures the amount of specific antibodies (IgE) in the blood that react to certain allergens. Here's what to discuss with your child's ophthalmologist: Symptoms: Describe your child's VKC symptoms in detail, including the frequency and severity of itching, redness, tearing, and any other discomfort. Family history: Mention any family history of allergies in your child's relatives. Benefits and risks of allergy testing: Discuss the potential benefits of allergy testing in helping manage your child's VKC and any potential risks or discomforts associated with the tests. Ultimately, the decision of whether to allergy test your child rests with you and your child's ophthalmologist. They can assess your child's specific situation and recommend the best course of action. Here are some additional points to consider: Allergy testing is generally safe and well-tolerated in children. Even if allergy testing doesn't reveal specific triggers, it can still be informative for your child's VKC management. Avoiding potential allergens, regardless of a positive allergy test, can often help reduce VKC symptoms. Remember, I am not a medical professional and cannot provide medical advice. Consulting your child's ophthalmologist is crucial for proper diagnosis, treatment, and determining if allergy testing is right for your child's case.
		While allergy testing isn't always mandatory for diagnosing vernal keratoconjunctivitis (VKC) in children, it can be helpful in some cases. Here's a breakdown of the pros and cons to consider: Possible benefits of allergy testing: Identify triggers: If your child has other allergies or atopic conditions like eczema or asthma, allergy testing can help identify specific allergens that might worsen VKC symptoms. This allows you to take steps to avoid those triggers and potentially reduce the severity of flare-ups. Targeted treatment: Knowing the specific allergens can help your doctor tailor a more targeted treatment plan. For example, if pollen is a trigger, they might recommend using allergy eye drops during peak pollen seasons. Management of other allergies: If your child has other allergies, identifying the triggers can help with overall allergy management, improving their quality of life. Points to consider: Not always conclusive: VKC can occur even without a positive allergy test. Up to 50% of VKC cases might not show positive results on allergy tests. Test limitations: Allergy tests may not always be completely accurate, and some allergens might not be detected. Cost and discomfort: Allergy testing can involve skin prick tests or blood tests, which can be slightly uncomfortable for your child and come with additional costs. Discussing allergy testing with your doctor: The decision to get your child tested for allergies depends on several factors, including: Severity of VKC: For severe VKC cases, identifying potential triggers can be very helpful. Presence of other allergies: If your child has other allergies or atopic conditions, testing can be beneficial for overall management. Your child's age and tolerance: Consider your child's age and ability to tolerate the testing procedures. Here's what you can do: Talk to your child's ophthalmologist about the pros and cons of allergy testing in your child's specific situation. Discuss your concerns and questions regarding VKC and potential allergy triggers. Follow your doctor's recommendations on whether allergy testing is necessary for your child. Remember, early diagnosis and proper management of VKC are crucial. Even if allergy testing isn't performed, there are still effective treatment options available to manage your child's VKC symptoms and improve their quality of life.
		Whether or not to get your child tested for allergies if they have vernal keratoconjunctivitis (VKC) depends on several factors. Here's a breakdown to help you decide: Potential Benefits of Allergy Testing: Identifying Triggers: Allergy testing can help identify specific allergens (like pollen, dust mites, pet dander) that might be triggering your child's VKC symptoms. This allows for: Targeted Avoidance: Once you know the triggers, you can implement strategies to minimize your child's exposure, potentially reducing the frequency and severity of VKC flare-ups. More Effective Treatment: Understanding the underlying allergies can guide the ophthalmologist (eye doctor) towards a more personalized treatment plan that addresses both VKC and the allergies. Ruling Out Other Allergic Conditions: Allergy testing might reveal allergies to other substances, such as food, which could be helpful for managing their overall health. Considerations for Testing: Not Always Necessary: While VKC is often associated with allergies, not all cases are allergy-driven. Your child's ophthalmologist can assess the need for allergy testing based on the severity and presentation of their VKC. Test Accuracy: Allergy tests aren't always perfect and can sometimes produce false positives or negatives. Cost and Time: Allergy testing can be expensive and time-consuming, especially if multiple tests are needed. Discussing with Ophthalmologist: The best course of action is to discuss the need for allergy testing with your child's ophthalmologist. They can consider factors like: Severity of your child's VKC. Presence of other allergy symptoms (like runny nose, sneezing). Family history of allergies. Potential benefits and drawbacks of testing in your child's specific case. Here are some general recommendations: Severe VKC: If your child has severe VKC or their symptoms are not adequately controlled with medications, allergy testing might be more likely to be recommended. Other Allergic Symptoms: If your child experiences other symptoms suggestive of allergies, such as hay fever or eczema, allergy testing might be beneficial for overall allergy management. Family History: A strong family history of allergies can also increase the likelihood of allergy testing being recommended. Ultimately, the decision of whether or not to get your child tested for allergies rests with you and your child's ophthalmologist. By considering the potential benefits and drawbacks in your child's specific situation, you can make an informed choice.
22	Will it help my vernal keratoconjunctivitis to get allergy vaccinated?	No, allergy vaccinations, also known as immunotherapy, are not a standard treatment for vernal keratoconjunctivitis (VKC) at this time. Here's why: VKC cause: VKC is an allergic reaction, but the exact cause is complex and may not involve just IgE antibodies, which is the target of allergy vaccinations. Limited research: While some studies on medications like omalizumab (targets IgE) show promise for VKC, these are not traditional allergy vaccinations and require further research. Currently, doctors focus on managing VKC symptoms with: Allergen avoidance: Identifying and avoiding triggers like pollen or dust mites. Eyedrops: Antihistamines or mast cell stabilizers to reduce inflammation and itching. Steroids: In severe cases, but for limited periods due to potential side effects. If you're concerned about VKC, it's best to consult an ophthalmologist (eye doctor) for proper diagnosis and a personalized treatment plan.
		Allergen immunotherapy, also known as allergy shots, might be helpful in managing your vernal keratoconjunctivitis (VKC). While there isn't a specific vaccine for VKC itself, allergy shots can target the underlying allergies that may be triggering your symptoms. Here's some information to consider: Effectiveness: Studies suggest allergen immunotherapy can be safe and effective for VKC (PubMed: Effectiveness of allergen immunotherapy in patients with vernal keratoconjuctivitis). It can help reduce symptoms and medication use. Discussion with your doctor: It's important to talk to your doctor about whether allergy shots are right for you. They can assess your specific case and determine if this approach aligns with your treatment plan. Here are some additional points to keep in mind: Allergy shots typically involve a series of injections over several months. They won't completely cure allergies, but can significantly reduce symptoms. If you're considering allergy shots, your doctor can discuss the potential benefits and risks in more detail.
		Allergy shots, also known as allergen immunotherapy, might be helpful for your vernal keratoconjunctivitis (VKC). While there isn't a specific vaccine for VKC itself, allergy shots can target the underlying allergies that trigger your symptoms. Here's some information: Studies have shown that allergy shots can be safe and effective for VKC (Effectiveness of allergen immunotherapy in patients with vernal keratoconjuctivitis). They can help reduce symptoms and medication use. However, it's important to note that allergy shots are a long-term treatment, typically taken over several months. They won't completely cure VKC, but can help manage it. Here's what I recommend: Talk to your doctor about allergy shots as a treatment option for your VKC. They can assess your individual situation and determine if it's the right approach for you. They can also discuss other treatment options for VKC, such as antihistamines, steroids, or immunosuppressants. I hope this information is helpful!
		Allergen immunotherapy, which can involve allergy shots, might be helpful for vernal keratoconjunctivitis (VKC) but it's not a guaranteed solution. Here's the breakdown: Possible benefit: Studies have shown some success with allergy immunotherapy in reducing symptoms and medication use for VKC patients (PubMed: Effectiveness of allergen immunotherapy in patients with vernal keratoconjunctivitis). Not a cure: Allergy shots aim to build tolerance to specific allergens, but they wouldn't eliminate VKC entirely, especially if the cause isn't well-identified. Here's what to consider: Talk to your doctor: They can assess your specific case and determine if allergy immunotherapy is a good option for you. Traditional treatments might be used first: Eye drops, medications, and cold compresses are common first-line treatments for VKC. If you're interested in learning more about allergy immunotherapy for VKC, consult your eye doctor. They can give you personalized advice based on your medical history.
		Allergy shots, also known as allergen immunotherapy, might be helpful for your vernal keratoconjunctivitis (VKC). There's research suggesting it can be safe and effective (Effectiveness of allergen immunotherapy in patients with vernal keratoconjunctivitis). However, it's important to talk to an ophthalmologist (eye doctor) to discuss if allergy shots are the right course of treatment for you. They can consider the severity of your VKC, what allergens you might be sensitive to, and your overall health. Here's a summary: Allergy shots hold promise for VKC treatment. Consult an ophthalmologist to discuss if it's suitable for you.
23	Why does my child have vernal keratoconjunctivitis when no allergy was found?	That's a good question. Vernal keratoconjunctivitis (VKC) is indeed considered an allergic condition, but it can be tricky because: Complex Mechanism: Unlike typical allergies, VKC's allergic response might not involve the usual IgE antibodies targeted by allergy tests. Other immune system components may be at play. Test Limitations: Standard allergy tests might not always pick up on the specific triggers for VKC. Here's a breakdown: Not Always IgE-Mediated: While some VKC cases show a link to allergens like pollen, up to 50% of patients test negative for allergies. This suggests other factors might be involved, like irritants, dry eyes, or even a genetic predisposition. Alternative Triggers: Even without a traditional allergy identified, there could be triggers causing an inflammatory response in your child's eyes. These might include dust mites, smoke, strong air fresheners, or even swimming pool chemicals. Here's what you can do: Consult the Ophthalmologist: Discuss your concerns about the negative allergy test results with your child's ophthalmologist. They can explore alternative triggers and discuss management strategies. Observe and Eliminate: Keep a log of your child's symptoms and see if there are any patterns related to activities or environments. This can help identify potential triggers for further avoidance. Focus on Management: The mainstay of VKC treatment involves managing symptoms with eyedrops (antihistamines or mast cell stabilizers) and possibly short-term steroids in severe cases. Remember, even though allergy tests might not pinpoint the exact cause, managing symptoms and identifying potential triggers can significantly improve your child's comfort.
		There are a couple reasons why your child might have vernal keratoconjunctivitis (VKC) even though allergy tests came back negative: Non-IgE Mediated Reactions: VKC is traditionally considered an allergic reaction, but research suggests other mechanisms might be involved. In up to 50% of VKC cases, allergy tests don't identify a specific allergen (PubMed: Understanding Vernal Keratoconjunctivitis: Beyond Allergic Mechanisms). This suggests other factors besides IgE-mediated allergies may be playing a role in the inflammation. Environmental Triggers: Dust mites, pollen, and smoke are all potential triggers for VKC, even if standard allergy testing doesn't pinpoint them. These irritants can cause an inflammatory response in some people, leading to VKC symptoms. Here are some additional things to consider: Genetics: There's a possibility of a genetic predisposition to VKC. If other family members have allergies or atopic conditions (eczema, asthma), your child might be more susceptible. Other Triggers: While less common, dry eyes, ultraviolet light exposure, and even contact lens use can trigger VKC in some individuals. It's important to talk to your child's ophthalmologist about the test results and their specific case. They can consider all the information and create a treatment plan to manage your child's VKC.
		That's a good question. Vernal keratoconjunctivitis (VKC) is interesting because even though it shares many features with allergic reactions, it doesn't always show up on allergy tests. Here's why your child might have VKC without a detectable allergy: Non-IgE Mechanism: VKC is traditionally considered an allergic condition. However, recent research suggests a more complex mechanism that doesn't solely rely on IgE antibodies, which are typically measured in allergy tests (Understanding Vernal Keratoconjunctivitis: Beyond Allergic Mechanisms). Other Triggers: While allergens can worsen VKC symptoms, other factors like dry eyes, dust mites, or even emotional stress can also be triggers (Vernal Keratoconjunctivitis - EyeWiki). Here are some things to consider: Discuss with your doctor: They can review your child's symptoms and perform a thorough eye exam to confirm VKC diagnosis. Non-allergic triggers: Talk to your doctor about possible non-allergic triggers in your child's environment. Management options: Even without a specific allergy identified, there are still effective treatments for VKC like artificial tears, antihistamine eye drops, or corticosteroids (Vernal keratoconjunctivitis: Current immunological and clinical evidence and the potential role of omalizumab). Remember, I am not a medical professional and this shouldn't replace consulting your child's doctor. They can provide the most accurate diagnosis and personalized treatment plan.
		Vernal keratoconjunctivitis (VKC) can be a bit of a puzzle. Even though it's often considered an allergic eye disease, allergy tests sometimes come back negative. Here are a few reasons why your child might have VKC despite no identified allergy: Non-IgE mediated inflammation: Traditional allergies involve immunoglobulin E (IgE), but VKC might involve other inflammatory pathways not detected by allergy testing (National Institutes of Health (.gov): Understanding Vernal Keratoconjunctivitis: Beyond Allergic Mechanisms). Environmental triggers: Dust, smoke, and even strong sunlight can irritate the eyes and trigger VKC symptoms even without a classic allergic response (News-Medical: What is Vernal Keratoconjunctivitis?). Genetics: There's a suspected link between VKC and family history of allergies or atopic conditions like eczema (National Center for Biotechnology Information: Management of vernal keratoconjunctivitis in children in Saudi Arabia). Here's what you can do: Talk to your child's ophthalmologist: They can discuss the possibility of non-allergic triggers and recommend strategies to manage symptoms. Identify potential irritants: Consider if your child is exposed to dust mites, smoke, strong sunlight, or other potential irritants at home, school, or outdoors. Focus on symptom relief: Treatments like eye drops, cold compresses, and avoiding triggers can help manage VKC even if the exact cause remains elusive. Remember, many children outgrow VKC as they get older.
		Vernal keratoconjunctivitis (VKC) is a tricky condition because even though it shares many characteristics of allergies, it isn't always triggered by a classic allergic reaction. Here's why your child might have VKC despite negative allergy tests: Non-IgE Mediated Response: VKC is traditionally thought of as an allergic reaction, but some studies suggest other mechanisms might be involved. The immune system can be overly sensitive without involving immunoglobulin E (IgE), a key player in allergies (Understanding Vernal Keratoconjunctivitis: Beyond Allergic Mechanisms). Environmental Irritants: Dust mites, pollen, and other allergens can worsen VKC symptoms, but they might not be the root cause. Exposure to smoke, strong wind, or irritants like chlorine in pools can also trigger flare-ups (What is Vernal Keratoconjunctivitis?). Here are some next steps: Talk to the Ophthalmologist: Discuss your child's negative allergy tests and explore alternative triggers. The doctor might recommend observing potential environmental triggers or keeping a symptom journal. Non-Allergy Treatments: Even without a confirmed allergy, treatments like mast cell stabilizers or corticosteroids can be very effective in managing VKC symptoms (Management of vernal keratoconjunctivitis in children in Saudi Arabia). Remember, I am not a medical professional. For any diagnosis or treatment plan, consult your child's ophthalmologist.
24	Can my kid have a pet if he has vernal keratoconjunctivitis?	Owning a pet with vernal keratoconjunctivitis (VKC) can be a possibility for your child, but there are some factors to consider: Pet Allergies: Testing Limitations: As discussed earlier, VKC might not show up in standard allergy tests. However, allergies to pet dander (dead skin flakes) are a concern. Consider consulting an allergist for a more specialized pet allergy test. Management Strategies: Hypoallergenic Breeds: If a pet allergy is confirmed, look into hypoallergenic breeds that shed less fur or dander, like poodles or certain types of terriers. Strict Hygiene: Regular pet grooming, frequent washing of bedding, and keeping pets off furniture can significantly reduce dander exposure. Continued VKC Management: Focus on managing your child's VKC symptoms with prescribed medications and avoid identified triggers (including potential pet dander). Trial and Observation: Start with Short Visits: If a specific pet is desired, consider supervised visits at a friend's house to see if your child experiences any worsening of VKC symptoms. Consulting the Doctor: Open Communication: Discuss your desire to get a pet with your child's ophthalmologist. They can assess the severity of VKC and offer personalized advice based on your child's specific situation. Remember: Every child and their VKC case is unique. While pets can be wonderful companions, prioritize your child's well-being and avoid introducing a pet if it significantly worsens their VKC symptoms.
		Having vernal keratoconjunctivitis (VKC) doesn't necessarily mean your child can't have a pet. However, there are some factors to consider: Triggers: Animal Dander: Pets like cats, dogs, and rabbits can shed dander (tiny skin flakes) that can trigger allergies and potentially worsen VKC symptoms. Management Strategies: Hypoallergenic Breeds: If you're set on a furry friend, consider hypoallergenic breeds that shed less dander, like poodles, bichon frises, or certain types of terriers. Minimize Exposure: Regular grooming, keeping pets out of bedrooms, and using air purifiers can help reduce dander exposure. Talk to your doctor: Discuss your child's specific situation and allergy triggers with their ophthalmologist. They can advise on potential risks and recommend ways to manage your child's VKC alongside a pet. Alternatives: Non-furry Pets: If dander is a major concern, consider fish, reptiles, or birds. These generally pose a lower allergy risk. Ultimately, the decision depends on your child's individual case and the severity of their VKC. It's best to discuss it with your doctor to weigh the potential benefits of pet ownership against the risk of triggering VKC symptoms.
		Having a pet with vernal keratoconjunctivitis (VKC) can be a bit tricky, but it's not necessarily impossible. Here's a breakdown of what to consider: Potential Issues: Triggers: Pets like cats and dogs can introduce allergens like dander, fur, and saliva into your child's environment. These allergens might worsen VKC symptoms like itching, redness, and watering eyes. Making it Work: Consult your doctor: Discuss your desire to get a pet with your child's doctor. They can assess the severity of your child's VKC and advise on suitable pet options. Hypoallergenic pets: Consider hypoallergenic pets like hairless cats or certain dog breeds known for shedding less dander. Strict hygiene: Maintain a clean environment by regularly vacuuming, washing bedding, and keeping pets off furniture where your child spends a lot of time. Pet care: Train your child on proper handwashing after interacting with pets to minimize allergen transfer to their eyes. Alternatives: Start with low-allergen pets: If unsure about a pet with fur, consider fish or small animals like guinea pigs that can be kept in well-contained environments. Remember: Monitor symptoms: Closely watch your child's VKC symptoms after introducing a pet. If symptoms worsen, consult your doctor to adjust the management plan. Prioritize your child's health: While pets can be wonderful companions, your child's well-being takes precedence. If managing VKC with a pet proves too difficult, prioritize their health and consider alternatives. It's always best to discuss this with your child's doctor for personalized advice based on the severity of their VKC and your living situation.
		I previously provided information on whether your child with vernal keratoconjunctivitis (VKC) can have a pet. Here's a concise summary: Pets and VKC: Potential issue: Pets can introduce allergens like fur, dander that might worsen VKC symptoms, especially if allergies are a trigger. Not an automatic no: If VKC is mild and well-controlled, a pet could be manageable. Before getting a pet: Talk to the ophthalmologist: Discuss your desire for a pet and get their professional opinion based on your child's specific case. Consider hypoallergenic pets: Research options like poodles, certain cat breeds, or even fish with minimal allergens. Prioritize hygiene: Regularly bathe pets, brush them frequently, and maintain a clean environment to minimize allergens. Monitor closely: If VKC symptoms worsen after getting a pet, consult the ophthalmologist again to determine if the pet is a trigger and what adjustments might be needed. Remember, the decision depends on your child's VKC severity. Consulting your doctor and taking precautions can help your child enjoy a pet while managing VKC.
		Whether your child with vernal keratoconjunctivitis (VKC) can have a pet depends on the severity of their VKC and the type of pet. Here's what to consider: Pet Dander: Furry animals like cats, dogs, and rabbits can shed dander, which can irritate the eyes and worsen VKC symptoms. Trigger Identification: If allergens haven't been identified as the cause of your child's VKC, a pet might not be a problem. Discuss this with your child's ophthalmologist. Pet Choice: Consider less allergenic pets like fish or certain breeds of hypoallergenic dogs and cats that shed less dander. Here are some steps you can take: Talk to the Doctor: Discuss your desire to get a pet with your child's ophthalmologist. They can advise based on your child's specific situation. Start Slowly: If getting a pet seems feasible, introduce your child to potential pets in controlled settings to see if their VKC flares up. Pet Care: Teach your child proper hygiene practices around pets, like frequent hand washing and avoiding touching their face after interacting with the animal. Remember, managing VKC often involves minimizing exposure to triggers. Weigh the potential benefits of a pet against the risk of worsening your child's VKC symptoms. Consulting your child's ophthalmologist is crucial before getting a pet.
25	Can you use makeup if you have vernal keratoconjunctivitis?	It's generally not recommended to wear makeup if you have vernal keratoconjunctivitis (VKC) for a few reasons: Potential Irritation: Makeup products, especially eyeliners, mascaras, and eyeshadows, can irritate the already inflamed tissues in your eyes. This can worsen symptoms like itching, redness, and tearing. Risk of Infection: Applying and removing makeup can introduce bacteria or allergens into your eyes, which can lead to infections. This is especially true if you're not careful about hygiene or if your eye condition makes them more susceptible. Flaking and Shedding: VKC can sometimes cause eyelid inflammation and bumps. Makeup can worsen this by flaking off and irritating the already sensitive area. Here's what you can do instead: Focus on Treatment: Prioritize managing your VKC symptoms with medications prescribed by your ophthalmologist. Once your symptoms are under control, you can discuss makeup use with your doctor. Explore Alternatives: If you really want to enhance your eyes, consider hypoallergenic and fragrance-free options like eyeglasses or contact lenses (consult your doctor for suitability with VKC). Consulting Your Doctor: The best course of action is to talk to your ophthalmologist. They can assess the severity of your VKC and advise on whether makeup use is a possibility, and if so, what kind of products might be safest. Remember, prioritizing your eye health is important. While makeup can be fun, it's best to avoid it if it risks worsening your VKC symptoms.
		Wearing makeup with vernal keratoconjunctivitis (VKC) can be tricky, but it's not necessarily impossible. Here's what you need to consider: Potential Irritation: Makeup products can irritate sensitive eyes, especially if you have VKC. Ingredients like fragrances, preservatives, and certain pigments can exacerbate symptoms like itching, redness, and watering. Minimizing Risks: If you do choose to wear makeup, there are ways to minimize the risk of irritation: Hypoallergenic Products: Look for hypoallergenic and fragrance-free makeup specifically designed for sensitive eyes. Minimize Application: Focus on applying makeup minimally, especially around the lash line and inner corners of your eyes. Eyeliner and Mascara: Use these with caution, and consider opting for a kohl pencil instead of liquid liner. Look for fragrance-free mascara formulated for sensitive eyes. Brushes and Sponges: Clean your makeup brushes and applicators regularly to prevent bacteria buildup that can further irritate your eyes. Alternatives: Consider alternatives that might be less irritating, such as eyelash tinting or brow gels. Talk to your doctor: It's always best to consult with your ophthalmologist before using any new products, especially if your VKC is severe. They can advise on the safest approach for your specific situation. Here are some additional tips: Remove makeup gently: Use a gentle, oil-free makeup remover specifically formulated for sensitive eyes. Avoid harsh rubbing or scrubbing. Moisturize: Keeping your eyelids well-moisturized can help reduce irritation. Look for hypoallergenic eye creams suitable for sensitive skin. Avoid Contact Lenses: During VKC flare-ups, it's best to avoid contact lenses and stick to glasses. Remember, prioritizing your eye health is important. If makeup worsens your VKC symptoms, it's best to take a break until your condition improves.
		Using makeup with vernal keratoconjunctivitis (VKC) can be tricky, but it's not entirely out of the question. Here's what you need to consider: Potential Irritation: Makeup products can irritate your eyes, especially if you have VKC. Ingredients like fragrances, preservatives, and certain pigments can exacerbate symptoms like itching, redness, and watering. Eye Health Priority: Your eye health is paramount. If makeup worsens your VKC symptoms, it's best to avoid it or minimize use. Making it Work (if your doctor approves): Hypoallergenic Makeup: Look for hypoallergenic and fragrance-free makeup specifically formulated for sensitive eyes. Minimal Application: Minimize makeup use, especially around the eyes. Opt for a simpler routine and avoid eyeliner, mascara, and eyeshadow close to the lash line. Cleanliness is Key: Practice good hygiene. Wash your makeup brushes regularly with a gentle cleanser and avoid sharing makeup with others. Remove Thoroughly: Gently remove all makeup before bed using a gentle, fragrance-free makeup remover. Alternatives: Focus on Skincare: A healthy skincare routine can enhance your natural beauty and minimize the need for makeup. Lashes and Brows: Consider lash tinting or eyebrow shaping for a more defined look that reduces reliance on makeup. Remember: Consult your doctor: Always discuss using makeup with your doctor, especially if your VKC is severe. They can advise on suitable products and application methods. Individual Tolerance: What works for one person with VKC might irritate another. Experiment cautiously and prioritize your eye comfort. Additional Tips: Look for makeup labeled "non-comedogenic," which means it's less likely to clog pores and irritate your eyes. Use artificial tears throughout the day to soothe and lubricate your eyes, especially if you do wear makeup. By following these tips and consulting your doctor, you can hopefully find a way to enjoy makeup while managing your VKC.
		It's generally not recommended to wear makeup if you have vernal keratoconjunctivitis (VKC) for a few reasons: Irritation: Makeup can irritate the already inflamed eyes, worsening symptoms like itching, redness, and watering. Infection: Applying makeup and removing it can introduce bacteria or allergens into the eyes, potentially leading to infections. Flakes and Particles: Eye makeup, especially mascara and eyeliner, can flake off and get into the eyes, further irritating them. Here's what you can do instead: Focus on eye care: Prioritize treating your VKC with eye drops, medications, and cold compresses as prescribed by your doctor. Hypoallergenic alternatives: Once your VKC is under control, discuss with your ophthalmologist if there are any hypoallergenic makeup options you might be able to try cautiously. Focus on other features: While you can't wear eye makeup, you can highlight other features like your eyebrows or lips with appropriate products if your doctor approves. Remember: Consult your ophthalmologist for personalized advice on makeup use based on your specific VKC condition. There are other ways to enhance your appearance without putting additional strain on your eyes. I hope this information helps!
		It's generally not recommended to wear makeup if you have vernal keratoconjunctivitis (VKC) for a few reasons: Irritation: Makeup products, especially around the eyes (eyeliner, mascara, eyeshadow), can irritate the already inflamed conjunctiva in VKC. This can worsen symptoms like itching, redness, and tearing. Infection Risk: Applying and removing makeup can introduce bacteria or allergens to the eyes, increasing the risk of infection. This is especially true if you have any breaks or injuries on your eyelid. Flares: Certain makeup ingredients might act as irritants for your eyes, even if they don't cause a full-blown allergic reaction. This can trigger VKC flare-ups. Here's what you can do instead: Focus on Skincare: A good skincare routine can enhance your natural beauty without irritating your eyes. Focus on gentle cleansers, moisturizers, and hypoallergenic products. Eyewear: Stylish sunglasses can add a touch of flair while protecting your eyes from irritants and allergens that might worsen VKC. Talk to Your Doctor: Discuss alternative ways to enhance your appearance with your ophthalmologist. They might suggest options that won't irritate your eyes. If you absolutely must wear makeup, here are some precautions to minimize risk: Hypoallergenic Products: Look for makeup labeled "hypoallergenic" and fragrance-free. Minimal Application: Apply minimal makeup, especially around the eyes. Avoid eyeliner, mascara close to the lash line, and eyeshadow directly on the eyelid. Cleanliness: Make sure your makeup brushes and applicators are clean and disinfected regularly. Discard Old Makeup: Throw away any expired makeup products. Remember, prioritizing your eye health is key. Consult your ophthalmologist for personalized advice on makeup use with VKC.

## References

[1] Kang D, Wu H, Yuan L, Shi Y, Jin K, Grzybowski A (2024). A beginner’s guide to artificial intelligence for ophthalmologists. Ophthalmol Ther.

[2] Cheung BH, Lau GK, Wong GT (2023). ChatGPT versus human in generating medical graduate exam multiple choice questions--a multinational prospective study (Hong Kong S.A.R., Singapore, Ireland, and the United Kingdom). PLoS One.

[3] Dahlmann-Noor A, Bonini S, Bremond-Gignac D, Heegaard S, Leonardi A, Montero J, Silva ED (2023). Novel insights in the management of vernal keratoconjunctivitis (VKC): European expert consensus using a modified nominal group technique. Ophthalmol Ther.

[4] Lorenzi A, Pugliese G, Maniaci A (2024). Reliability of large language models for advanced head and neck malignancies management: a comparison between ChatGPT 4 and Gemini Advanced. Eur Arch Otorhinolaryngol.

[5] Rasmussen ML, Larsen AC, Subhi Y, Potapenko I (2023). Artificial intelligence-based ChatGPT chatbot responses for patient and parent questions on vernal keratoconjunctivitis. Graefes Arch Clin Exp Ophthalmol.

[6] Rane N, Choudhary S, Rane J (2024). Gemini versus ChatGPT: applications, performance, architecture, capabilities, and implementation. Journal of Applied Artificial Intelligence.

[7] Potapenko I, Malmqvist L, Subhi Y, Hamann S (2023). Artificial intelligence-based ChatGPT responses for patient questions on optic disc drusen. Ophthalmol Ther.

[8] Alhur A (2024). Redefining healthcare with artificial intelligence (AI): the contributions of ChatGPT, Gemini, and co-pilot. Cureus.

[9] Høj S, Thomsen SF, Meteran H, Sigsgaard T, Meteran H (2024). Artificial intelligence and allergic rhinitis: does ChatGPT increase or impair the knowledge?. J Public Health (Oxf).

[10] Tikhomirov L, Semmler C, McCradden M, Searston R, Ghassemi M, Oakden-Rayner L (2024). Medical artificial intelligence for clinicians: the lost cognitive perspective. Lancet Digit Health.

